# Domino Reactions Enable Zn-Mediated Direct Synthesis of Spiro-Fused 2-Oxindole-α-Methylene-γ-Butyrolactones/Lactams from Isatin Derivatives and 2-(Bromomethyl)acrylates

**DOI:** 10.3390/molecules29153612

**Published:** 2024-07-30

**Authors:** Prathap Reddy Mukthapuram, Amarnath Natarajan

**Affiliations:** Eppley Institute for Research in Cancer and Allied Diseases, Fred & Pamela Buffett Cancer Center, University of Nebraska Medical Center, Omaha, NE 698198, USA

**Keywords:** isatin, spiro-γ-lactones, spiro-γ-lactams, domino strategy, zinc powder

## Abstract

Isatin-derived spirocyclic cores are found in several biologically active molecules. Here, we report nucleophilic domino reactions for the synthesis of α-methylene-γ-butyrolactone/lactam containing spirocyclic oxindoles. The Zn-mediated one-step reaction accommodates a range of substrates and can be used to rapidly generate focused libraries of highly substituted spirocyclic compound.

## 1. Introduction

Domino reactions are a cascade of multiple reactions that occur simultaneously, resulting in the formation of two or more bonds, in a single reaction vessel [1]. Unlike stepwise reactions, domino reactions generate complex products in one pot without the need for the separation and purification of the intermediates. A recent outlook that highlights the above summarizes the use of domino reactions that utilize 1,2-anionotropic rearrangement in the synthesis of complex natural products [2]. The use of metal-catalyzed reactions as part of a domino cascade is another common approach used to generate complex molecules. A recent review by Fan et al. includes the total synthesis of natural products with tricyclic indole cores that employed palladium-catalyzed domino reactions [3].

**Figure 1:**
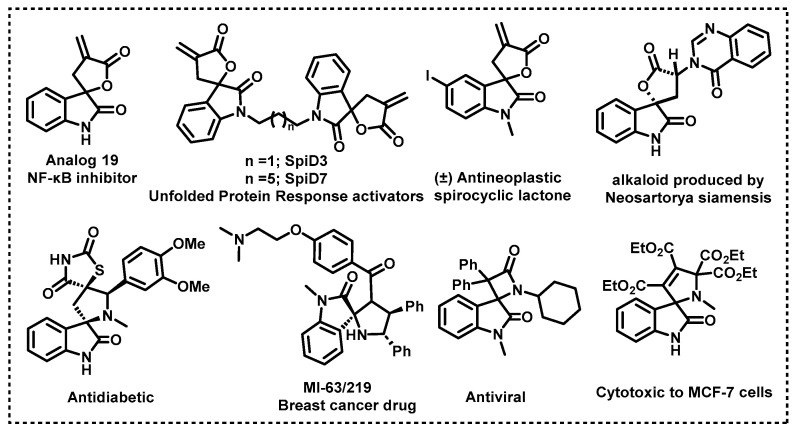
Biologically active molecules containing isatin-based spiro-cyclic lactones and lactams.

Spirocyclic oxindole analogs have attracted substantial attention in recent years due to their prevalence in various natural bioactive compounds [4,5,6,7,8,9,10,11,12,13]. We and others have mounted a variety of substitutions such as α-methylene-γ-butyrolactone/lactam structures on to the spirocyclic oxindole core (Figure 1) and evaluated them as bioactive compounds (e.g., NF-κB inhibitors, anticancer, antimalarial, antiviral, antibacterial, antifungal, and anti-inflammatory compounds) [14,15,16,17,18,19,20,21,22,23,24,25,26,27,28,29,30]. Specifically, we used the α-methylene-γ-butyrolactone on the spiro-isatin core to target cysteine residues on proteins to perturb specific pathways [14,15,16,17,18].

**Scheme 1:**
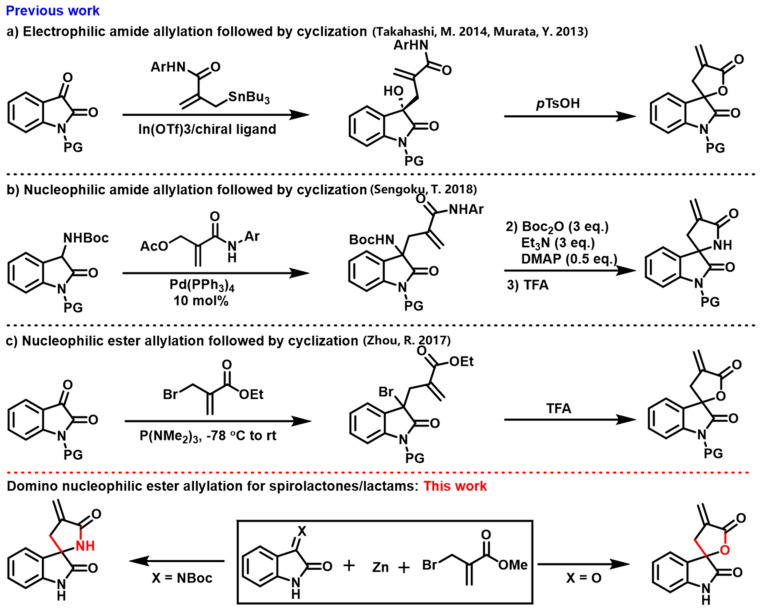
Synthetic approaches to isatin−based spiro−lactones and spiro lactams [31,32,33,34].

Various research groups, including ours, have synthesized spirolactones and spirolactams from a range of starting materials with and without organometallic reagents (Scheme 1). For example, we employed an indium-catalyzed Barbier-type reaction followed by acid-catalyzed cyclization to generate the spirocyclic core. An attractive feature of the α-methylene-γ-butyrolactone-containing spirocyclic systems is the face-selective approach of the biological nucleophile [18,19]. Similarly, enantiopure spirocyclic lactones can be synthesized via a two-step sequence involving the indium-catalyzed nucleophilic amide allylation of N-methyl isatins with allylstannanes. In contrast, spirocyclic lactams were accessed through a three-step reaction sequence involving electrophilic amide allylation using acetoxy methacrylamides and tetrakis(triphenylphosphine)palladium as the catalyst [31,32,33]. A metal catalyst-free approach employed the Umpolung Kukhtin-Ramirez S_N_2-S_N_2 cascade to insert isatin into carbon-halogen bond. The resulting intermediate underwent acid-catalyzed cyclization to generate the spiro oxindole [34]. Another metal catalyst-free approach to access diastereo- and enantio-selective spirolactones is conducted through the use of 3-OBoc-oxindoles as nucleophiles and cinchona alkaloid β-ICD as a catalyst [35].

Nearly every method reported, including those described above, requires multiple steps to access the α,β-unsaturated lactone mounted spiroisatin analogs. Herein, we reported a simple, one-pot metal mediated double nucleophilic domino reactions to synthesize spiro-oxindoles with α-methylene-γ-butyrolactones or lactams from the corresponding isatins or isatinimines as substrates (Scheme 1).

## 2. Results and Discussion

We opted for a Zn-mediated domino strategy for the synthesis of spiro-lactones using isatin (**1a**) and methyl 2-(bromomethyl)acrylate (**2a**). Zn was chosen for its high reactivity, its ease of organozinc formation, and its lower toxicity [36]. A reaction in a sealed tube at 60 °C for 5 h yielded the desired product (**3a**) in modest yields (63%) (Table 1, entry 1). The optimization of reaction conditions, such as temperature, solvents and catalytic amount of an acid, along with reagent ratios identified the optimal conditions that resulted in a 22% increase in the isolated yields (Table 1, entry 4).

**Scheme 2:**
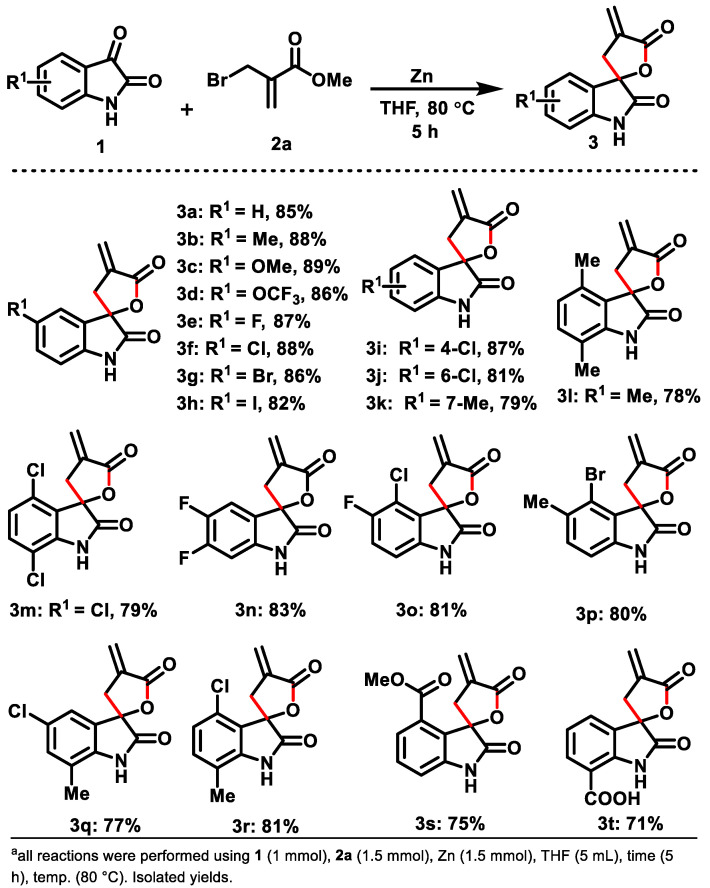
Substrate scope with isatins **1** ^a^.

Under the optimized reaction conditions, we explored the scope of the Zn-mediated spiro-lactone synthesis with mono- and disubstituted isatin analogs **1a**–**1t** (Scheme 2). Regardless of their electronic or steric properties, all isatin analogs afforded the corresponding products **3a**–**3t** in good to excellent yields (78–89%). Notably, substrates with substitutions at the 4-chloro-7-methyl, the 7-carboxylic acid, and the 4-methyl esters yielded the corresponding desired products (**3r**–**3t**) in good to excellent yields. This demonstrates the robustness and broad applicability of the one-pot reaction, which could also be used to generate valuable intermediates for other research applications.

**Scheme 3:**
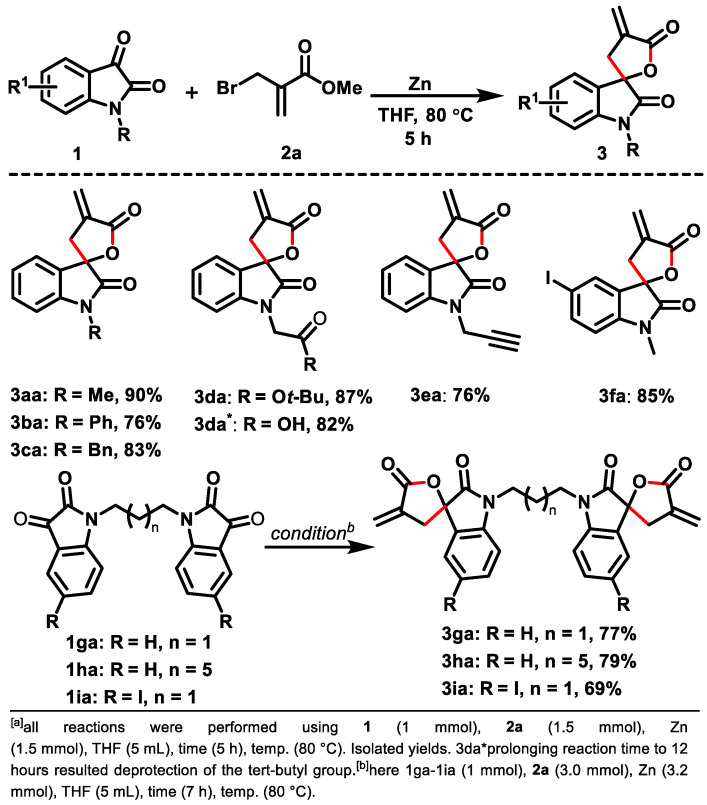
Substrate scope of N-substituted isatins **1** ^a^.

Considering that SpiD3 [14], SpiD7 [15,16], and N-methyl spiro-lactone [37] (Figure 1) function as NF-κB inhibitors and unfolded protein response (UPR) activators with potential anticancer activities, we next explored the effects of various functional groups on the isatin nitrogen atom (Scheme 3). Under the optimized conditions, N-substituted isatin monomers resulted in the corresponding desired products (**3aa**–**3fa**) in good to excellent yields (69–91%). Dimers linked through the isatin nitrogen atom (**1ga**–**1ia**) were subjected to the method reported here. This afforded the desired SpiD3 (**3ga**), SpiD7 (**3ha**) and the 5-iodo dimer (**3ia**) in 77%, 79%, and 85% yields, respectively. Compared with the previous reports, the method reported here not only provided high yields (increasing the overall yield for SpiD3 from 47% to 77% and for SpiD7 from 60% to 79%), but also resulted in the desired product in just one step.

**Scheme 4:**
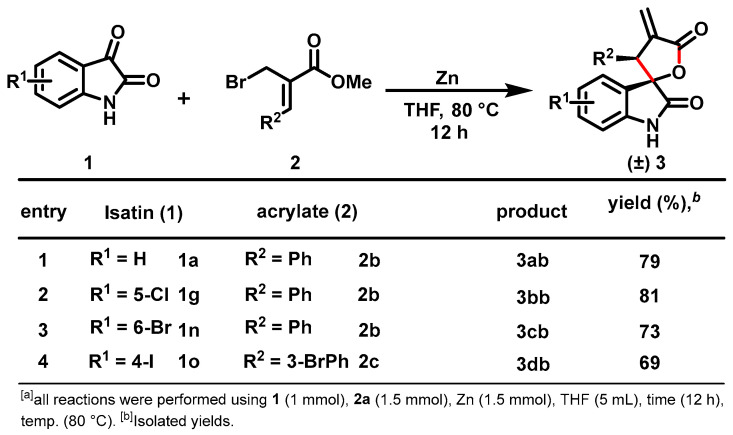
Substrate scope of acrylates **2** on isatins **1** ^a^.

Next, we explored the effect of substitutions on the methyl 2-(bromomethyl) acrylate (Scheme 4). Gratifyingly, arylacrylates such as methyl (E)-2-(bromomethyl)-3-phenylacrylate (**2b**) and methyl 2-(bromomethyl)-3-(3-bromophenyl)acrylate (**2c**), when reacted with isatin (**1a**) or substituted isatins (**1g**, **1n**–**1o**), provided the corresponding lactone products with moderate to good yields and high diastereoselectivity (**3cb**–**3fb**; 69–81%; dr > 99). In alignment with the previous reports [38,39], the stereochemistry of these products (**3**) was determined based on six-membered chair-like transition states. The *re*-*re* and *si*-*si* face attacks were found to be unfavorable due to the axial position of the phenyl group in the indolin-2-one. These attacks produced trans-configured enantiomers, (2S,3S)- and (2R,3R)-spirolactones. In contrast, the *si*-*re* and *re*-*si* face attacks yielded another pair of enantiomers, (2R,3S)- and (2S,3R)-spirolactones, which are cis-configured and more favorable, leading to cis products with respect to the phenyl group of the indolin-2-one and the aryl group of the bromomethyl acrylate. The configuration of the cis stereoisomers was confirmed using NOESY spectroscopy (Supporting Information: Figure S7).

**Scheme 5:**
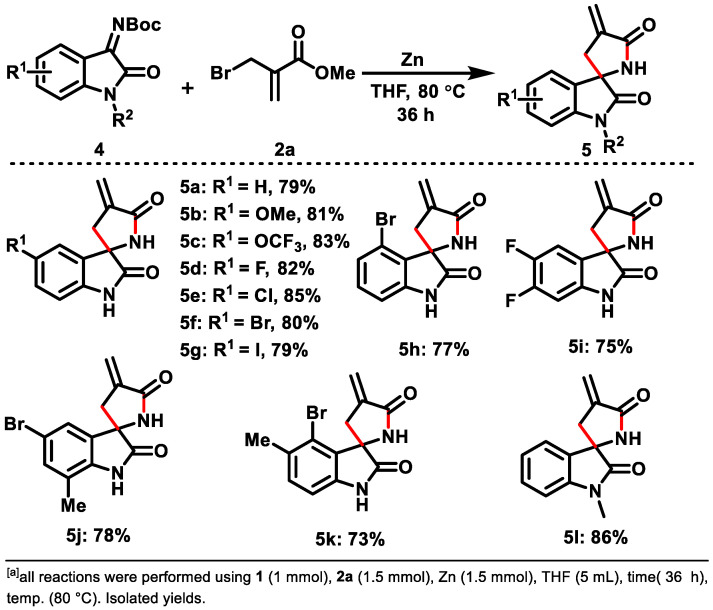
Substrate scope of isatin-derived N-Boc ketimines 4 ^a^.

Next, we explored isatin imine as a substrate and successfully demonstrated that the method can be extended to spiro-lactam synthesis. Notably, under the optimized reaction conditions, isatin imine **4a** was successfully converted to the desired product in 76% yield (Scheme 5; **5a**). Unlike the reaction to generate the spirolactone, it took 36 h for the spirolactam reaction to reach completion. The isolation of the lactam indicates the loss of Boc; however, at this time, we do not know if the cyclization followed the Boc deprotection or the other way around. The efficient formation of the spirolactam prompted us to study the reaction scope with a series of isatin imine substrates **4b**–**4l** (Scheme 5). Like the spirolactone system, regardless of their electronic or steric properties, all substituted isatin analogs explored afforded the corresponding products **5b**–**5l** in good to excellent yields (79–86%).

**Scheme 6:**
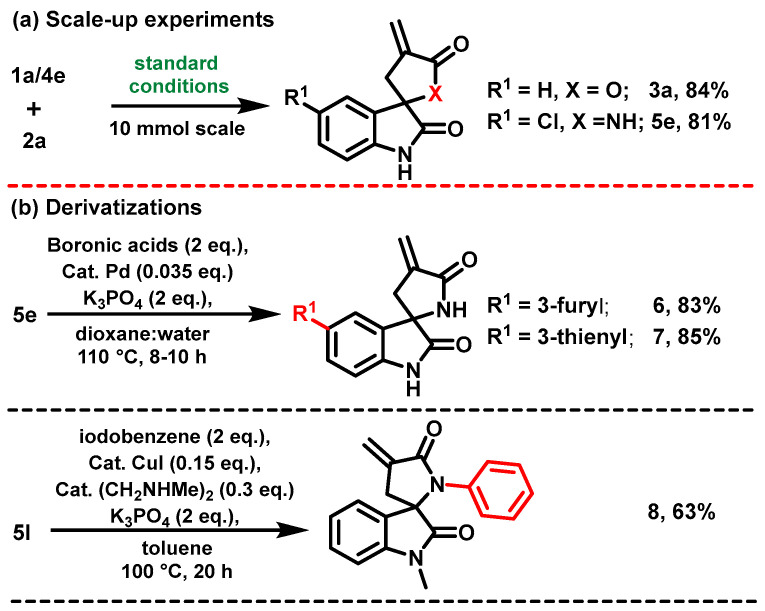
Scale-up experiments and derivatizations of products.

To demonstrate the practical utility of the reported method, preparative scale reactions were performed. The resulting spirolactams (**5e** and **5l**) were used as substrates for the Suzuki reaction and a copper-catalyzed N-arylation reaction (Scheme 6). The model reaction with **1a**/**4e** and methyl 2-(bromomethyl)acrylate **2a** was conducted at 10x scale which resulted in **3a**/**5e** (quantitative yield), demonstrating scalability. We explored diversification following the gram-scale synthesis of **5e** with heteroaryl boronic acids. The reactions reached completion within ~8−10 h and resulted in the corresponding products **6** and **7** in good yields. Coupling of phenyl iodide with 1-methyl-4′-methylenespiro[indoline-3,2′-pyrrolidine]-2,5′-dione (**5l**) afforded N-phenyl α-methylene-γ-lactam **8** in a moderate yield.

**Scheme 7:**
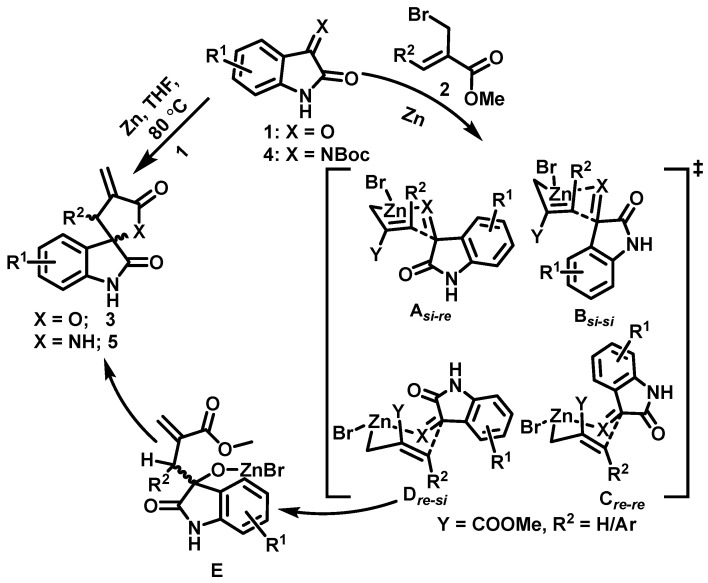
Proposed mechanism.

Based on the experimental observations and previous reports from Lee et al. [38] and the René Csuk group [39], we propose a plausible mechanism for the domino reactions (Scheme 7). The allylation reaction could proceed through four plausible transition states. The *re*-*re* and *si*-*si* attacks are thought to proceed through the six-membered chair-like transition states B and C, respectively, in which the phenyl group of the indolin-2-one occupies an unfavorable axial position, leading to the formation of trans-configurated products. In contrast, the *si*-*re* and *re*-*si* attacks are predicted to occur through the more favorable transition states A and D, respectively, resulting in cis-configurated products with respect to the phenyl group of the indolin-2-one and aryl group. Studies have reported that both *si*-*re* and *re*-*si* attacks in these transition states increase the favorability of the intermediate E. Subsequent nucleophilic addition mediates cyclization, generating the spiro-lactone **3** or spiro-lactam **5**.

## 3. Materials and Methods

^1^H NMR and ^13^C NMR spectra were measured on a Bruker spectrometer, using CDCl_3_ and DMSO-*d* as the solvents with tetramethyl silane (TMS) as an internal standard at room temperature. High-resolution mass spectra (HR-MS) were acquired using an Agilent 6230 LC/TOF mass spectrometer (Agilent technology Co., Ltd. Santa Clara, CA, USA). All solvents used in the experiment were dried using activated molecular sieves, and the other reagents used in the experiment were all analytically pure without any other treatment. Chemical shifts are given in δ relative to TMS, and the coupling constants J are given in Hz. Characterization data of the compounds and NMR spectra of the compounds are given in the Supplementary Materials.

### 3.1. General Method for the Synthesis of Spiro-Fused 2-Oxindole/α-Methylene-γ-Butyrolactone

To zinc (1.5 mmol) in an anhydrous THF solvent (3 mL), substituted isatin (**1**; 1 mmol) and methyl 2-(bromomethyl)acrylate (**2**; 1.5 mmol) were added in sequence in a sealed tube at r.t. under N_2_. The reaction tube was directly sealed and reacted at 80 °C (oil bath temperature) for 5 h and the progress of the reaction was monitored via thin-layer chromatography. Once the reaction was completed, the mixture was cooled to room temperature and quenched via the addition of 1N HCl (2 mL). The resulting mixture was extracted with ethyl acetate (3 × 5 mL) and the combined organic layers were dried over Na_2_SO_4_, filtered, and concentrated under reduced pressure. The crude product was purified by means of flash chromatography on silica gel to give the corresponding product.

### 3.2. General Method for the Synthesis of Spiro-Fused 2-Oxindole/α-Methylene-γ-Butyrolactam

To zinc (1.5 mmol) in an anhydrous THF solvent (3 mL), substituted isatin imines (**4**; 1 mmol) and methyl 2-(bromomethyl)acrylate (**2**; 1.5 mmol) were added in sequence in a sealed tube at r.t. under N_2_. The reaction tube was directly sealed and reacted at 80 °C (oil bath temperature) for 36 h and the progress of the reaction was monitored via thin-layer chromatography. Once the reaction was completed, the mixture was cooled to room temperature and quenched via the addition of 1N HCl (2 mL). The resulting mixture was extracted with ethyl acetate (3 × 5 mL) and the combined organic layers were dried over Na_2_SO_4_, filtered, and concentrated under reduced pressure. The crude product was purified by means of flash chromatography on silica gel to give the corresponding product.

## 4. Conclusions

In summary, we report the development of a one-pot protocol for the synthesis of spiro lactones/lactam derivatives. Isatins or the corresponding imines undergo a Zn-mediated Barbier/aza-Barbier reaction with 2-(bromomethyl)acrylates and the resulting intermediate undergoes cyclization. Key highlights of this study include the use of readily available starting materials to generate a spirocyclic system, this method’s operational simplicity, the use of a non-toxic metal, broad substrate applicability, and high atom efficiency. Additionally, we demonstrated scalability and diversification, indicating that these spirosystems can serve as key intermediates to rapidly generate bioactive libraries. Studies focused on the asymmetric synthesis of spirolactones/lactams are currently underway and will be reported in due course.

## Figures and Tables

**Table 1:** Optimization of reaction conditions *^a^*.

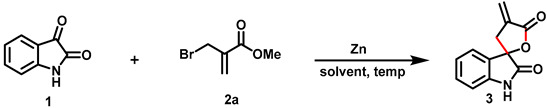					
Entry	2a (equiv.)	Zn (equiv.)	Solvent	Temperature (°C)	3 Yield (%) *^b^*
1	1	1	THF	60	63
2	1	1	THF	80	71
3	1.5	2	THF	80	84
**4**	**1.5**	**1.6**	**THF**	**80**	**85**
5	2.0	2.5	THF	80	81
6	1.5	1.6	DMF	80	63
7	1.5	1.6	Dioxane	80	59
8	1.5	1.6	DCE	80	65
9	1.5	1.6	MeOH	80	73
10	1.5	1.6	THF	70	76
11	1.5	1.6	THF	100	81
12 *^c^*	1.5	1.6	THF	80	82

*^a^* Reaction conditions: **1a** (1 mmol), **2a** (1.5 mmol), Zn (1.6 mmol), THF (5 mL, 80 °C, 5 h, sealed tube. *^b^* Isolated yield. *^c^* Catalytic amount of protonic acid (TfOH: 20 mol%).

## Data Availability

The data underlying this study are available in the published article and its Supplementary Materials.

## Supplementary Materials

The following supporting information can be downloaded at: https://www.mdpi.com/article/10.3390/molecules29153612/s1, Pages S1 and S2: General synthetic procedures for the spirolactones/lactams, Suzuki-Miyaura cross-coupling on spirolactam, and N–arylation of lactams; Figure S1: Transition states for the Barbier reaction in Scheme 4; Figure S2: Numbering of **3bb;** Figure S3: cis structure of **3bb;** Figure S4: trans structure of **3bb**; Figure S5: COSY spectrum of **3bb;** Figure S6: ^1^H-NMR of **3bb**; Figure S7: NOESY spectrum of **3bb**; Pages S7–S16: Characterization data of products; Page S17: References [14,33,40]; Pages S18–S66: NMR spectra of products.
