# Supplementary material for: Domino Reactions Enable Zn-Mediated Direct Synthesis of Spiro-Fused 2-Oxindole-α-Methylene-γ-Butyrolactones/Lactams from Isatin Derivatives and 2-(Bromomethyl)acrylates
Source: Molecules. 2024 Jul 30;29(15):3612. doi: 10.3390/molecules29153612 (PMC11314261; doi:10.3390/molecules29153612)
Supplement: Supplementary file 1 [file molecules-29-03612-s001.zip › molecules-3110323-supplementary.pdf]

Supporting Information

Domino Reactions Enable Zn-Mediated Direct Synthesis of Spiro-Fused 2-Oxindole-α-Methylene-γ-Butyrolactones/lactams from Isatin Derivatives and 2-(Bromomethyl)acrylates

Prathap Reddy Mukthapuram* and Amarnath Natarajan*

Eppley Institute for Research in Cancer and Allied Diseases, Fred & Pamela Buffett Cancer Center, University of Nebraska Medical Center, Omaha, NE 698198, USA.

* Correspondence: pmukthapuram@unmc.edu; anatarajan@unmc.edu; Tel.: +1-(402)-559-3795

1. General Procedure

1.1 General Method for the Synthesis of Spiro-fused 2-oxindole/α-methylene-γ-butyrolactone.

To zinc (1.6 mmol) in anhydrous THF solvent (3 mL), substituted isatin (**1**; 1 mmol) and methyl 2-(bromomethyl)acrylate (**2**; 1.5 mmol) were added in sequence to a seal tube at r.t. under N_2. The reaction tube was directly sealed and reacted at 80 °C (oil bath temperature) for 5 hours and the progress of the reaction was monitored by thin-layer chromatography. Once the reaction was completed, the mixture was cooled to room temperature and quenched by addition of 1N HCl (2 mL). The resulting mixture was extracted with ethyl acetate (3 × 5 mL) and the combined organic layers were dried over Na_2SO_4, filtered, and concentrated under reduced pressure. The crude product was purified by flash chromatography on silica gel to give the corresponding product.

1.2 General Method for the Synthesis of Spiro-fused 2-oxindole/α-methylene-γ-butyrolactam.

To zinc (1.6 mmol) in anhydrous THF solvent (3 mL), substituted isatin imines (**4**; 1 mmol) and methyl 2-(bromomethyl)acrylate (**2**; 1.5 mmol) were added in sequence to a seal tube at r.t. under N_2. The reaction tube was directly sealed and reacted at 80 °C (oil bath temperature) for 36 hours and the progress of the reaction was monitored by thin-layer chromatography. Once the reaction was completed, the mixture was cooled to room temperature and quenched by addition of 1N HCl (2 mL). The resulting mixture was extracted with ethyl acetate (3 × 5 mL) and the combined organic layers were dried over Na_2SO_4, filtered, and concentrated under reduced pressure. The crude product was purified by flash chromatography on silica gel to give the corresponding product.

1.3 General Method for the Suzuki-Miyaura Cross-Coupling of Spirolactam.

An oven-dried sealed tube was charged with the **5a** (1.00 mmol, 1.00 eq.), boronic acid (2.00 mmol, 2.00 eq.), SPhos Pd G2 (0.02-0.035 mmol, 0.02-0.035 eq.), and K_3PO_4 (2.00 mmol, 2.00 eq.). The reaction tube was evacuated and backfilled with argon (this process was repeated a total of three times). Anhydrous dioxane (4 mL) and H_2O (0.8 mL) were then added via syringe, and the sealed tube was placed in a preheated oil bath and stirred at 100 °C for ~8-10 h. The reaction mixture was then cooled to room

temperature and was subsequently filtered through a short plug of Celite, the filter cake washed with EtOAc (25 mL), and the solvent removed under vacuum. The crude product was purified by column chromatography.

1.4 General Procedure for the N-Arylation of Lactams.

An oven-dried sealed tube was charged with the copper(I) iodide (0.15 equiv) and tripotassium phosphate (2 equiv). The pheny iodide (2 equiv) and N,N-dimethylethylenediamine (0.3 equiv) were added to the tube. To this mixture, 1-methyl-4'-methylenespiro[indoline-3,2'-pyrrolidine]-2,5'-dione (**5l**) (1 equiv) was added then degassed by bubbling with argon for 3 times, and this solution was sealed with tube and placed in a preheated oil bath (100 °C). After the complete disappearance of **5l** was observed by TLC, the resulting suspension was allowed to cool to rt and passed through a 1 cm × 1 cm pad of silica gel, eluting with ethyl acetate. The filtrate was concentrated by rotary evaporation, and the residue was purified by silica gel flash column chromatography.

2. Stereochemistry Explanation Based on the Transition states for the Barbier reaction

The stereochemistry of the products (3) was determined based on six-membered chair-like transition states. The configuration of cis stereoisomer was confirmed using NOESY spectroscopy.

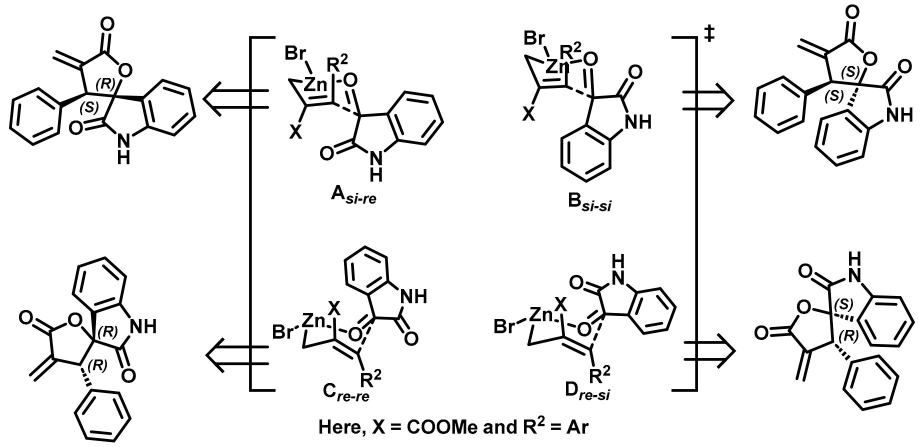

Figure S1: Transition states for the Barbier reaction

- For the *si-si* face attack, the phenyl group is below the plane, and the aryl group is above the plane, resulting in the (2S,3S)-spirolactone product.
- For the *re-re* face attack, the phenyl group is also below the plane, with the aryl group above the plane, leading to the (2R,3R)-spirolactone product.
- For the *si-re* face attack, both the phenyl group and the aryl group are above the plane, resulting in the (2R,3S)-spirolactone product.
- For the *re-si* face attack, both groups are below the plane, leading to the (2S,3R)-spirolactone product.

In summary, the *si-si* and *re-re* face attacks yield a pair of enantiomers, (2S,3S)- and (2R,3R)-spirolactones, which are trans-configured products. The *si-re* and *re-si* face attacks yield another pair of enantiomers, (2R,3S)- and (2S,3R)-spirolactones, which are cis-configured products.

Conclusion:

The *si-re* and *re-si* face attacks are more favorable due to steric factors that influence the transition state during, leading to cis-configurated products with respect to the phenyl group of the indolin-2-one and the aryl group of the bromo methyl acrylate.

Based on the 1D NMR, we can confirm diastereoselectivity. To determine if the configuration is cis or trans, we used 2D NOESY NMR spectroscopy.

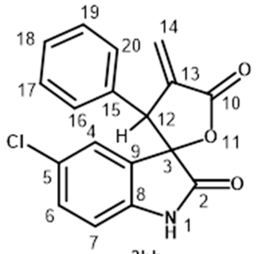

3bb

Figure S2: Numbering for the 3bb structure

In the 3D structure of cis-**3bb**, the distances between H(4) & H(16) and H(4) & H(20) are 2.56 Å and 3.94 Å, respectively. For the trans structure, these distances are 4.43 Å and 5.83 Å. Therefore, a cross-peak between H(4) & H(16) in the NOESY spectrum would be present in the cis structure but absent in the trans configuration. There is a correlation between H(4) & H(16) in NOESY spectrum, indicating the cis configuration. Moreover the lack of a cross peak between H(4) & H(12) which are 2.57 Å apart in the trans configuration also indicates cis-configuration of **3bb**.

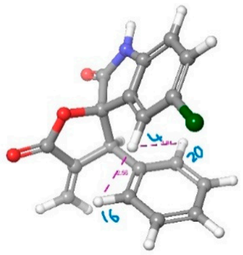

Figure S3: 3D structure of cis-3bb.

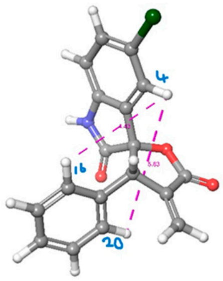

Figure S4: 3D structure of trans-3bb

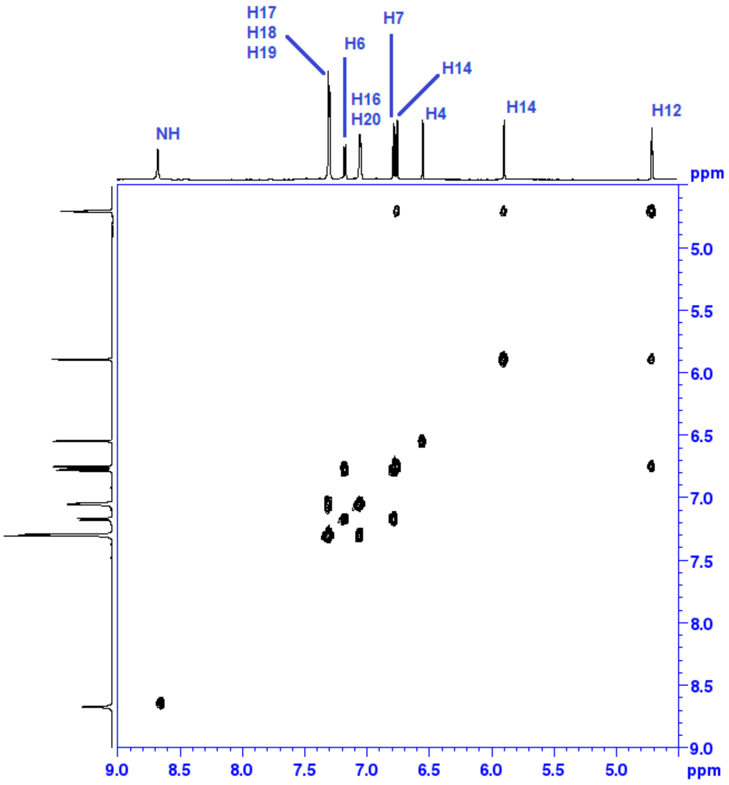

Figure S5: COSY spectrum of compound 3bb at 600 MHz ($CDCl_3$)

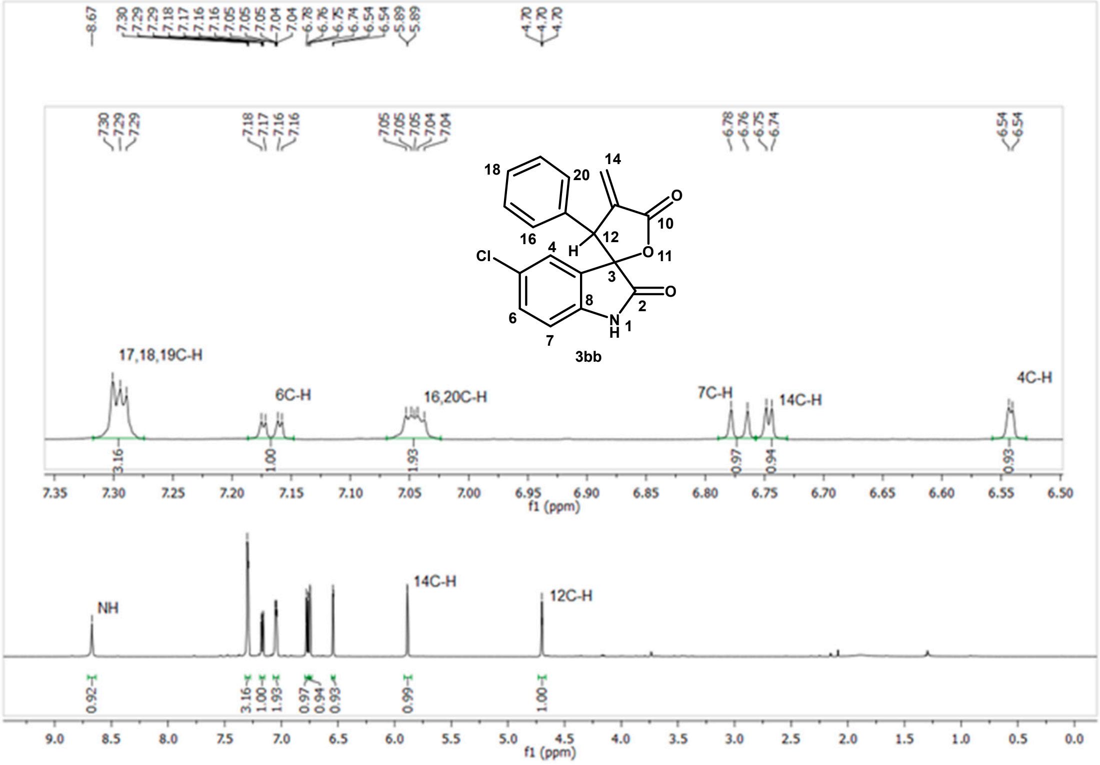

Figure S6: ^{1}H NMR of compound 3bb at 600 MHz ($CDCl_3$)

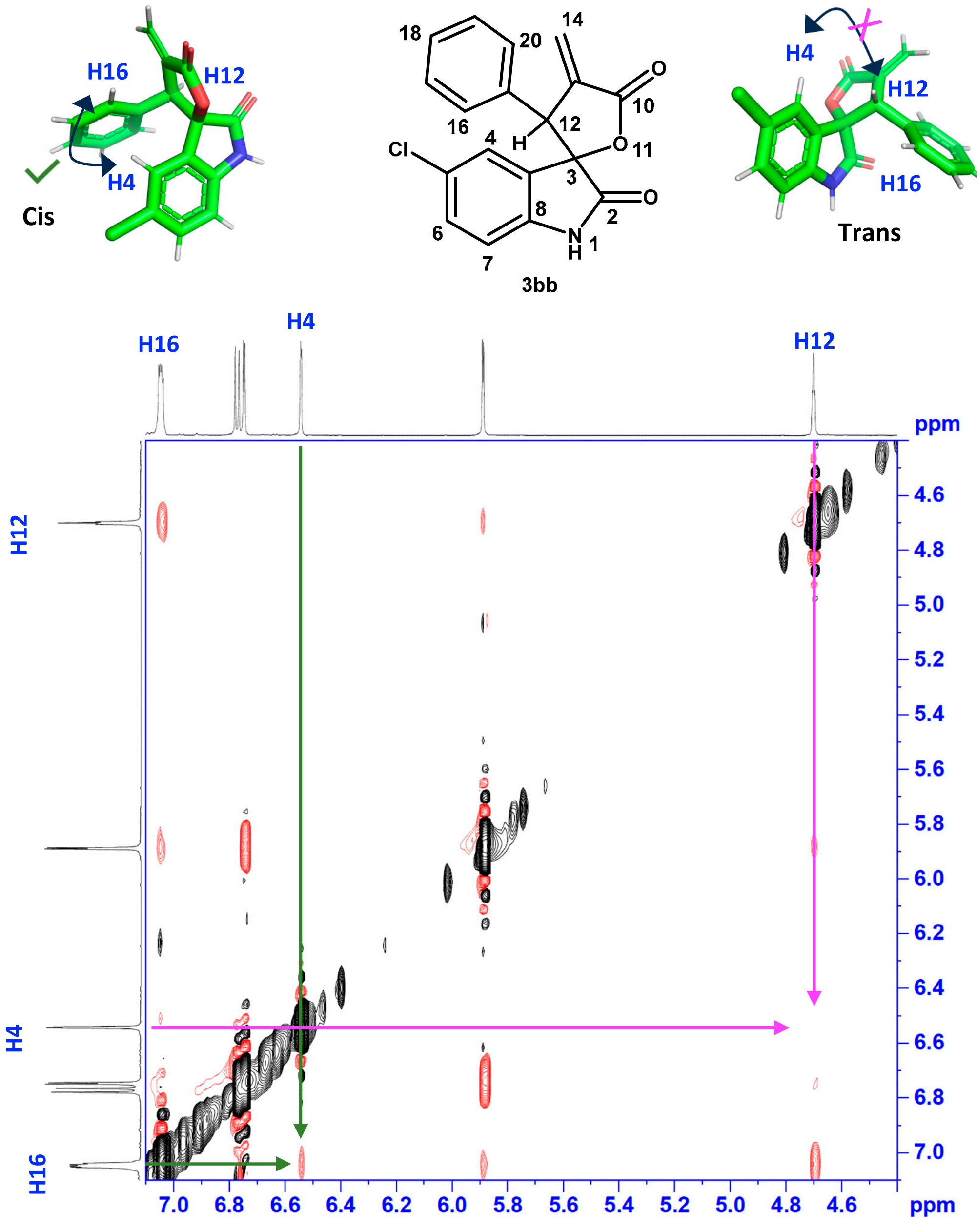

Figure S7: NOESY NMR of compound 3bb at 600 MHz ($CDCl_3$)

3. Characterization data of compounds

4-methylene-3,4-dihydro-5H-spiro[furan-2,3'-indoline]-2',5-dione (3a):[1] White solid, 182.9 mg, yield 85%. m.p. 139–140 °C; ^{1}H NMR (400 MHz, DMSO-*d*$_6$) δ 10.76 (s, 1H), 7.54 (d, *J* = 7.4 Hz, 1H), 7.36 (td, *J* = 7.7, 1.0 Hz, 1H), 7.08 (t, *J* = 7.5 Hz, 1H), 6.92 (d, *J* = 7.8 Hz, 1H), 6.19 (s, 1H), 5.86 (s, 1H), 3.32 – 3.19 (m, 2H). ^{13}C NMR (101 MHz, DMSO-*d*$_6$) δ 175.51, 169.69, 143.00, 134.32, 131.72, 126.91, 125.69, 123.15, 122.49, 110.96, 80.32, 35.88. [M+H]+ Calcd for $C_{12}H_{10}NO_3$ 216.0661; Found 216.0663.

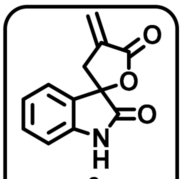

3a

5'-methyl-4-methylene-3,4-dihydro-5H-spiro[furan-2,3'-indoline]-2',5-dione (3b):[1] White solid, 201.7 mg, yield 88%. m.p. 196–197 °C; ^{1}H NMR (400 MHz, DMSO-*d*$_6$) δ 10.65 (s, 1H), 7.35 (s, 1H), 7.16 (d, *J* = 7.9 Hz, 1H), 6.81 (d, *J* = 7.9 Hz, 1H), 6.18 (t, *J* = 2.4 Hz, 1H), 5.86 (bs, 1H), 3.29 – 3.16 (m, 2H), 2.27 (s, 3H). ^{13}C NMR (101 MHz, DMSO-*d*$_6$) δ 175.47, 169.66, 140.45, 134.28, 132.28, 131.86, 126.97, 126.14, 122.53, 110.71, 80.43, 35.88, 21.01. [M+H]+ Calcd for $C_{13}H_{12}NO_3$ 230.0817; Found 230.0809.

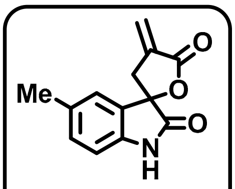

3b

5'-methoxy-4-methylene-3,4-dihydro-5H-spiro[furan-2,3'-indoline]-2',5-dione (3c):[1] White solid, 218.2 mg, yield 89%. m.p. 163–164 °C; ^{1}H NMR (400 MHz, DMSO-*d*$_6$) δ 10.56 (s, 1H), 7.28 (d, *J* = 2.5 Hz, 1H), 6.92 (dd, *J* = 8.5, 2.6 Hz, 1H), 6.83 (d, *J* = 8.5 Hz, 1H), 6.17 (t, *J* = 2.7 Hz, 1H), 5.85 (t, *J* = 2.3 Hz, 1H), 3.73 (s, 3H), 3.25 (t, *J* = 2.4 Hz, 2H). ^{13}C NMR (101 MHz, DMSO-*d*$_6$) δ 175.55, 169.71, 156.05, 135.98, 134.43, 127.92, 122.26, 116.85, 112.24, 111.54, 80.67, 56.10, 35.94. [M+H]+ Calcd for $C_{13}H_{12}NO_4$ 246.0766; Found 246.0776.

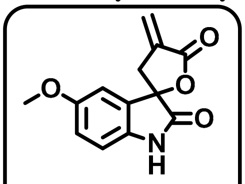

3c

4-methylene-5'-(trifluoromethoxy)-3,4-dihydro-5H-spiro[furan-2,3'-indoline]-2',5-dione (3d): White solid, 257.3 mg, yield 86%. m.p. 126–127 °C; ^{1}H NMR (400 MHz, $CDCl_3$) δ 8.44 (s, 1H), 7.28 – 7.23 (m, 2H), 6.98 (d, *J* = 8.4 Hz, 1H), 6.47 (t, *J* = 2.8 Hz, 1H), 5.87 (t, *J* = 2.4 Hz, 1H), 3.38 (dt, *J* = 17.3, 2.3 Hz, 1H), 3.15 (dt, *J* = 17.4, 2.9 Hz, 1H). ^{13}C NMR (101 MHz, $CDCl_3$) δ 175.82, 168.71, 145.33, 139.71, 131.88, 128.27, 124.60, 124.05, 120.41 (q, *J* = 257.8), 118.43, 111.97, 79.50, 36.25. HRMS (ESI-TOF) m/z: $[M+H]^+$ Calcd for $C_{13}H_9F_3NO_4$ 300.0484; Found 300.0494.

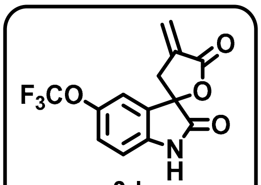

3d

5'-fluoro-4-methylene-3,4-dihydro-5H-spiro[furan-2,3'-indoline]-2',5-dione (3e):[1] Yellow solid, 202.9 mg, yield 87%. m.p. 164–165 °C; ^{1}H NMR (400 MHz, DMSO-d_6) δ 10.78 (s, 1H), 7.58 (dd, J = 8.1, 2.5 Hz, 1H), 7.21 (td, J = 9.3, 2.6 Hz, 1H), 6.91 (dd, J = 8.5, 4.3 Hz, 1H), 6.18 (bs, 1H), 5.86 (bs, 1H), 3.27 (bs, 2H). ^{13}C NMR (101 MHz, DMSO-d_6) δ 175.60, 169.53, 158.82 (d, J = 238.4), 139.21, 134.11, 128.45 (d, J = 8.4), 122.54, 118.10 (d, J = 23.5), 113.79 (d, J = 25.5), 111.98 (d, J = 8.1), 80.31, 35.77. [M+H]+ Calcd for $C_{12}H_9FNO_3$ 234.0566; Found 234.0575.

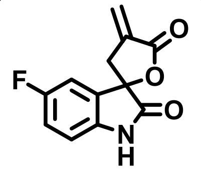

3e

5'-chloro-4-methylene-3,4-dihydro-5H-spiro[furan-2,3'-indoline]-2',5-dione (3f):[1] White solid, 219.7 mg, yield 88%. m.p. 190–191 °C; ^{1}H NMR (400 MHz, DMSO-d_6) δ 10.88 (s, 1H), 7.75 (d, J = 2.1 Hz, 1H), 7.42 (dd, J = 8.3, 2.2 Hz, 1H), 6.93 (d, J = 8.3 Hz, 1H), 6.18 (t, J = 2.7 Hz, 1H), 5.86 (t, J = 2.2 Hz, 1H), 3.28 – 3.27 (m, 2H). ^{13}C NMR (101 MHz, DMSO-d_6) δ 175.33, 169.48, 141.96, 134.08, 131.51, 128.83, 127.12, 126.17, 122.54, 112.47, 80.08, 35.71. [M+H]+ Calcd for $C_{12}H_9ClNO_3$ 250.0271; Found 250.0259.

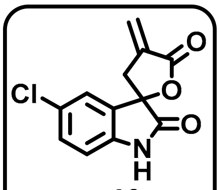

3f

H

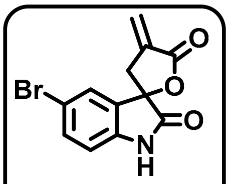

3g

5'-bromo-4-methylene-3,4-dihydro-5H-spiro[furan-2,3'-indoline]-2',5-dione (3g):[1] White solid, 252.9 mg, yield 86%. m.p. 195–196 °C; ^{1}H NMR (400 MHz, DMSO-d_6) δ 10.89 (s, 1H), 7.86 (s, 1H), 7.55 (d, J = 7.5 Hz, 1H), 6.88 (d, J = 8.1 Hz, 1H), 6.18 (s, 1H), 5.85 (s, 1H), 3.27 (s, 2H). ^{13}C NMR (101 MHz, DMSO-d_6) δ 175.21, 169.48, 142.38, 134.36, 134.09, 129.20, 128.89, 122.53, 114.69, 112.95, 80.03, 35.70. [M+H]+ Calcd for $C_{12}H_9BrNO_3$ 293.9766; Found 293.9739.

5'-iodo-4-methylene-3,4-dihydro-5H-spiro[furan-2,3'-indoline]-2',5-dione (3h):[1] White solid, 279.7 mg, yield 82%. m.p. 179–180 °C; ^{1}H NMR (400 MHz, DMSO-d_6) δ 10.86 (s, 1H), 7.97 (d, J = 1.5 Hz, 1H), 7.70 (dd, J = 8.2, 1.6 Hz, 1H), 6.76 (d, J = 8.2 Hz, 1H), 6.17 (t, J = 2.6 Hz, 1H), 5.85 (bs, 1H), 3.26 (bs, 2H). ^{13}C NMR (101 MHz, DMSO-d_6) δ 175.00, 169.50, 140.12, 134.26, 134.12, 129.41, 122.48, 113.34, 85.82, 79.88, 35.69. [M+H]+ Calcd for $C_{12}H_9INO_3$ 341.9627; Found 341.9645.

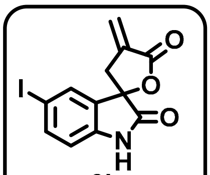

3h

H

4'-chloro-4-methylene-3,4-dihydro-5H-spiro[furan-2,3'-indoline]-2',5-dione (3i):[1] White solid, 217.2 mg, yield 87%. m.p. 166–167 °C; ^{1}H NMR (400 MHz, DMSO-d_6) δ 11.06 (s, 1H), 7.40 (t, J = 8.0 Hz, 1H), 7.12 (d, J = 8.2 Hz, 1H), 6.92 (d, J = 7.8 Hz, 1H), 6.27 (t, J = 2.9 Hz, 1H), 5.95 (t, J = 2.5 Hz, 1H), 3.42 – 3.35 (m, 1H), 3.29 (dt, J = 18.5, 2.5 Hz, 1H). ^{13}C NMR (101 MHz, DMSO-d_6) δ 174.69, 169.34, 144.94, 133.52, 133.29, 131.07, 123.95, 123.55, 110.25, 80.05, 33.08. [M+H]+ Calcd for $C_{12}H_9ClNO_3$ 250.0271; Found 250.0270.

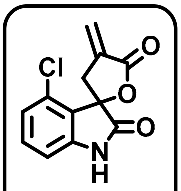

3i

6'-chloro-4-methylene-3,4-dihydro-5H-spiro[furan-2,3'-indoline]-2',5-dione (3j):[1] White solid, 202.2 mg, yield 81%. m.p. 167–168 °C; ^{1}H NMR (400 MHz, DMSO-d_6) δ 10.92 (s, 1H), 7.60 (d, J = 8.0 Hz, 1H), 7.14 (dd, J = 8.0, 1.9 Hz, 1H), 6.94 (d, J = 1.8 Hz, 1H), 6.19 (t, J = 2.7 Hz, 1H), 5.87 (t, J = 2.4 Hz, 1H), 3.27 – 3.20 (m, 2H). ^{13}C NMR (101 MHz, DMSO-d_6) δ 175.49, 169.53, 144.58, 135.97, 134.10, 127.37, 125.78, 122.90, 122.64, 111.12, 79.80, 35.74. [M+H]+ Calcd for $C_{12}H_9ClNO_3$ 250.0271; Found 250.0284.

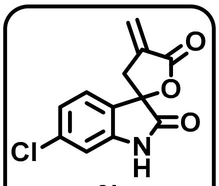

3j

7'-methyl-4-methylene-3,4-dihydro-5H-spiro[furan-2,3'-indoline]-2',5-dione (3k):[1] White solid, 181.1 mg, yield 79%. m.p. 205–206 °C; ^{1}H NMR (400 MHz, DMSO-d_6) δ 10.81 (s, 1H), 7.35 (d, J = 7.3 Hz, 1H), 7.18 (d, J = 7.6 Hz, 1H), 6.99 (t, J = 7.5 Hz, 1H), 6.18 (bs, 1H), 5.86 (bs, 1H), 3.29 – 3.18 (m, 2H), 2.22 (s, 3H). ^{13}C NMR (101 MHz, DMSO-d_6) δ 176.01, 169.71, 141.51, 134.39, 132.87, 126.63, 123.12, 122.85, 122.42, 120.48, 80.59, 36.02, 16.66. [M+H]+ Calcd for $C_{13}H_{12}NO_3$ 230.0817; Found 230.0808.

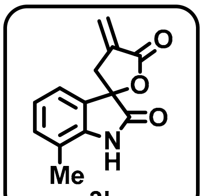

3k

4',7'-dimethyl-4-methylene-3,4-dihydro-5H-spiro[furan-2,3'-indoline]-2',5-dione (3l): Yellow solid, 189.7 mg, yield 78%. m.p. 206–207 °C; ^{1}H NMR (400 MHz, DMSO-d_6) δ 10.77 (s, 1H), 7.07 (d, J = 7.8 Hz, 1H), 6.78 (d, J = 7.8 Hz, 1H), 6.26 (t, J = 2.5 Hz, 1H), 5.92 (bs, 1H), 3.31 – 3.22 (m, 2H), 2.18 (s, 3H), 2.15 (s, 3H). ^{13}C NMR (101 MHz, DMSO-d_6) δ 176.02, 169.69, 141.38, 133.75, 133.34, 132.68, 125.04, 124.03, 123.68, 117.98, 81.05, 33.82, 17.10, 16.38. HRMS (ESI-TOF) m/z: $[M+H]^+$ Calcd for $C_{14}H_{14}NO_3$ 244.0974; Found 244.0975.

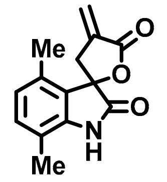

3l

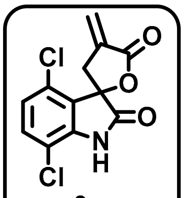

3m

4',7'-dichloro-4-methylene-3,4-dihydro-5H-spiro[furan-2,3'-indoline]-2',5-dione (3m): Half White solid, 224.4 mg, yield 79%. m.p. 198–199 °C; ^{1}H NMR (400 MHz, DMSO-d_6) δ 11.53 (s, 1H), 7.51 (d, J = 8.8 Hz, 1H), 7.16 (d, J = 8.8 Hz, 1H), 6.28 (bs, 1H), 5.98 (bs, 1H), 3.45 – 3.34 (m, 2H). ^{13}C NMR (101 MHz, DMSO-d_6) δ 174.76, 169.16, 142.61, 133.11, 132.92, 129.67, 125.27, 124.66, 124.31, 114.45, 80.47, 33.14. HRMS (ESI-TOF) m/z: $[M+H]^+$ Calcd for $C_{12}H_8Cl_2NO_3$ 283.9881; Found 283.9890.

5',6'-difluoro-4-methylene-3,4-dihydro-5H-spiro[furan-2,3'-indoline]-2',5-dione (3n): White solid, 208.5 mg, yield 83%. m.p. 191–192 °C; ^{1}H NMR (400 MHz, DMSO-d_6) δ 10.89 (s, 1H), 7.90 – 7.80 (m, 1H), 6.98 (dd, J = 10.4, 6.6 Hz, 1H), 6.18 (t, J = 2.4 Hz, 1H), 5.86 (bs, 1H), 3.26 – 3.25 (m, 2H). ^{13}C NMR (101 MHz, DMSO-d_6) δ 175.67, 169.42, 151.69 (dd, J = 249.3, 13.9), 146.25 (dd, J = 240.4, 13.4), 140.01 (dd, J = 10.7, 2.3), 134.03, 122.79 (dd, J = 7.0, 3.6), 122.59, 115.99 (d, J = 20.9), 101.11 (d, J = 22.8), 79.92, 35.66. HRMS (ESI-TOF) m/z: $[M+H]^+$ Calcd for $C_{12}H_8F_2NO_3$ 252.0472; Found 252.0438.

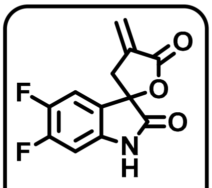

3n

4'-chloro-5'-fluoro-4-methylene-3,4-dihydro-5H-spiro[furan-2,3'-indoline]-2',5-dione (3o): Light yellow solid, 216.3 mg, yield 81%. m.p. 149–150 °C; ^{1}H NMR (400 MHz, $CDCl_3$) δ 8.72 (s, 1H), 7.17 (t, *J* = 8.8 Hz, 1H), 6.85 (dd, *J* = 8.6, 3.6 Hz, 1H), 6.47 (t, *J* = 2.9 Hz, 1H), 5.87 (t, *J* = 2.6 Hz, 1H), 3.46 (dt, *J* = 17.7, 2.9 Hz, 1H), 3.31 (dt, *J* = 17.7, 2.6 Hz, 1H). ^{13}C NMR (101 MHz, $CDCl_3$) δ 175.35, 168.71, 155.00 (d, *J* = 246.4), 137.95, 132.00, 125.46, 123.95, 120.20 (d, *J* = 21.6), 118.67 (d, *J* = 23.1), 109.99 (d, *J* = 6.8), 79.90, 33.13. HRMS (ESI-TOF) m/z: $[M+H]^+$ Calcd for $C_{12}H_8ClFNO_3$ 268.0177; Found 268.0179.

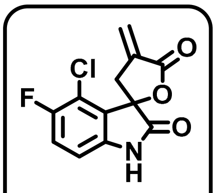

3o

4'-bromo-5'-methyl-4-methylene-3,4-dihydro-5H-spiro[furan-2,3'-indoline]-2',5-dione (3p): White solid, 246.5 mg, yield 80%. m.p. 199–200 °C; ^{1}H NMR (400 MHz, DMSO-d_6) δ 10.93 (s, 1H), 7.36 (d, *J* = 7.9 Hz, 1H), 6.87 (d, *J* = 7.9 Hz, 1H), 6.27 (t, *J* = 2.7 Hz, 1H), 5.94 (bs, 1H), 3.42 – 3.36 (m, 1H), 3.24 (m, 1H), 2.30 (s, 3H). ^{13}C NMR (101 MHz, DMSO-d_6) δ 174.86, 169.57, 142.75, 133.63, 133.58, 132.01, 125.59, 123.77, 122.11, 110.45, 80.96, 32.98, 21.74. HRMS (ESI-TOF) m/z: $[M+H]^+$ Calcd for $C_{13}H_{11}BrNO_3$ 307.9922; Found 307.9969.

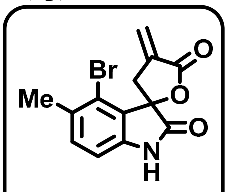

3p

5'-chloro-7'-methyl-4-methylene-3,4-dihydro-5H-spiro[furan-2,3'-indoline]-2',5-dione (3q): Half White solid, 203.0 mg, yield 77%. m.p. 211–212 °C; ^{1}H NMR (400 MHz, DMSO-d_6) δ 10.94 (s, 1H), 7.55 (s, 1H), 7.29 (s, 1H), 6.18 (s, 1H), 5.85 (s, 1H), 3.26 (bs, 2H), 2.22 (s, 3H). ^{13}C NMR (101 MHz, DMSO-d_6) δ 175.81, 169.52, 140.61, 134.14, 132.34, 128.33, 126.97, 123.24, 122.66, 122.48, 80.29, 35.83, 16.48. HRMS (ESI-TOF) m/z: $[M+H]^+$ Calcd for $C_{13}H_{11}ClNO_3$ 264.0427; Found 264.0464.

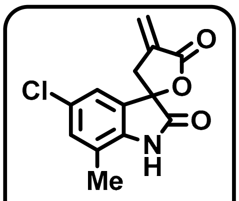

3q

4'-chloro-7'-methyl-4-methylene-3,4-dihydro-5H-spiro[furan-2,3'-indoline]-2',5-dione (3r): White solid, 213.6 mg, yield 81%. m.p. 196–197 °C; ^{1}H NMR (400 MHz, DMSO-d_6) δ 11.08 (s, 1H), 7.24 – 7.22 (m, 1H), 7.03 (d, *J* = 8.3 Hz, 1H), 6.26 (t, *J* = 3.0 Hz, 1H), 5.95 (t, *J* = 2.6 Hz, 1H), 3.39 – 3.38 (m, 1H), 3.28 (dt, *J* = 18.3, 2.6 Hz, 1H), 2.21 (s, 3H). ^{13}C NMR (101 MHz, DMSO-d_6) δ 175.22, 169.38, 143.37, 134.48, 133.39, 128.15, 123.84, 123.33, 123.22, 120.01, 80.34, 33.16, 16.33. HRMS (ESI-TOF) m/z: $[M+H]^+$ Calcd for $C_{13}H_{11}ClNO_3$ 264.0427; Found 264.0464.

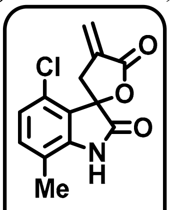

3r

methyl 4-methylene-2',5-dioxo-4,5-dihydro-3H-spiro[furan-2,3'-indoline]-4'-carboxylate (3s): White solid, 204.9 mg, yield 75%. m.p. 156–157 °C; ^{1}H NMR (400 MHz, DMSO-d_6) δ 11.02 (s, 1H), 7.65 (dd, *J* = 8.0, 1.0 Hz, 1H), 7.54 (t, *J* = 7.9 Hz, 1H), 7.21 (dd, *J* = 7.8, 1.0 Hz, 1H), 6.20 (t, *J* = 2.9 Hz, 1H), 5.82 (t, *J* = 2.5 Hz, 1H), 3.76 (s, 3H), 3.39 – 3.34 (m, 1H), 3.18 (dt, *J* = 17.9, 2.4 Hz, 1H). ^{13}C NMR (101 MHz, DMSO-d_6) δ 175.87, 170.12, 165.05, 144.28, 134.80, 132.05, 127.73, 127.59, 124.78, 121.98, 115.77, 80.17, 53.02, 34.74. HRMS (ESI-TOF) m/z: [M+H]+ Calcd for $C_{14}H_{12}NO_5$ 274.0715; Found 274.0756.

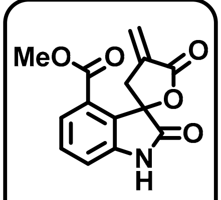

3s

4-methylene-2',5-dioxo-4,5-dihydro-3H-spiro[furan-2,3'-indoline]-7'-carboxylic acid (3t): White solid, 184.0 mg, yield 71%. m.p. 223–224 °C; ^{1}H NMR (400 MHz, DMSO-d_6) δ 13.54 (bs, 1H), 10.39 (s, 1H), 7.85 (d, J = 7.8 Hz, 1H), 7.78 (d, J = 6.9 Hz, 1H), 7.17 (t, J = 7.7 Hz, 1H), 6.21 (bs, 1H), 5.89 (s, 1H), 3.30 (bs, 2H). ^{13}C NMR (101 MHz, DMSO-d_6) δ 175.45, 175.40, 169.56, 144.12, 134.07, 132.37, 129.76, 128.23, 126.41, 122.93, 122.78, 79.22, 35.84. HRMS (ESI-TOF) m/z: [M+H]+ Calcd for $C_{13}H_{10}NO_5$ 260.0559; Found 260.0597.

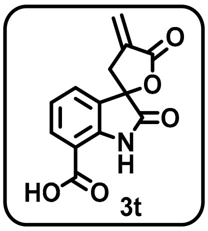

1'-methyl-4-methylene-3,4-dihydro-5H-spiro[furan-2,3'-indoline]-2',5-dione (3aa):[1] White solid, 206.3 mg, yield 90%. m.p. 133–134 °C; ^{1}H NMR (400 MHz, CDCl$_3$) δ 7.42 (td, J = 7.8, 1.1 Hz, 1H), 7.35 – 7.33 (m, 1H), 7.14 (t, J = 7.6 Hz, 1H), 6.90 (d, J = 7.8 Hz, 1H), 6.43 (t, J = 2.8 Hz, 1H), 5.82 (t, J = 2.5 Hz, 1H), 3.32 (dt, J = 17.2, 2.5 Hz, 1H), 3.23 (s, 3H), 3.12 (dt, J = 17.3, 2.9 Hz, 1H). ^{13}C NMR (101 MHz, CDCl$_3$) δ 173.47, 168.98, 143.91, 132.80, 131.31, 126.71, 124.13, 123.67, 123.13, 108.97, 79.35, 36.27, 26.49. [M+H]+ Calcd for $C_{13}H_{12}NO_3$ 230.0817; Found 230.0826.

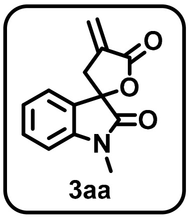

4-methylene-1'-phenyl-3,4-dihydro-5H-spiro[furan-2,3'-indoline]-2',5-dione (3ba):[1] White solid, 221.4 mg, yield 76%. m.p. 138–139 °C; ^{1}H NMR (400 MHz, DMSO-d_6) δ 7.73 (d, J = 7.5 Hz, 1H), 7.63 – 7.57 (m, 2H), 7.53 – 7.47 (m, 3H), 7.41 (t, J = 7.8 Hz, 1H), 7.22 (t, J = 7.5 Hz, 1H), 6.81 (d, J = 7.9 Hz, 1H), 6.23 (bs, 1H), 5.92 (bs, 1H), 3.53 – 3.48 (m, 1H), 3.40 (t, J = 2.9 Hz, 1H). ^{13}C NMR (101 MHz, DMSO-d_6) δ 172.84, 169.14, 143.90, 133.67, 133.30, 131.38, 129.72, 128.54, 126.64, 125.74, 125.46, 123.89, 122.35, 109.75, 79.67, 35.82. [M+H]+ Calcd for $C_{18}H_{14}NO_3$ 292.0974; Found 292.0964.

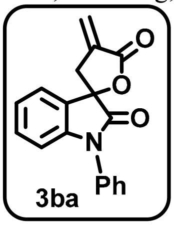

1'-benzyl-4-methylene-3,4-dihydro-5H-spiro[furan-2,3'-indoline]-2',5-dione (3ca):[1] White solid, 253.4 mg, yield 83%. m.p. 145–146 °C; ^{1}H NMR (400 MHz, CDCl$_3$) δ 7.38 – 7.30 (m, 7H), 7.11 (t, J = 7.6 Hz, 1H), 6.78 (d, J = 7.8 Hz, 1H), 6.46 (t, J = 2.8 Hz, 1H), 5.85 (t, J = 2.4 Hz, 1H), 4.91 (m, 2H), 3.39 (dt, J = 17.2, 2.4 Hz, 1H), 3.18 (dt, J = 17.2, 2.9 Hz, 1H). ^{13}C NMR (101 MHz, CDCl$_3$) δ 173.70, 168.99, 143.05, 134.86, 132.77, 131.21, 128.98, 127.98, 127.30, 126.69, 124.26, 123.70, 123.25, 110.01, 79.38, 77.35, 44.06, 36.40. [M+H]+ Calcd for $C_{19}H_{16}NO_3$ 306.1130; Found 306.1126.

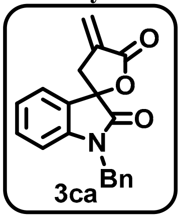

Tert-butyl 2-(4-methylene-2',5-dioxo-4,5-dihydro-3H-spiro[furan-2,3'-indolin]-1'-yl)acetate (3da): White solid, 286.5 mg, yield 87%. m.p. 104–105 °C; ^{1}H NMR (400 MHz, CDCl$_3$) δ 7.42 – 7.35 (m, 2H), 7.16 (t, J = 7.6 Hz, 1H), 6.77 (d, J = 7.9 Hz, 1H), 6.44 (t, J = 2.8 Hz, 1H), 5.82 (t, J = 2.4 Hz, 1H), 4.53 (d, J = 17.5 Hz, 1H), 4.20 (d, J = 17.5 Hz, 1H), 3.37 (dt, J = 17.3, 2.3 Hz, 1H), 3.16 (dt, J = 17.3, 2.8 Hz, 1H), 1.47 (s, 9H). ^{13}C NMR (101 MHz, CDCl$_3$) δ 173.57, 168.96, 165.93, 142.75, 132.67, 131.21, 126.44, 124.31, 123.92, 123.23, 109.01, 83.14, 79.30, 42.23, 36.60, 27.95. HRMS (ESI-TOF) m/z: [M+Na]$^+$ Calcd for $C_{18}H_{19}NO_5Na$ 352.1161; Found 352.1193.

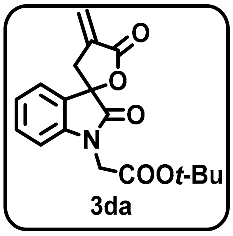

2-(4-methylene-2',5-dioxo-4,5-dihydro-3H-spiro[furan-2,3'-indolin]-1'-yl)acetic acid (3da*): Semi solid, 224.1 mg, yield 82%. ^{1}H NMR (400 MHz, DMSO-d_6) δ 13.23 (s, 1H), 7.64 (d, *J* = 7.0 Hz, 1H), 7.44 (td, *J* = 7.8, 1.0 Hz, 1H), 7.18 – 7.12 (m, 2H), 6.22 (t, *J* = 2.7 Hz, 1H), 5.90 (t, *J* = 2.3 Hz, 1H), 4.47 (s, 2H), 3.32 (dt, *J* = 17.4, 3.1 Hz, 1H), 3.25 – 3.20 (m, 1H). ^{13}C NMR (101 MHz, DMSO-d_6) δ 174.07 (s), 169.55 (s), 169.26 (s), 143.61 (s), 133.96 (s), 131.71 (s), 126.03 (s), 125.48 (s), 123.94 (s), 122.93 (s), 110.23 (s), 79.79 (s), 41.65 (s), 36.20 (s). HRMS (ESI-TOF) m/z: $[M+H]^+$ Calcd for $C_{14}H_{12}NO_5$ 274.0715; Found 274.0739.

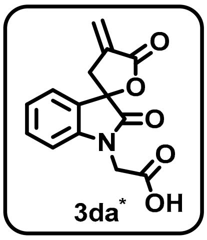

4-methylene-1'-(prop-2-yn-1-yl)-3,4-dihydro-5H-spiro[furan-2,3'-indoline]-2',5-dione (3ea):[1] White solid, 192.5 mg, yield 76%. m.p. 172–173 °C; ^{1}H NMR (400 MHz, $CDCl_3$) δ 7.46 (t, *J* = 7.8 Hz, 1H), 7.37 (d, *J* = 7.4 Hz, 1H), 7.19 (t, *J* = 7.6 Hz, 1H), 7.13 (d, *J* = 7.9 Hz, 1H), 6.45 (t, *J* = 2.8 Hz, 1H), 5.83 (t, *J* = 2.4 Hz, 1H), 4.60 (dd, *J* = 17.7, 2.5 Hz, 1H), 4.43 (dd, *J* = 17.7, 2.4 Hz, 1H), 3.35 (dt, *J* = 17.3, 2.3 Hz, 1H), 3.15 (dt, *J* = 17.3, 2.8 Hz, 1H), 2.31 (t, *J* = 2.4 Hz, 1H). ^{13}C NMR (101 MHz, $CDCl_3$) δ 172.60, 168.80, 142.02, 132.55, 131.29, 126.55, 124.27, 124.06, 123.36, 110.07, 79.27, 76.02, 73.12, 36.35, 29.67. [M+H]+ Calcd for $C_{15}H_{12}NO_3$ 254.0817; Found 254.0841.

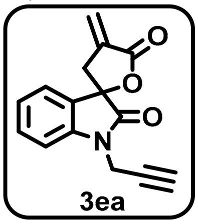

5'-iodo-1'-methyl-4-methylene-3,4-dihydro-5H-spiro[furan-2,3'-indoline]-2',5-dione (3fa):[1] White solid, 301.9 mg, yield 85%. m.p. 208–209 °C; ^{1}H NMR (400 MHz, $CDCl_3$) δ 7.75 (dd, *J* = 8.2, 1.7 Hz, 1H), 7.61 (d, *J* = 1.7 Hz, 1H), 6.69 (d, *J* = 8.2 Hz, 1H), 6.45 (t, *J* = 2.8 Hz, 1H), 5.84 (t, *J* = 2.5 Hz, 1H), 3.32 (dt, *J* = 17.3, 2.5 Hz, 1H), 3.22 (s, 3H), 3.11 (dt, *J* = 17.3, 2.8 Hz, 1H). ^{13}C NMR (101 MHz, $CDCl_3$) δ 172.76, 168.53, 143.61, 140.05, 132.95, 132.16, 128.96, 123.71, 110.98, 85.73, 78.72, 36.13, 26.57. [M+H]+ Calcd for $C_{13}H_{11}INO_3$ 355.9784; Found 355.9750.

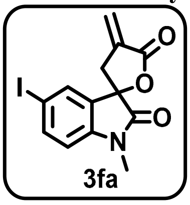

1',1'''-(propane-1,3-diyl)bis(4-methylene-3,4-dihydro-5H-spiro[furan-2,3'-indoline]-2',5-dione) (3ga):[2] White solid, 362.3 mg, yield 77%. m.p. 117–118 °C; ^{1}H NMR (400 MHz, DMSO-d_6) δ 7.62 (d, *J* = 7.4 Hz, 2H), 7.45 (t, *J* = 7.7 Hz, 2H), 7.20 – 7.14 (m, 4H), 6.20 (bs, 2H), 5.87 – 5.86 (m, 2H), 3.75 (t, *J* = 7.2 Hz, 4H), 3.31 – 3.25 (m, 4H), 1.99 – 1.94 (m, 2H). ^{13}C NMR (101 MHz, DMSO-d_6) δ 173.85, 173.82, 169.60, 143.53, 143.49, 134.15, 131.82, 126.41, 125.59, 123.83, 122.71, 110.06, 79.93, 37.82, 37.70, 35.92, 35.88, 25.31, 25.12. [M+H]+ Calcd for $C_{27}H_{23}N_2O_6$ 471.1556; Found 471.1540.

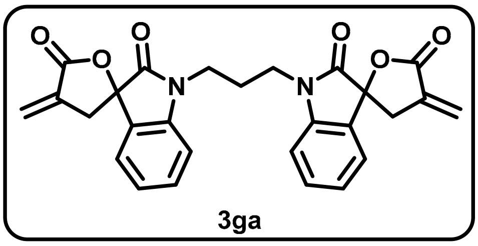

1',1'''-(heptane-1,7-diyl)bis(4-methylene-3,4-dihydro-5H-spiro[furan-2,3'-indoline]-2',5-dione) (3ha):[2]

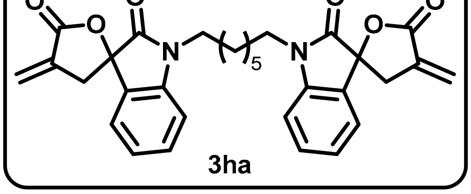

White solid, 416.0 mg, yield 79%. m.p. 119–120 °C; ^{1}H NMR (400 MHz, DMSO-d_6) δ 7.61 (d, *J* = 7.5 Hz, 2H), 7.44 (t, *J* = 7.8 Hz, 2H), 7.15 – 7.12 (m, 4H), 6.21 – 6.20 (m, 2H), 5.87 (bs, 2H), 3.64 (t, *J* = 7.1 Hz, 4H), 3.26 – 3.25 (m, 4H), 1.61 – 1.56 (m, 4H), 1.30 – 1.26 (m, 6H). ^{13}C NMR (101 MHz, DMSO-d_6) δ 173.76, 169.62, 143.72, 134.18, 131.81, 126.39, 125.50, 123.66, 122.71,

110.13, 79.92, 35.96, 28.58, 27.01, 26.43. [M+H]+ Calcd for $C_{31}H_{30}N_2NaO_6$ 549.2002; Found 549.2003.

1',1'''-(propane-1,3-diyl)bis(5'-iodo-4-methylene-3,4-dihydro-5H-spiro[furan-2,3'-indoline]-2',5-dione) (3ia): White solid, 498.4 mg, yield 69%. m.p. 125–126 °C; ^{1}H NMR (400 MHz, DMSO-d_6) δ 8.04 (d, J = 1.6 Hz, 2H), 7.80 (dd, J = 8.3, 1.5 Hz, 2H), 7.06 (d, J = 8.2 Hz, 2H), 6.18 (bs, 2H), 5.86 – 5.85 (m, 2H), 3.70 (t, J = 7.1 Hz, 4H), 3.27 – 3.18 (m, 4H), 1.94.1.88 (m, 2H). ^{13}C NMR (101 MHz, DMSO-d_6) δ 173.34, 169.40, 143.41, 140.15, 134.13, 133.95, 128.81, 122.69, 112.49, 86.80, 79.43, 37.85, 37.78, 35.74, 35.70, 25.07, 24.88. HRMS (ESI-TOF) m/z: [M+H]$^+$ Calcd for $C_{27}H_{21}I_2N_2O_6$ 722.9489; Found 722.9468.

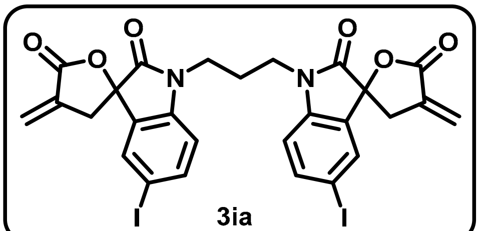
3ia

4'-chloro-7'-methyl-4-methylene-3,4-dihydro-5H-spiro[furan-2,3'-indoline]-2',5-dione (3ab): Yellow semi solid, 230.1 mg, yield 79%.; ^{1}H NMR (400 MHz, $CDCl_3$) δ 7.96 (s, 1H), 7.23 – 7.22 (m, 3H), 7.17 (td, J = 7.8, 1.2 Hz, 1H), 7.05 – 7.04 (m, 2H), 6.83 – 6.76 (m, 2H), 6.70 (d, J = 2.9 Hz, 1H), 6.64 (d, J = 7.5 Hz, 1H), 5.83 (d, J = 2.6 Hz, 1H), 4.70 (t, J = 2.7 Hz, 1H). ^{13}C NMR (101 MHz, $CDCl_3$) δ 175.61, 169.28, 140.62, 136.35, 135.22, 130.85, 129.02, 128.63, 128.26, 126.24, 125.42, 124.02, 122.78, 110.57, 84.43, 52.97. HRMS (ESI-TOF) m/z: [M+H]$^+$ Calcd for $C_{18}H_{14}NO_3$ 292.0974; Found 292.1002.

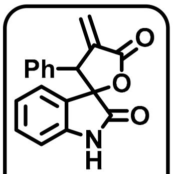
3ab

5'-chloro-4-methylene-3-phenyl-3,4-dihydro-5H-spiro[furan-2,3'-indoline]-2',5-dione (3bb): White solid, 263.8 mg, yield 81%. m.p. 188–189 °C; ^{1}H NMR (600 MHz, $CDCl_3$) δ 8.67 (s, 1H), 7.30 – 7.29 (m, 3H), 7.17 (dd, J = 8.3, 2.0 Hz, 1H), 7.05 – 7.04 (m, 2H), 6.77 (d, J = 8.3 Hz, 1H), 6.75 (d, J = 2.8 Hz, 1H), 6.54 (d, J = 1.8 Hz, 1H), 5.89 (d, J = 2.5 Hz, 1H), 4.70 (t, J = 2.5 Hz, 1H). ^{13}C NMR (151 MHz, $CDCl_3$) δ 175.53, 168.93, 139.11, 135.70, 134.94, 130.76, 128.83, 128.82, 128.55, 128.24, 126.61, 126.05, 125.57, 111.64, 52.78. HRMS (ESI-TOF) m/z: [M+H]$^+$ Calcd for $C_{18}H_{13}ClNO_3$ 326.0584; Found 326.0598.

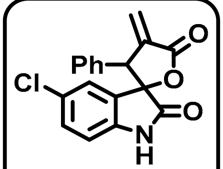
3bb

6'-bromo-4-methylene-3-phenyl-3,4-dihydro-5H-spiro[furan-2,3'-indoline]-2',5-dione (3cb): White solid, 270.2 mg, yield 73%. m.p. 180–181 °C; ^{1}H NMR (400 MHz, $CDCl_3$) δ 8.16 (s, 1H), 7.28 – 7.25 (m, 3H), 7.03 – 7.01 (m, 2H), 6.98 (d, J = 1.6 Hz, 1H), 6.93 (dd, J = 8.1, 1.7 Hz, 1H), 6.71 (d, J = 2.7 Hz, 1H), 6.41 (d, J = 8.1 Hz, 1H), 5.85 (d, J = 2.5 Hz, 1H), 4.66 (t, J = 2.6 Hz, 1H). ^{13}C NMR (101 MHz, $CDCl_3$) δ 175.29, 168.99, 141.84, 136.00, 135.29, 128.91, 128.84, 128.49, 127.57, 125.90, 125.82, 124.73, 122.84, 114.06, 83.80, 52.66. HRMS (ESI-TOF) m/z: [M+H]$^+$ Calcd for $C_{18}H_{13}BrNO_3$ 370.0079; Found 370.0093.

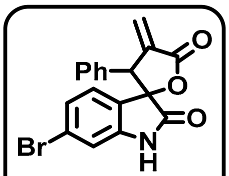
3cb

3-(3-bromophenyl)-4'-iodo-4-methylene-3,4-dihydro-5H-spiro[furan-2,3'-indoline]-2',5-dione (3db): Yellow solid, 342.3 mg, yield 69%. m.p. 258–259 °C; ^{1}H NMR (400 MHz, DMSO-d_6) δ 11.09 (s, 1H), 7.42 (bs, 1H), 7.36 (dt, J = 6.6, 2.0 Hz, 1H), 7.21 (d, J = 7.5 Hz, 1H), 7.15 – 7.12 (m, 2H), 6.88 (t, J = 7.9 Hz, 1H), 6.78 (d, J = 7.2 Hz, 1H), 6.58 (d, J = 3.6 Hz, 1H), 5.92 (d, J = 3.2 Hz, 1H), 4.85 (t, J = 3.3 Hz, 1H). ^{13}C NMR (101 MHz, DMSO-d_6) δ 175.48, 169.62, 144.44, 136.73, 135.69, 133.31, 132.81, 131.70, 131.26, 130.67, 128.19, 127.46, 125.73, 121.79, 110.74, 93.82, 85.58, 52.19. HRMS (ESI-TOF) m/z: [M+H]$^+$ Calcd for $C_{18}H_{12}BrINO_3$ 495.9045; Found 495.9074.

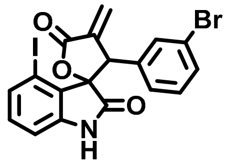
3db

4'-methylenespiro[indoline-3,2'-pyrrolidine]-2,5'-dione (5a):[3] White solid, 342.3 mg, yield 69%. m.p. 236–237 °C; ^{1}H NMR (400 MHz, DMSO-d_6) δ 10.52 (s, 1H), 8.64 (s, 1H), 7.30 – 7.25 (m, 2H), 7.02 (td, J = 7.6, 0.7 Hz, 1H), 6.87 (d, J = 7.7 Hz, 1H), 5.82 (bs, 1H), 5.40 (bs, 1H), 3.04 – 2.99 (m, J = 19.2, 2.0 Hz, 1H), 2.85 (dt, J = 17.2, 2.8 Hz, 1H). ^{13}C NMR (101 MHz, DMSO-d_6) δ 178.66, 170.00, 142.20, 139.73, 131.09, 130.04, 124.33, 122.82, 115.22, 110.43, 61.08, 37.86. [M+H]+ Calcd for $C_{12}H_{11}N_2O_2$ 215.0821; Found 215.0836.

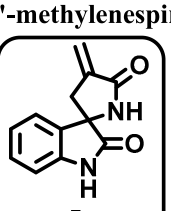

5a

5-methoxy-4'-methylenespiro[indoline-3,2'-pyrrolidine]-2,5'-dione (5b): White solid, 197.8 mg, yield 81%. m.p. 265–266 °C; ^{1}H NMR (400 MHz, DMSO-d_6) δ 10.33 (s, 1H), 8.65 (s, 1H), 6.96 (d, J = 2.4 Hz, 1H), 6.83 (dd, J = 8.4, 2.5 Hz, 1H), 6.78 (d, J = 8.4 Hz, 1H), 5.81 (bs, 1H), 5.39 (bs, 1H), 3.71 (s, 3H), 3.00 (m, 1H), 2.88 (dt, J = 17.3, 2.7 Hz, 1H). ^{13}C NMR (101 MHz, DMSO-d_6) δ 178.66, 170.03, 155.92, 139.81, 135.30, 132.16, 115.12, 115.06, 110.94, 61.53, 56.05, 37.85. HRMS (ESI-TOF) m/z: $[M+H]^+$ Calcd for $C_{13}H_{13}N_2O_3$ 245.0926; Found 245.0945

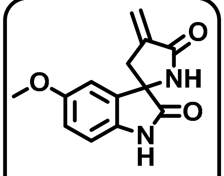

5b

4'-methylene-5-(trifluoromethoxy)spiro[indoline-3,2'-pyrrolidine]-2,5'-dione (5c): White solid, 247.5 mg, yield 83%. m.p. 210–211 °C; ^{1}H NMR (400 MHz, DMSO-d_6) δ 10.70 (s, 1H), 8.65 (s, 1H), 7.42 (s, 1H), 7.29 (d, J = 8.5 Hz, 1H), 6.95 (d, J = 8.5 Hz, 1H), 5.82 (s, 1H), 5.41 (s, 1H), 3.03 (d, J = 17.2 Hz, 1H), 2.92 (d, J = 17.2 Hz, 1H). ^{13}C NMR (101 MHz, DMSO-d_6) δ 178.75, 169.97, 144.07, 141.51, 139.39, 132.73, 123.39, 120.26 (q, J = 252.5), 118.50, 116.53, 111.33, 61.30, 37.55. HRMS (ESI-TOF) m/z: $[M+H]^+$ Calcd for $C_{13}H_{10}F_3N_2O_3$ 299.0644; Found 299.0665.

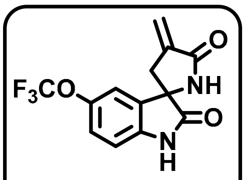

5c

5-fluoro-4’-methylenespiro[indoline-3,2’-pyrrolidine]-2,5’-dione (5d): White solid, 190.4 mg, yield 82%. m.p. 256–257 °C; ^{1}H NMR (400 MHz, DMSO-d_6) δ 10.54 (s, 1H), 8.66 (s, 1H), 7.27 (dd, J = 8.2, 2.6 Hz, 1H), 7.13 – 7.08 (m, 1H), 6.86 (dd, J = 8.5, 4.3 Hz, 1H), 5.82 (bs, 1H), 5.40 (bs, 1H), 3.04 – 2.87 (m, 1H), 2.90 (dt, J = 17.3, 2.8 Hz, 1H). ^{13}C NMR (101 MHz, DMSO-d_6) δ 178.73, 169.99, 158.85 (d, J = 238.4), 139.50, 138.39, 132.72 (d, J = 7.8), 116.34 (d, J = 23.4), 115.28, 112.40 (d, J = 24.8), 111.27 (d, J = 7.9), 61.44, 37.61. HRMS (ESI-TOF) m/z: $[M+H]^+$ Calcd for $C_{12}H_{10}FN_2O_2$ 233.0726; Found 233.0741.

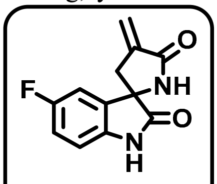

5d

5-chloro-4’-methylenespiro[indoline-3,2’-pyrrolidine]-2,5’-dione (5e): White solid, 211.3 mg, yield 85%. m.p. 275–276 °C; ^{1}H NMR (400 MHz, DMSO-d_6) δ 10.65 (s, 1H), 8.64 (s, 1H), 7.41 (d, J = 2.0 Hz, 1H), 7.32 (dd, J = 8.3, 2.1 Hz, 1H), 6.88 (d, J = 8.3 Hz, 1H), 5.82 (bs, 1H), 5.41 (bs, 1H), 3.03 – 2.99 (m, 1H), 2.92 (dt, J = 17.4, 2.7 Hz, 1H). ^{13}C NMR (101 MHz, DMSO-d_6) δ 178.45, 169.97, 141.15, 139.42, 133.04, 129.88, 126.77, 124.73, 115.37, 111.89, 61.24, 37.56. HRMS (ESI-TOF) m/z: $[M+H]^+$ Calcd for $C_{12}H_{10}ClN_2O_2$ 249.0431; Found 249.0449.

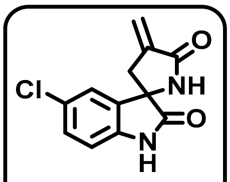

5e

5-bromo-4'-methylenespiro[indoline-3,2'-pyrrolidine]-2,5'-dione (5f): White solid, 234.5 mg, yield 80%. m.p. 283–284 °C; ^{1}H NMR (400 MHz, DMSO-d_6) δ 10.66 (s, 1H), 8.64 (s, 1H), 7.51 (s, 1H), 7.46 (d, J = 8.2 Hz, 1H), 6.83 (d, J = 8.2 Hz, 1H), 5.82 (bs, 1H), 5.40 (bs, 1H), 2.99 – 3.03 (m, 1H), 2.90- 2.94 (m, 1H). ^{13}C NMR (101 MHz, DMSO-d_6) δ 178.31, 169.95, 141.58, 139.41, 133.43, 132.73, 127.41, 115.37, 114.38, 112.40, 61.18, 37.56. HRMS (ESI-TOF) m/z: $[M+H]^+$ Calcd for $C_{12}H_{10}BrN_2O_2$ 292.9926; Found 292.9931.

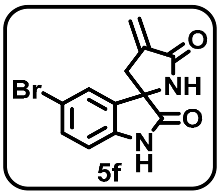

5f

5-iodo-4'-methylenespiro[indoline-3,2'-pyrrolidine]-2,5'-dione (5g): White solid, 268.7 mg, yield 79%. m.p. 270–271 °C; ^{1}H NMR (400 MHz, DMSO-d_6) δ 10.64 (s, 1H), 8.63 (s, 1H), 7.60 – 7.62 (m, 2H), 6.72 (d, J = 8.7 Hz, 1H), 5.82 (bs, 1H), 5.40 (bs, 1H), 2.98 – 3.02 (m, 1H), 2.91 (dt, J = 17.3, 2.7 Hz, 1H). ^{13}C NMR (101 MHz, DMSO-d_6) δ 178.08, 169.93, 142.03, 139.43, 138.53, 133.69, 132.78, 115.37, 112.88, 85.49, 60.98, 37.58. HRMS (ESI-TOF) m/z: $[M+H]^+$ Calcd for $C_{12}H_{10}IN_2O_2$ 340.9787; Found 340.9813.

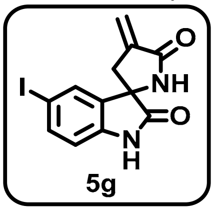

5g

4-bromo-4'-methylenespiro[indoline-3,2'-pyrrolidine]-2,5'-dione (5h): White solid, 225.7 mg, yield 77%. m.p. 287–288 °C; ^{1}H NMR (400 MHz, DMSO-d_6) δ 10.77 (s, 1H), 8.60 (s, 1H), 7.25 – 7.18 (m, 2H), 6.89 (dd, J = 7.3, 1.2 Hz, 1H), 5.84 (s, 1H), 5.42 (s, 1H), 3.06 (dt, J = 17.9, 2.7 Hz, 1H), 2.97 (dt, J = 18.0, 2.3 Hz, 1H). ^{13}C NMR (101 MHz, DMSO-d_6) δ 178.02, 169.99, 144.59, 139.64, 132.01, 128.34, 126.36, 119.19, 115.37, 109.99, 62.04, 34.42. HRMS (ESI-TOF) m/z: $[M+H]^+$ Calcd for $C_{12}H_{10}BrN_2O_2$ 292.9926; Found 292.9937.

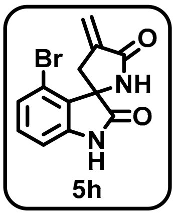

5h

5,6-difluoro-4'-methylenespiro[indoline-3,2'-pyrrolidine]-2,5'-dione (5i): White solid, 180.7 mg, yield 75%. m.p. 268–269 °C; ^{1}H NMR (400 MHz, DMSO-d_6) δ 10.65 (s, 1H), 8.63 (s, 1H), 7.56 (dd, J = 9.8, 8.0 Hz, 1H), 6.92 (dd, J = 10.5, 6.6 Hz, 1H), 5.82 (d, J = 2.1 Hz, 1H), 5.40 (s, 1H), 3.03 – 2.99 (m, 1H), 2.90 (dt, J = 17.3, 2.9 Hz, 1H). ^{13}C NMR (101 MHz, DMSO-d_6) δ 178.82, 169.92, 150.67 (dd, J = 246.6, 13.7), 146.17 (dd, J = 237.2, 13.2), 139.39, 138.94 (dd, J = 10.7, 1.9), 126.89 (dd, J = 6.1, 3.6), 115.34, 114.58 (d, J = 20.1), 100.36 (d, J = 22.7), 61.10, 37.46. HRMS (ESI-TOF) m/z: $[M+H]^+$ Calcd for $C_{12}H_9F_2N_2O_2$ 251.0632; Found 251.0655.

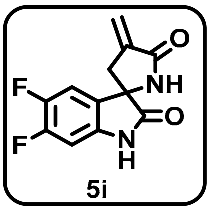

5i

5-bromo-7-methyl-4'-methylenespiro[indoline-3,2'-pyrrolidine]-2,5'-dione (5j): White solid, 239.6 mg, yield 78%. m.p. 298–299 °C; ^{1}H NMR (400 MHz, DMSO-d_6) δ 10.73 (s, 1H), 8.63 (s, 1H), 7.32 (s, 2H), 5.82 (s, 1H), 5.40 (s, 1H), 3.03 – 2.98 (m, 1H), 2.90 (dt, J = 17.2, 2.8 Hz, 1H), 2.21 (s, 3H). ^{13}C NMR (101 MHz, DMSO-d_6) δ 178.79, 169.93, 140.20, 139.45, 133.56, 132.92, 124.56, 122.46, 115.34, 114.35, 61.34, 37.69, 16.41. HRMS (ESI-TOF) m/z: [M+H]+ Calcd for $C_{13}H_{12}BrN_2O_2$ 307.0082; Found 307.0088.

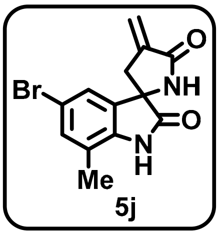

5j

4-bromo-5-methyl-4'-methylenespiro[indoline-3,2'-pyrrolidine]-2,5'-dione (5k): White solid, 224.2 mg, yield 73%. m.p. 276–277 °C; ^{1}H NMR (400 MHz, DMSO-d_6) δ 10.66 (s, 1H), 8.58 (s, 1H), 7.27 (d, J = 7.9 Hz, 1H), 6.81 (d, J = 7.9 Hz, 1H), 5.84 (s, 1H), 5.41 (s, 1H), 3.07 (dt, J = 17.9, 2.7 Hz, 1H), 3.10 – 2.94 (m, 1H), 2.29 (s, 3H). ^{13}C NMR (101 MHz, DMSO-d_6) δ 178.79, 169.93, 140.20, 139.45, 133.56, 132.92, 124.56, 122.46, 115.34, 114.35, 61.34, 37.69, 16.41. HRMS (ESI-TOF) m/z: [M+H]+ Calcd for $C_{13}H_{12}BrN_2O_2$ 307.0082; Found 307.0081

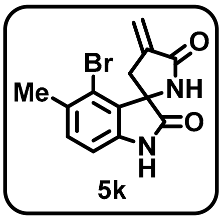

1-methyl-4'-methylenespiro[indoline-3,2'-pyrrolidine]-2,5'-dione (5l):[3] White solid, 196.3 mg, yield 86%. m.p. 174–175 °C; ^{1}H NMR (400 MHz, DMSO-d_6) δ 8.60 (s, 1H), 7.40 – 7.35 (m, 2H), 7.13 – 7.06 (m, 2H), 5.84 (t, J = 2.1 Hz, 1H), 5.42 (bs, 1H), 3.14 (s, 3H), 3.02 (dt, J = 17.3, 2.0 Hz, 1H), 2.88 (dt, J = 17.2, 2.8 Hz, 1H). ^{13}C NMR (101 MHz, DMSO-d_6) δ 176.82, 169.96, 143.71, 139.58, 130.46, 130.16, 123.94, 123.47, 115.40, 109.38, 60.80, 37.81, 26.75. [M+H]+ Calcd for $C_{13}H_{13}N_2O_2$ 229.0977.1556; Found 229.0978.

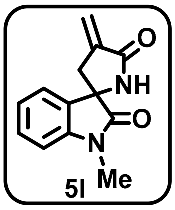

5-(furan-3-yl)-4'-methylenespiro[indoline-3,2'-pyrrolidine]-2,5'-dione (6): White solid, 232.7 mg, yield 83%. m.p. 130–131 °C; ^{1}H NMR (400 MHz, DMSO-d_6) δ 10.56 (s, 1H), 8.68 (s, 1H), 8.14 (s, 1H), 7.70 (s, 1H), 7.11 (s, 2H), 7.52 (d, J = 7.2 Hz, 1H), 6.94 (s, 1H), 6.87 (d, J = 8.0 Hz, 1H), 5.83 (s, 1H), 5.41 (s, 1H), 3.06 – 2.99 (m, 1H), 2.94 – 2.90 (m, 1H). ^{13}C NMR (101 MHz, DMSO-d_6) δ 178.77, 170.05, 144.61, 141.06, 139.80, 139.06, 131.71, 127.13, 126.97, 126.11, 121.89, 115.16, 110.64, 109.06, 61.17, 37.79. HRMS (ESI-TOF) m/z: [M+H]+ Calcd for $C_{16}H_{13}N_2O_3$ 281.0926; Found 281.0931.

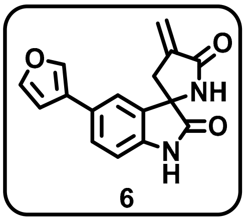

4'-methylene-5-(thiophen-3-yl)spiro[indoline-3,2'-pyrrolidine]-2,5'-dione (7): Yellow solid, 251.9 mg, yield 85%. m.p. 136–137 °C; ^{1}H NMR (400 MHz, DMSO-d_6) δ 10.58 (s, 1H), 8.68 (s, 1H), 7.80 (dd, J = 2.9, 1.3 Hz, 1H), 7.71 (d, J = 1.6 Hz, 1H), 7.63 (dd, J = 8.1, 1.8 Hz, 1H), 7.60 (dd, J = 5.0, 2.9 Hz, 1H), 7.54 (dd, J = 5.0, 1.3 Hz, 1H), 6.90 (d, J = 8.1 Hz, 1H), 5.84 (bs, 1H), 5.41 (s, 1H), 3.07 – 3.02 (m, 1H), 2.96 (dt, J = 17.3, 2.8 Hz, 1H). ^{13}C NMR (101 MHz, DMSO-d_6) δ 178.84, 170.07, 141.66, 141.28, 139.86, 131.71, 130.40, 127.76, 127.40, 126.50, 122.42, 120.16, 115.11, 110.66, 61.22, 37.79. HRMS (ESI-TOF) m/z: [M+H]+ Calcd for $C_{16}H_{13}N_2O_2S$ 297.0698; Found 297.0707.

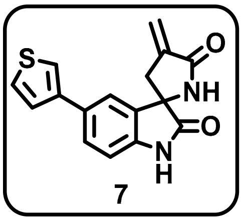

1-methyl-4'-methylene-1'-phenylspiro[indoline-3,2'-pyrrolidine]-2,5'-dione (8): White solid, 191.7 mg, yield 63%. m.p. 153-154 °C; ^{1}H NMR (600 MHz, $CDCl_3$) δ 7.37 – 7.35 (m, 1H), 7.34 (td, J = 7.8, 1.2 Hz, 1H), 7.23 – 7.20 (m, 2H), 7.19 – 7.16 (m, 1H), 7.13 (td, J = 7.6, 0.8 Hz, 1H), 7.02 – 7.00 (m, 2H), 6.80 (d, J = 7.8 Hz, 1H), 6.33 (t, J = 2.7 Hz, 1H), 5.60 (t, J = 2.3 Hz, 1H), 3.34 (dt, J = 16.7, 2.6 Hz, 1H), 3.15 (s, 3H), 3.03 (dt, J = 16.7, 2.4 Hz, 1H). ^{13}C NMR (101 MHz, $CDCl_3$) δ 175.18, 168.21, 143.04, 136.80, 136.22, 130.19, 129.00, 128.87, 127.66, 126.76, 123.63, 123.58, 117.91, 108.88, 67.52, 37.29, 26.54. HRMS (ESI-TOF) m/z: [M+H]+ Calcd for $C_{19}H_{17}N_2O_2$ 305.1290; Found 341.1317.

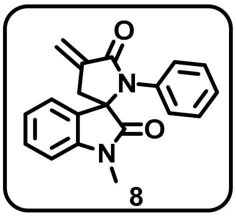

4. References

1) Yang, Z.; Huang, D.; Wen, L.; Wang, J.; Wang, K.; Hu, Y., Tin Powder-Promoted "One-Pot" Synthesis of α -Methylene- γ -butyrolactones. *Chin. J. Org. Chem.* **2018,** 38, 1725-1732.
2) Rana, S.; Kour, S.; Kizhake, S.; King, H. M.; Mallareddy, J. R.; Case, A. J.; Huxford, T.; Natarajan, A., Dimers of isatin derived α-methylene-γ-butyrolactone as potent anti-cancer agents. *Bioorg. Med. Chem. Lett.* **2022,** 65, 128713-1218719.
3) Sengoku, T.; Hayashi, D.; Takahashi, M.; Yoda, H., Electrophilic Amide Allylation of 3-Heterosubstituted Oxindoles: A Route to Spirocyclic 2-Oxindoles Containing the α-Methylene-γ-butyrolactam Structure. *European Journal of Organic Chemistry* **2018,** 2018, (15), 1813-1820

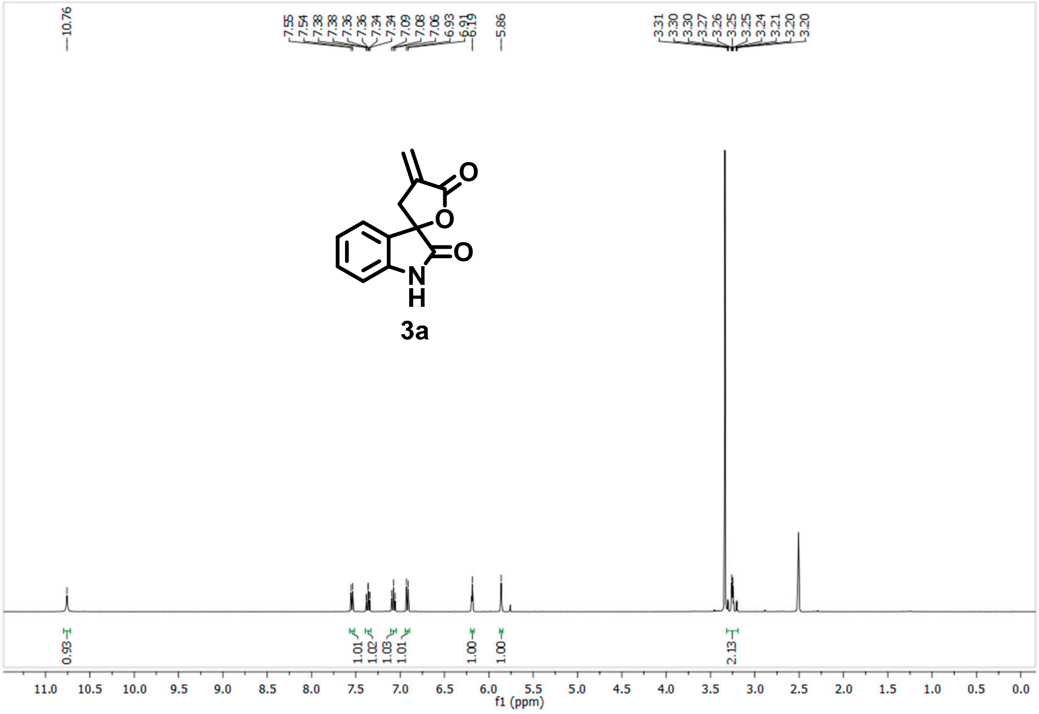

^{1}H NMR of compound **3a** at 400 MHz (DMSO-d_6)

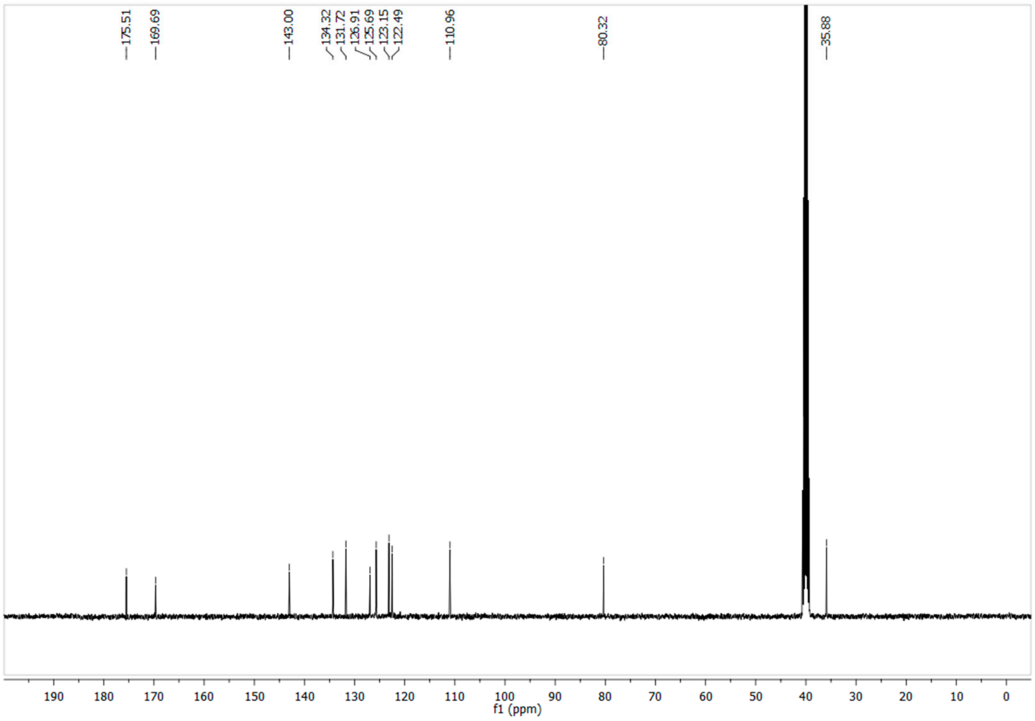

^{13}C NMR of compound **3a** at 101 MHz (DMSO-d_6)

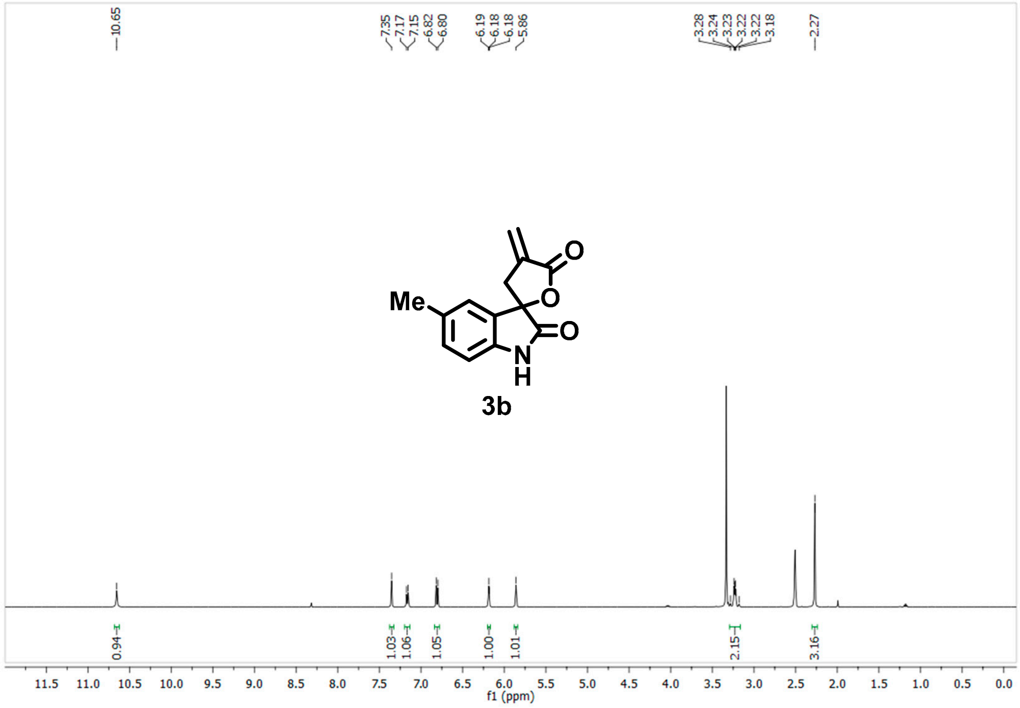

^1H NMR of compound **3b** at 400 MHz (DMSO-d_6)

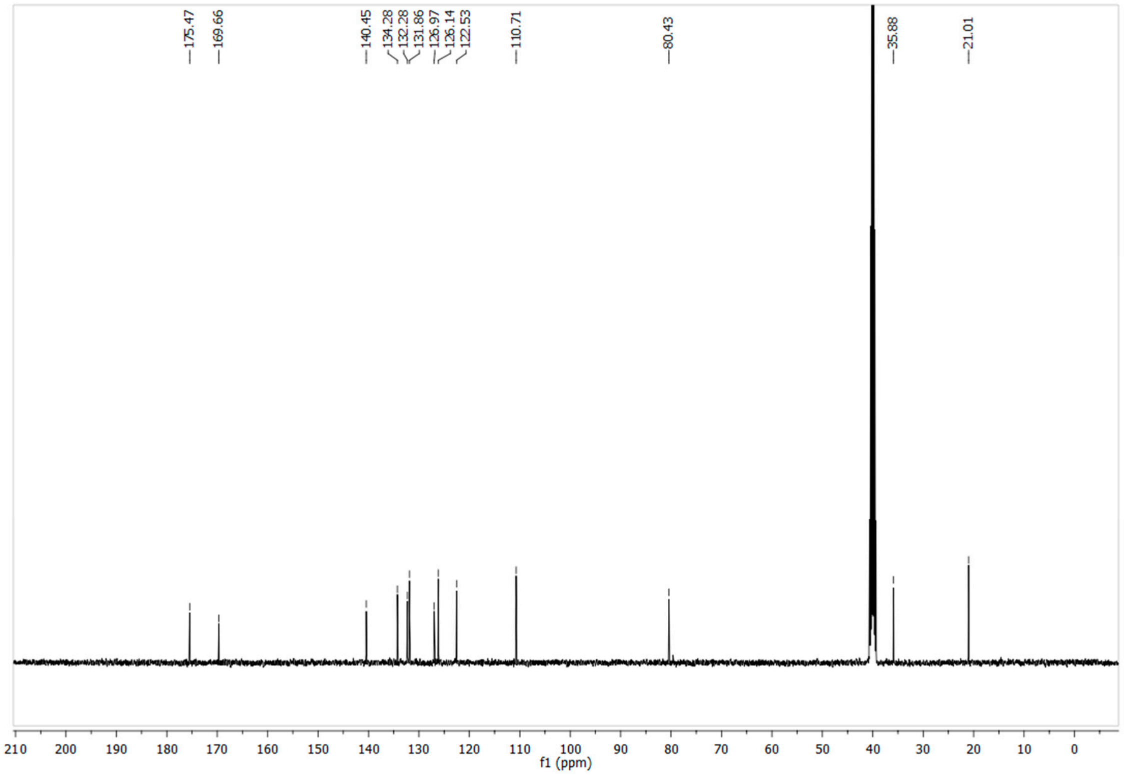

^{13}C NMR of compound **3b** at 101 MHz (DMSO-d_6)

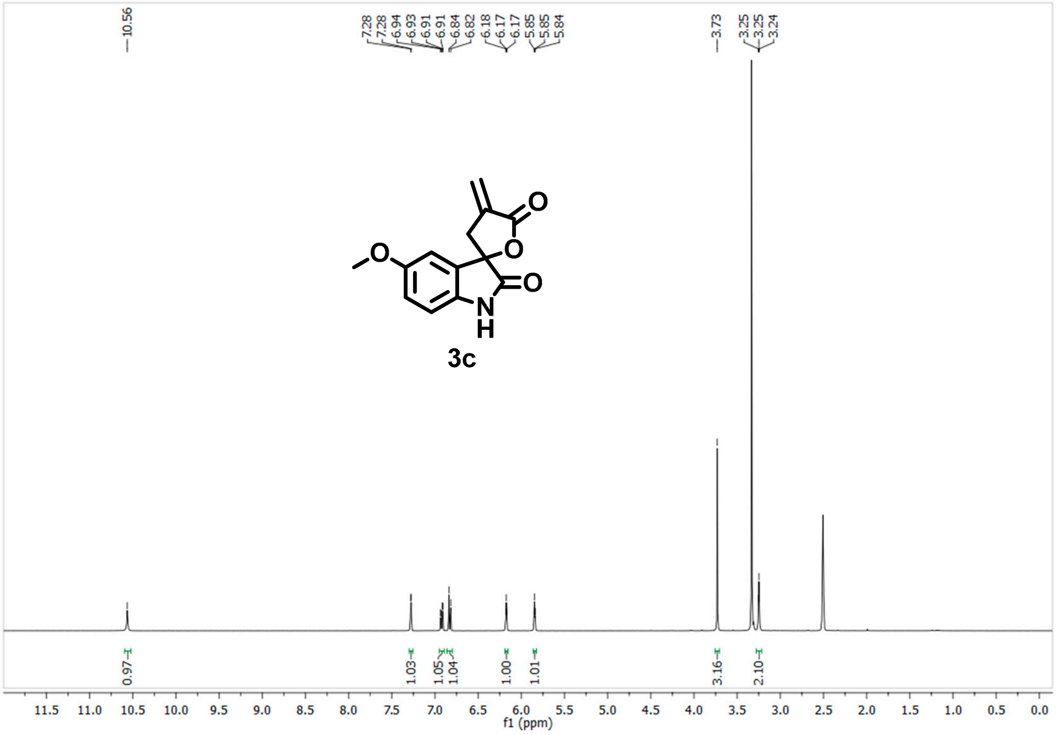

^{1}H NMR of compound **3c** at 400 MHz (DMSO-d_6)

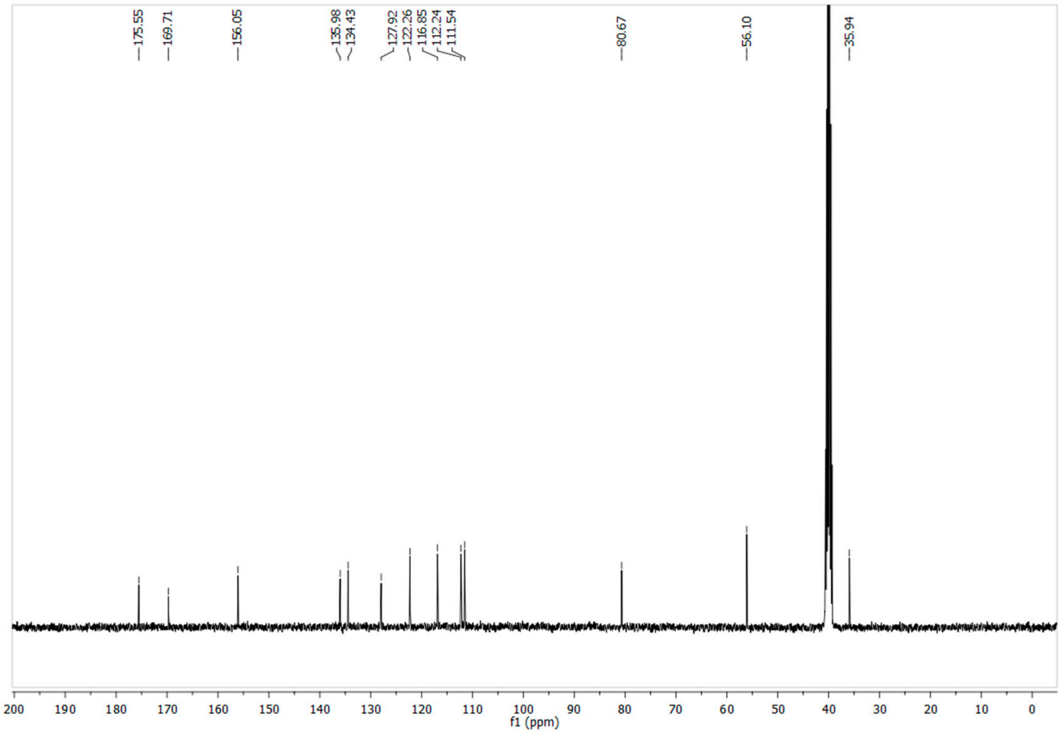

^{13}C NMR of compound **3c** at 101 MHz (DMSO-d_6)

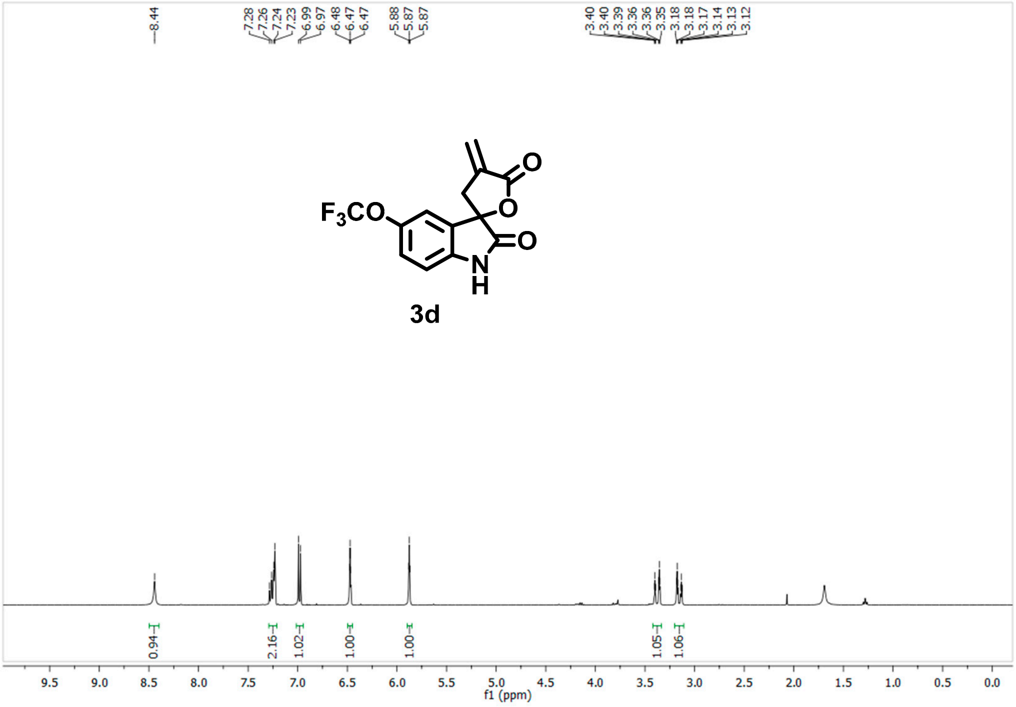

^{1}H NMR of compound **3d** at 400 MHz ($CDCl_3$)

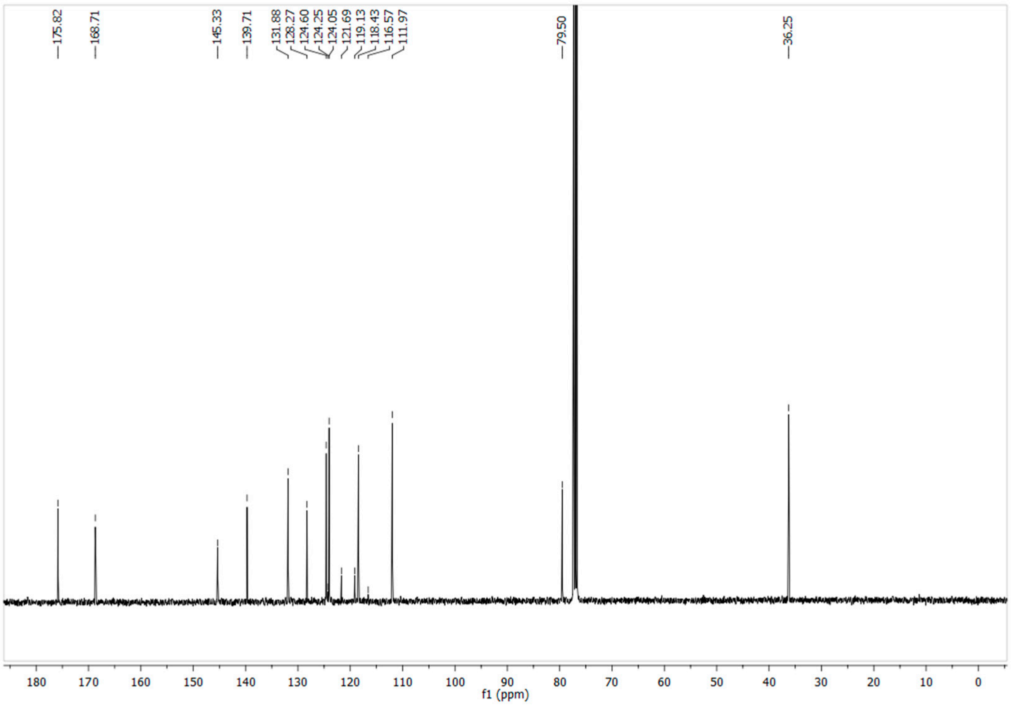

^{13}C NMR of compound **3d** at 101 MHz ($CDCl_3$)

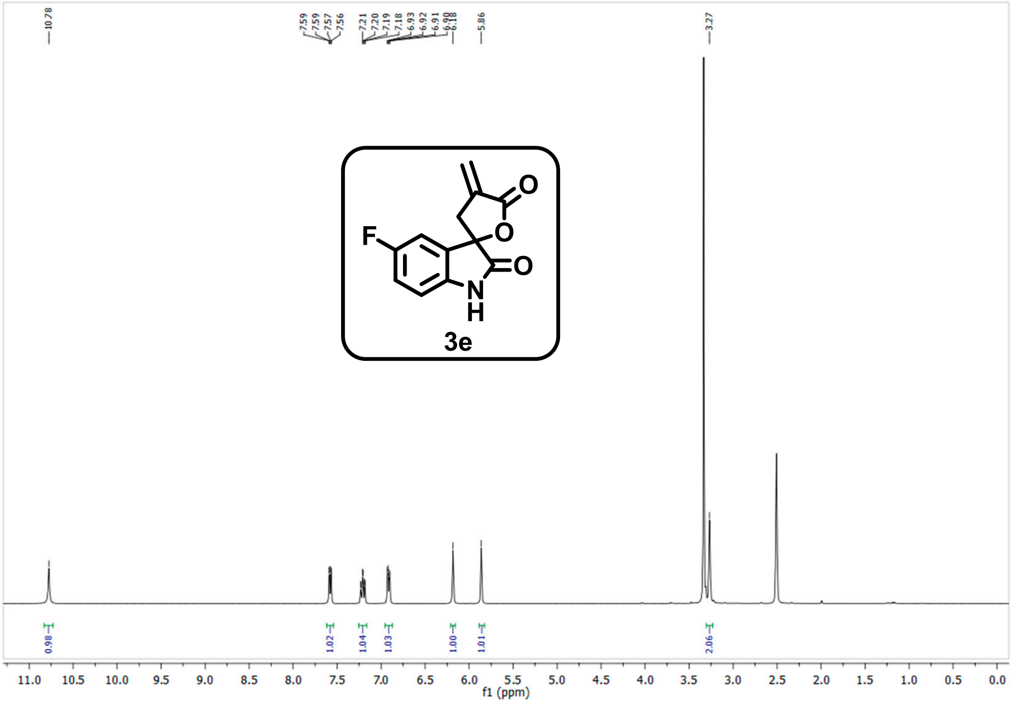

^{1}H NMR of compound **3e** at 400 MHz (DMSO-d_6)

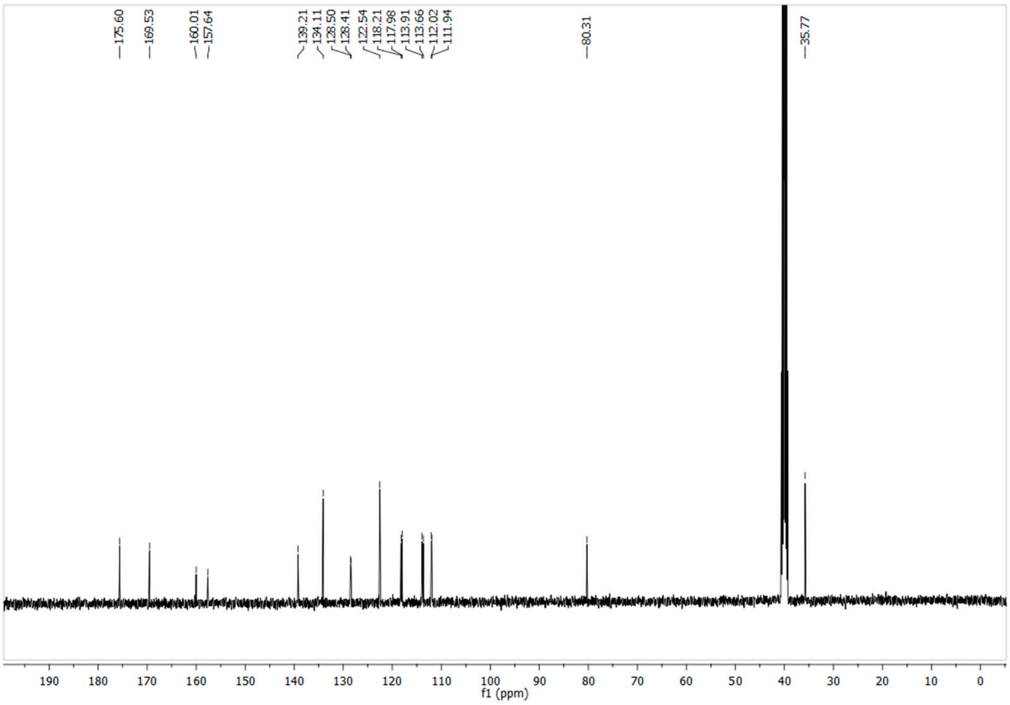

^{13}C NMR of compound **3e** at 101 MHz (DMSO-d_6)

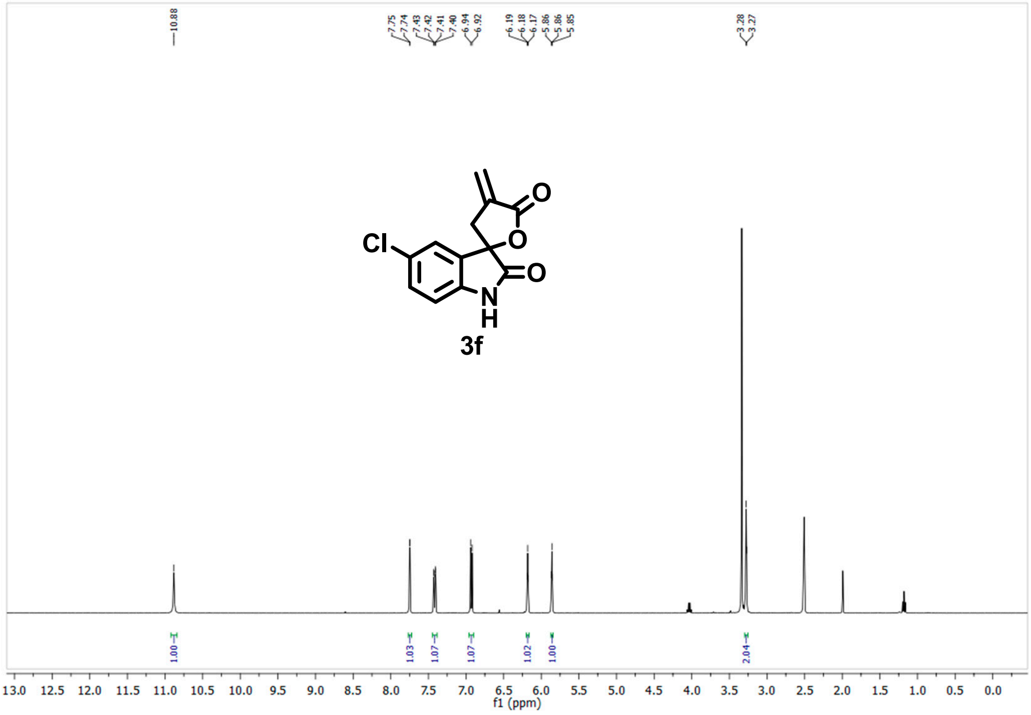

^{1}H NMR of compound **3f** at 400 MHz (DMSO-d_6)

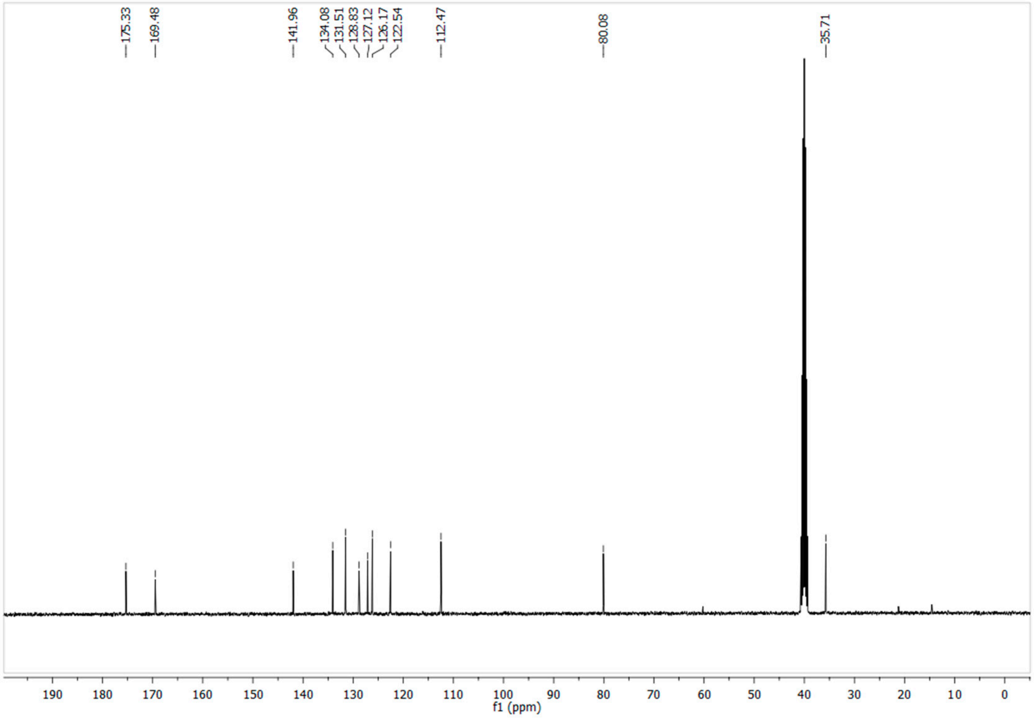

^{13}C NMR of compound **3f** at 101 MHz (DMSO-d_6)

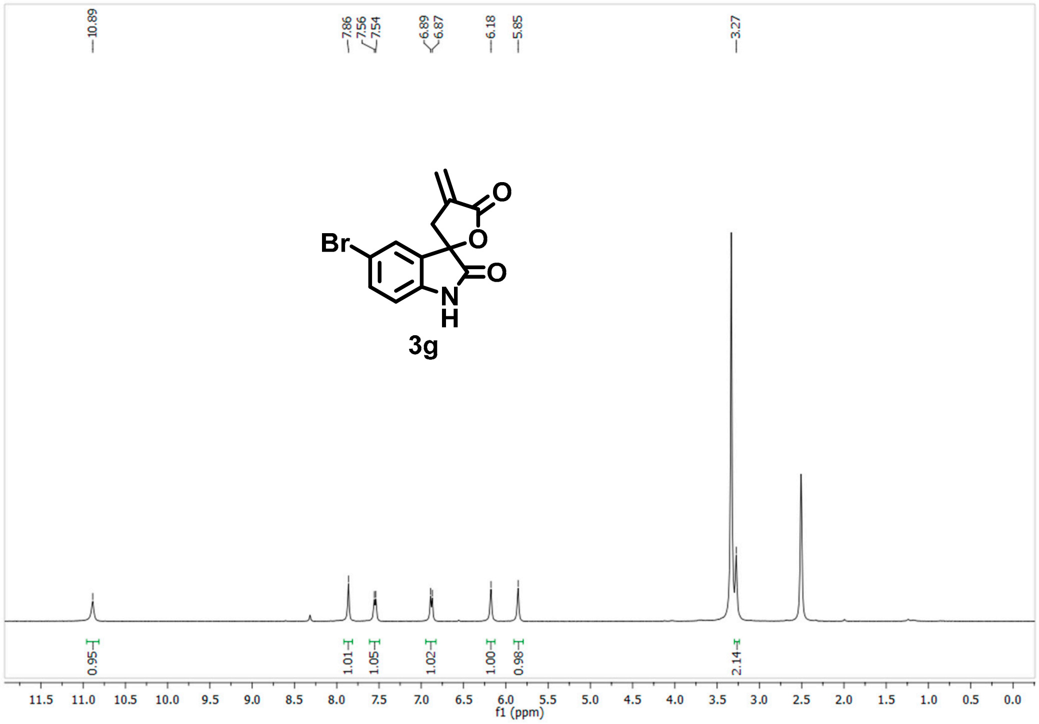

^{1}H NMR of compound **3g** at 400 MHz (DMSO-d_6)

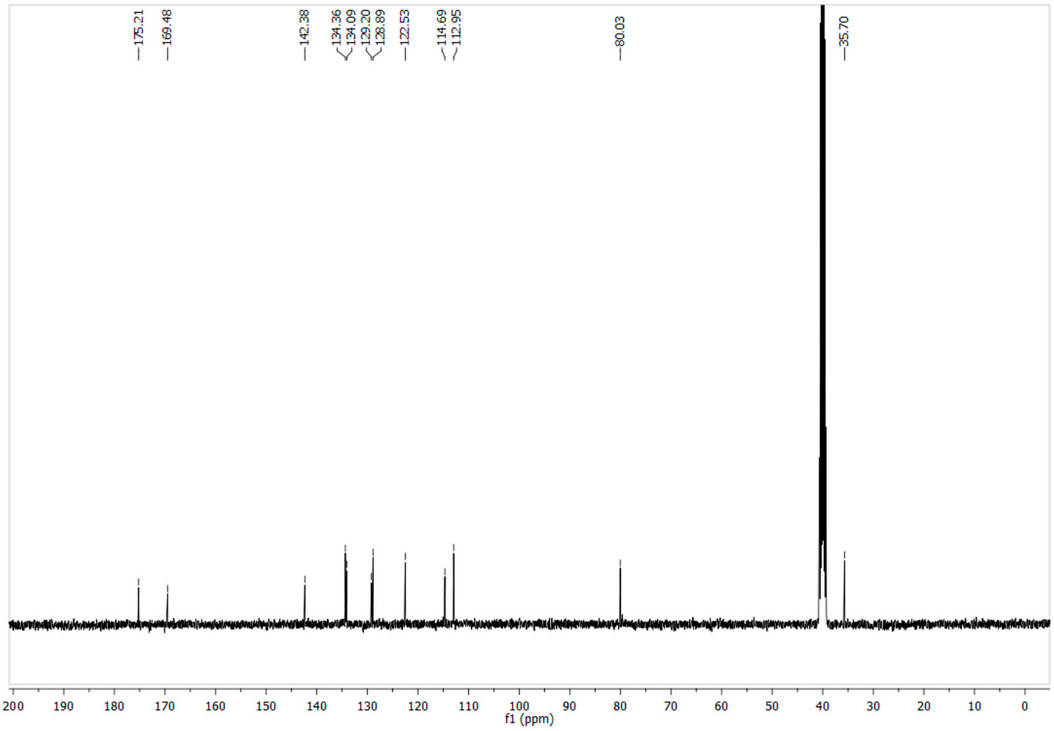

^{13}C NMR of compound **3g** at 101 MHz (DMSO-d_6)

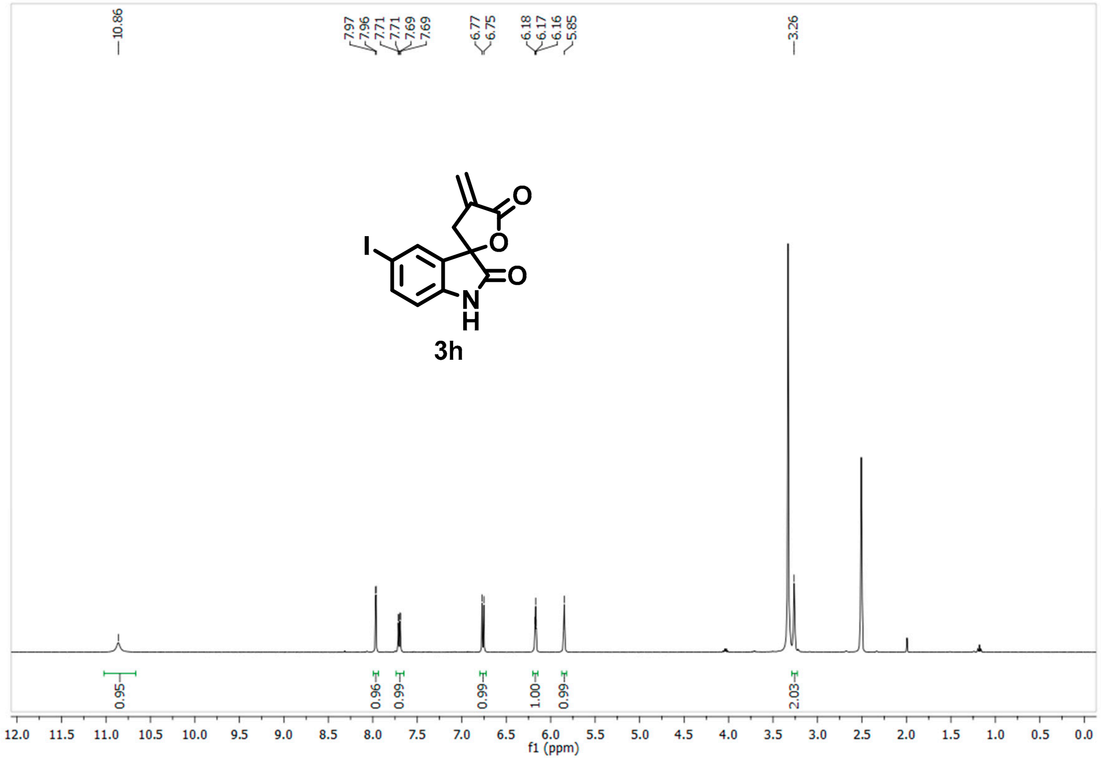

^{1}H NMR of compound **3h** at 400 MHz (DMSO-d_6)

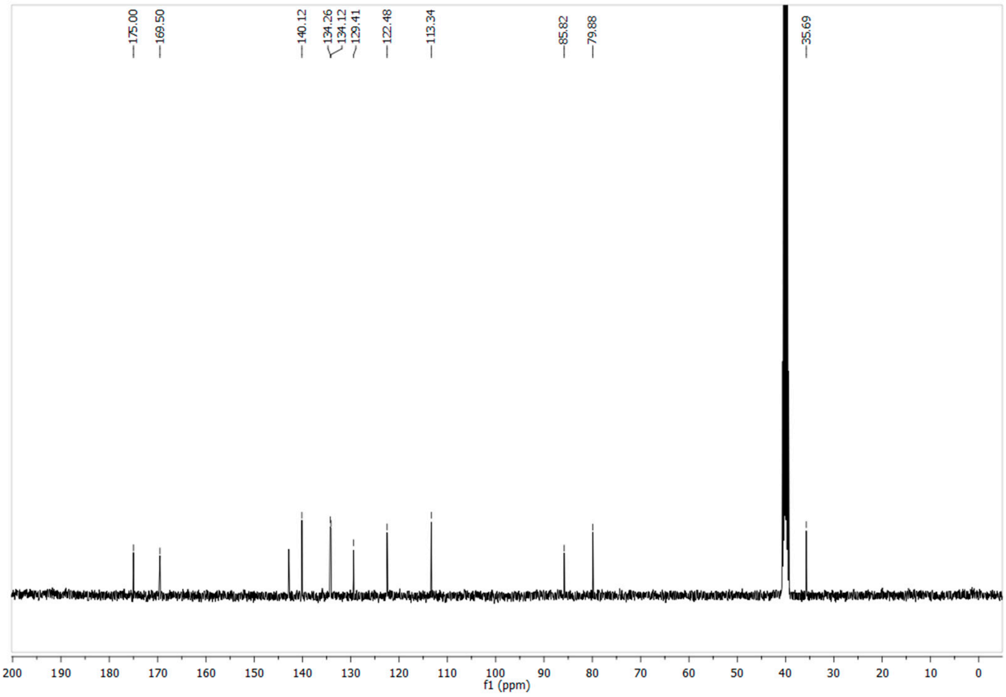

^{13}C NMR of compound **3h** at 101 MHz (DMSO-d_6)

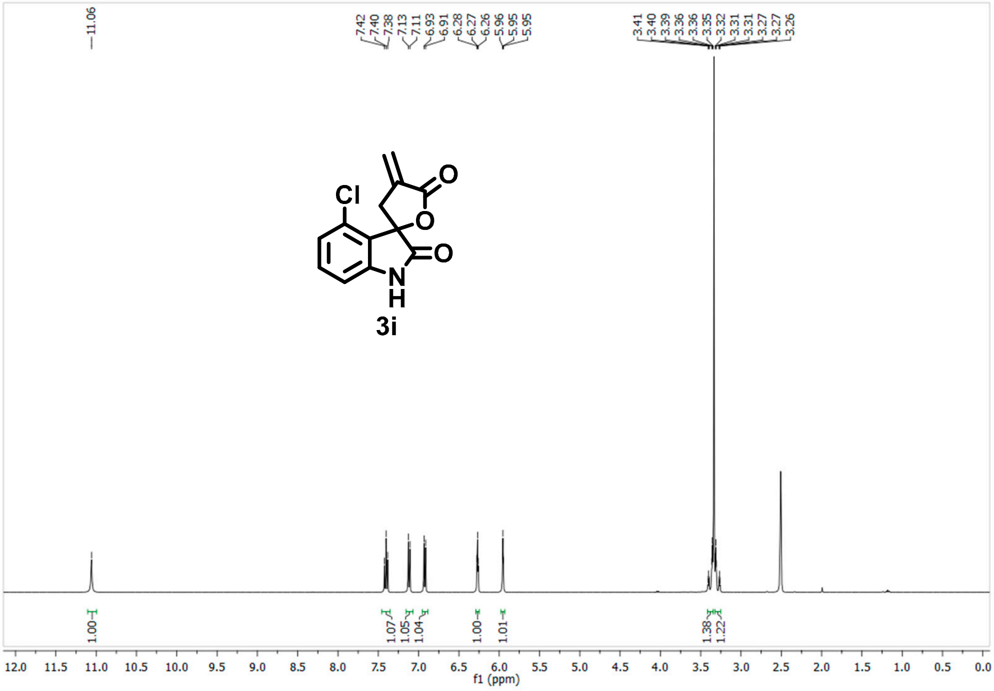

^{1}H NMR of compound **3i** at 400 MHz (DMSO-d_6)

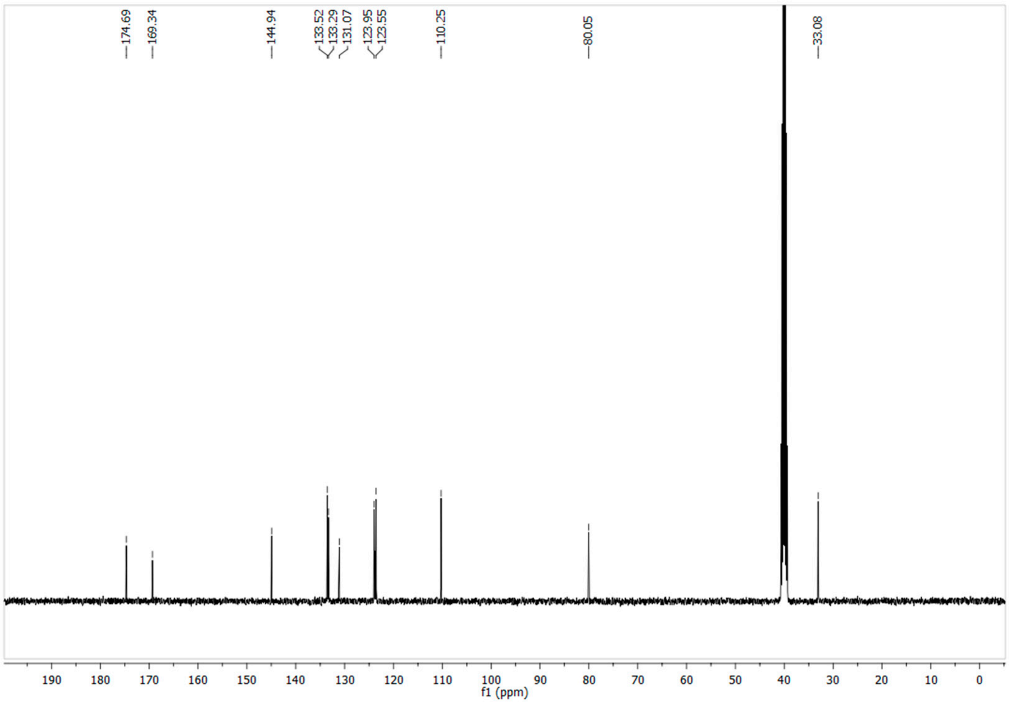

^{13}C NMR of compound **3i** at 101 MHz (DMSO-d_6)

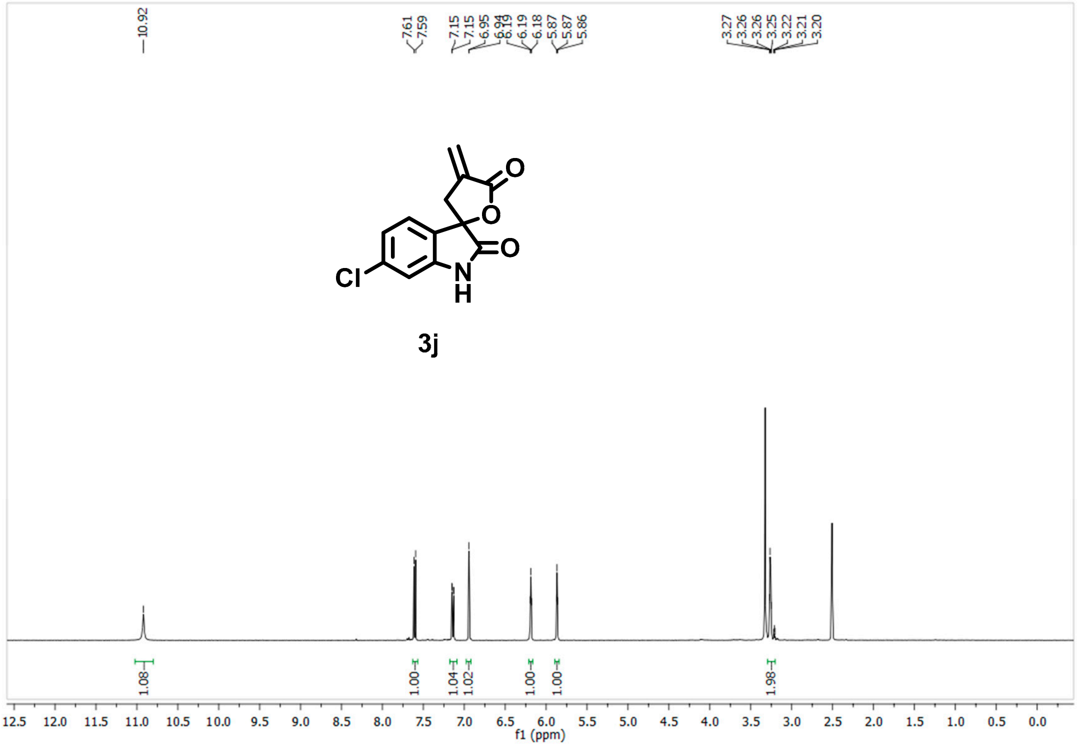

^{1}H NMR of compound **3j** at 400 MHz (DMSO-d_6)

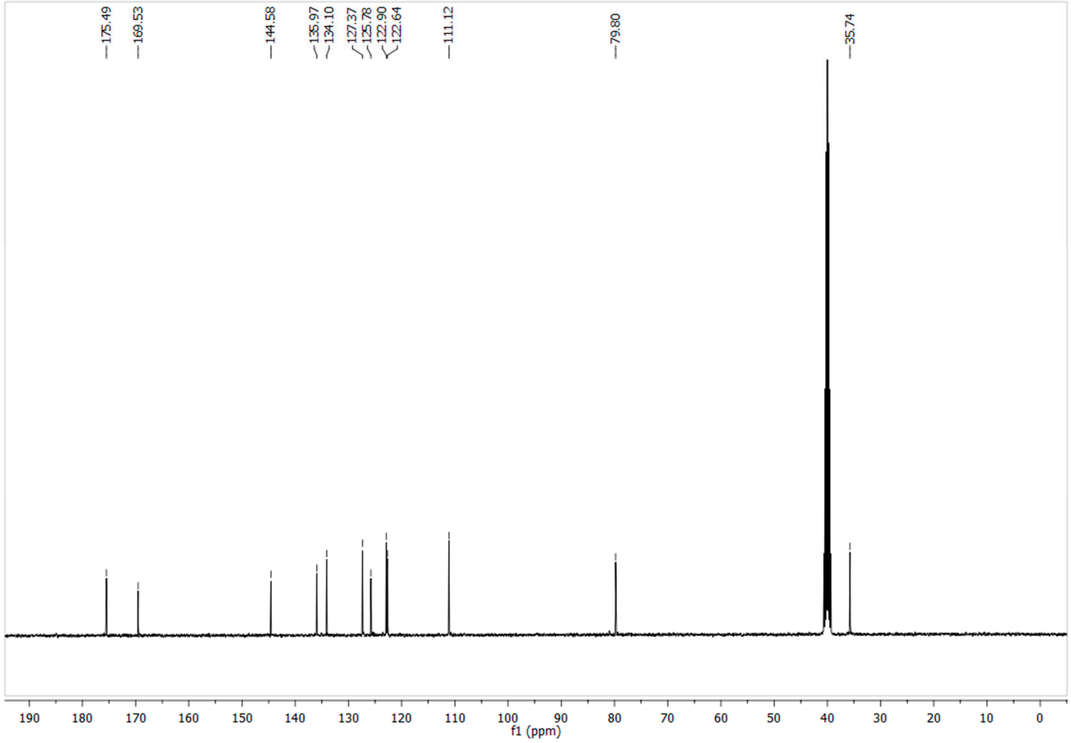

^{13}C NMR of compound **3j** at 101 MHz (DMSO-d_6)

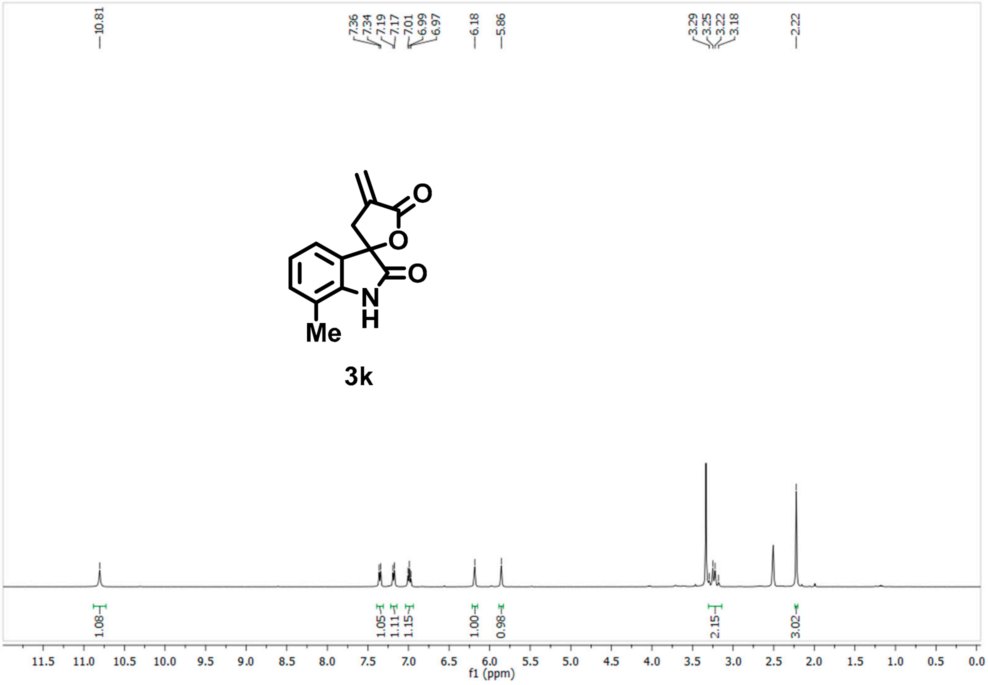

^{1}H NMR of compound **3k** at 400 MHz (DMSO-d_6)

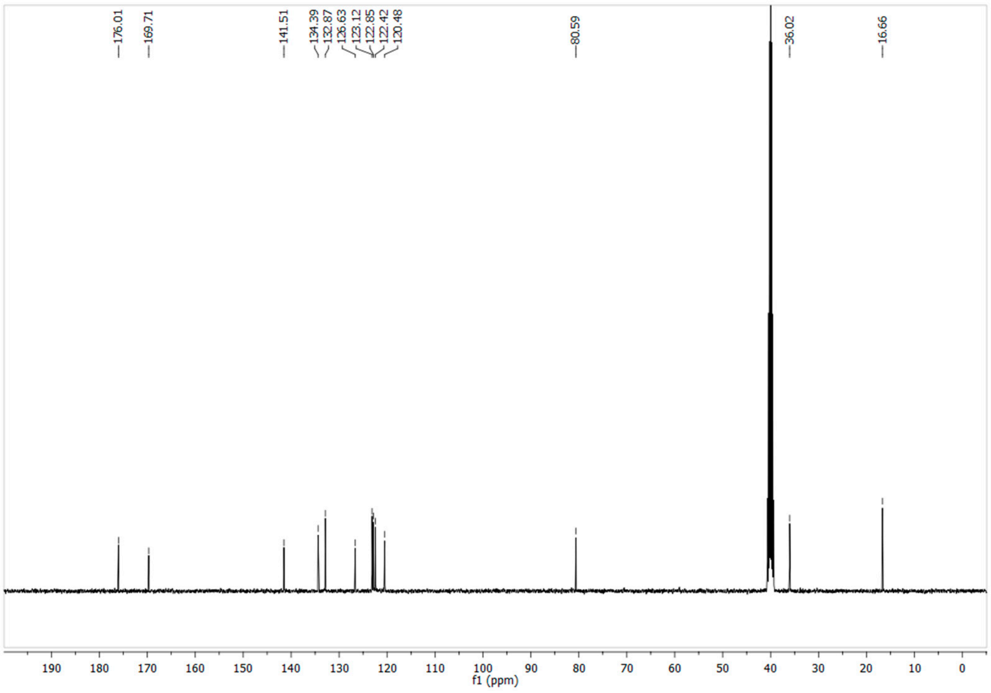

^{13}C NMR of compound **3k** at 101 MHz (DMSO-d_6)

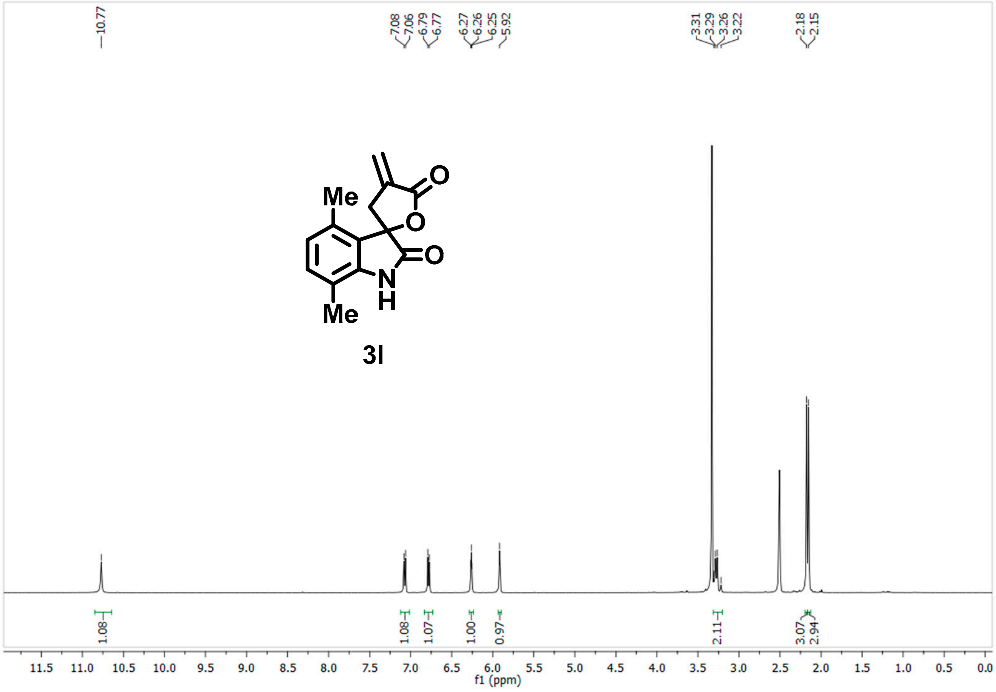

^{1}H NMR of compound **3l** at 400 MHz (DMSO-d_6)

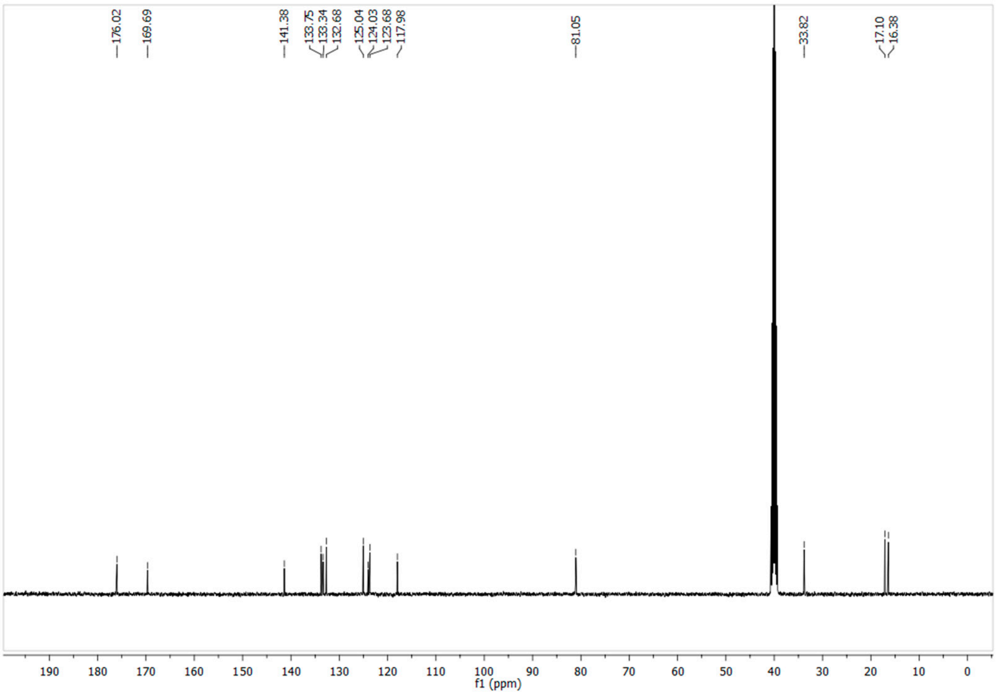

^{13}C NMR of compound **3j** at 101 MHz (DMSO-d_6)

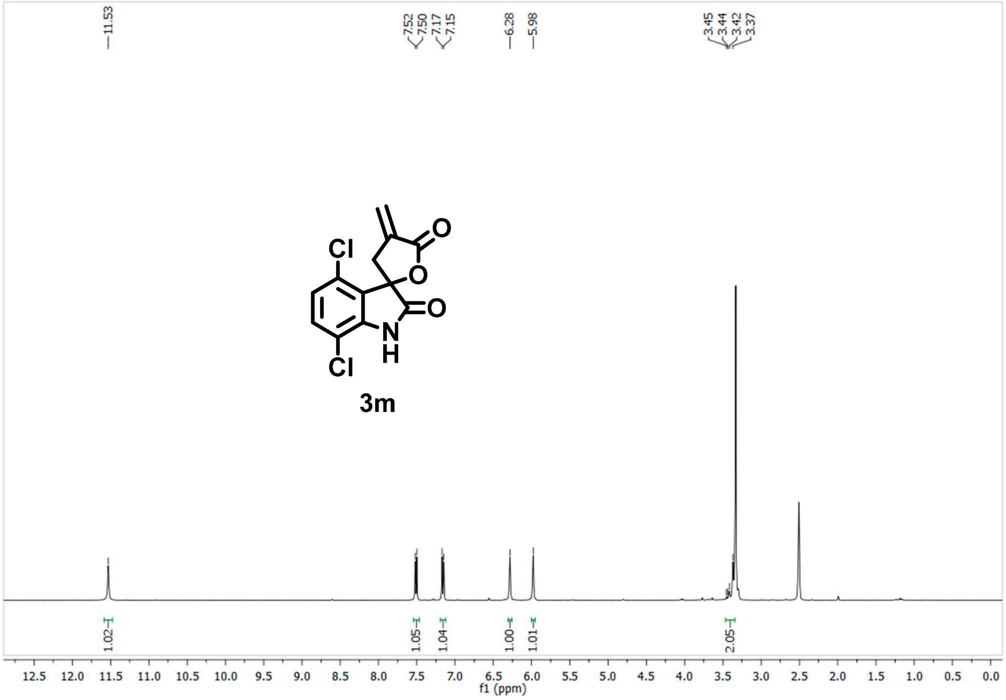

^{1}H NMR of compound **3m** at 400 MHz (DMSO-d_6)

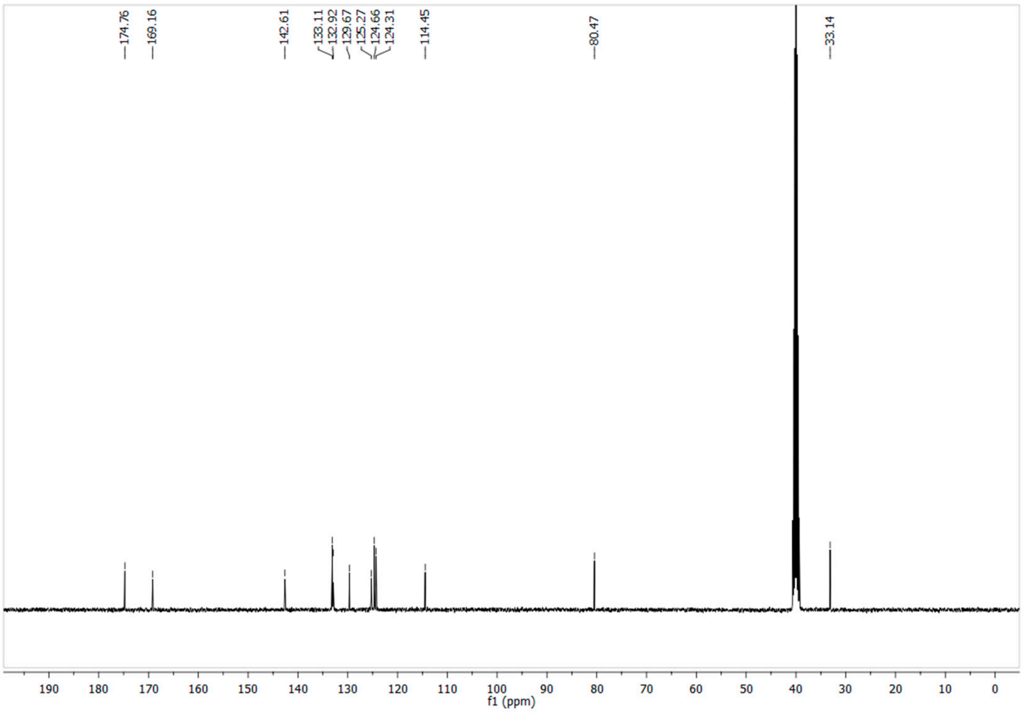

^{13}C NMR of compound **3m** at 101 MHz (DMSO-d_6)

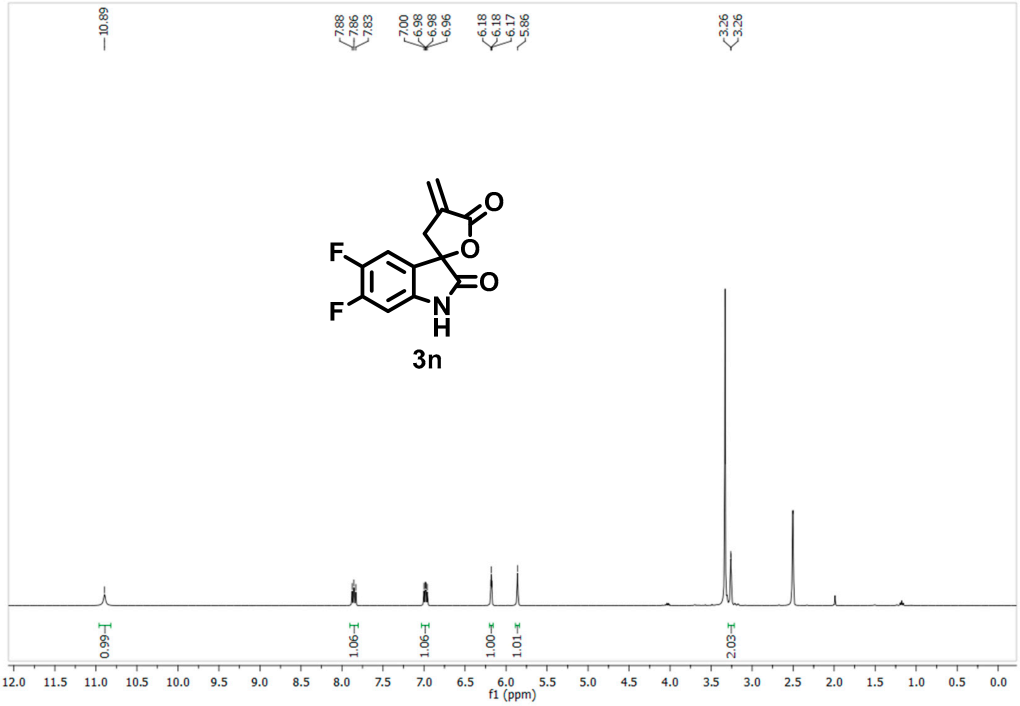

^{1}H NMR of compound **3n** at 400 MHz (DMSO-d_6)

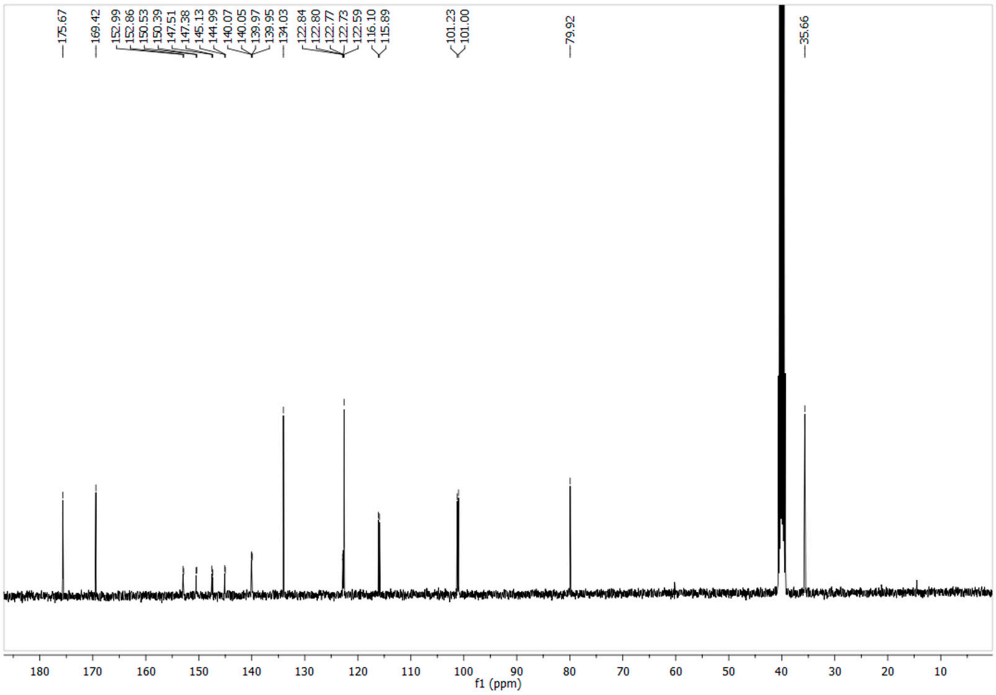

^{13}C NMR of compound **3n** at 101 MHz (DMSO-d_6)

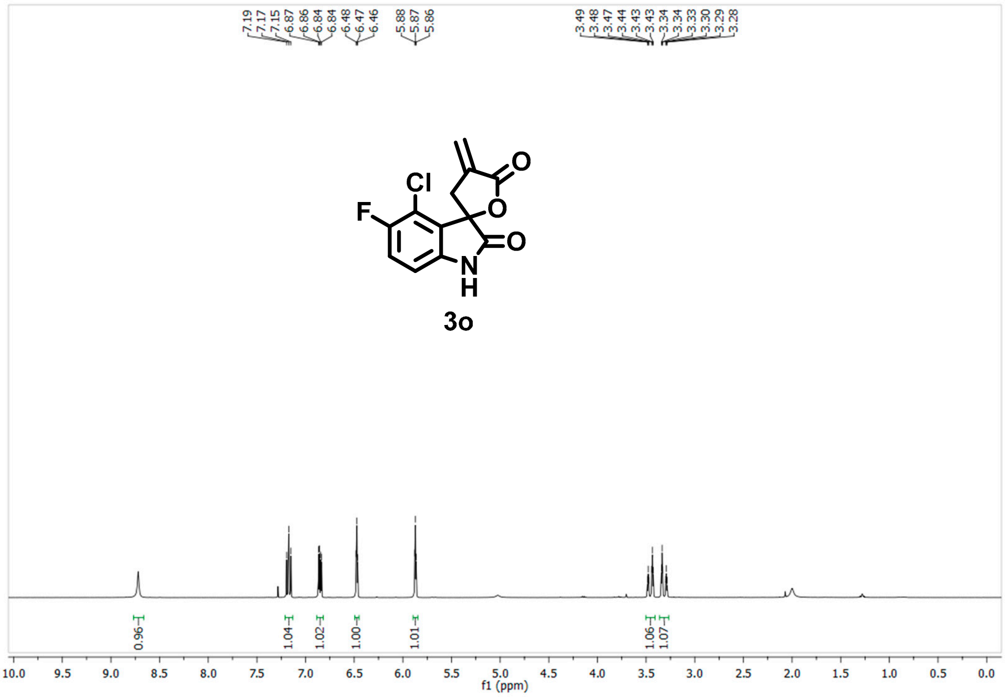

^{1}H NMR of compound **3o** at 400 MHz ($CDCl_3$)

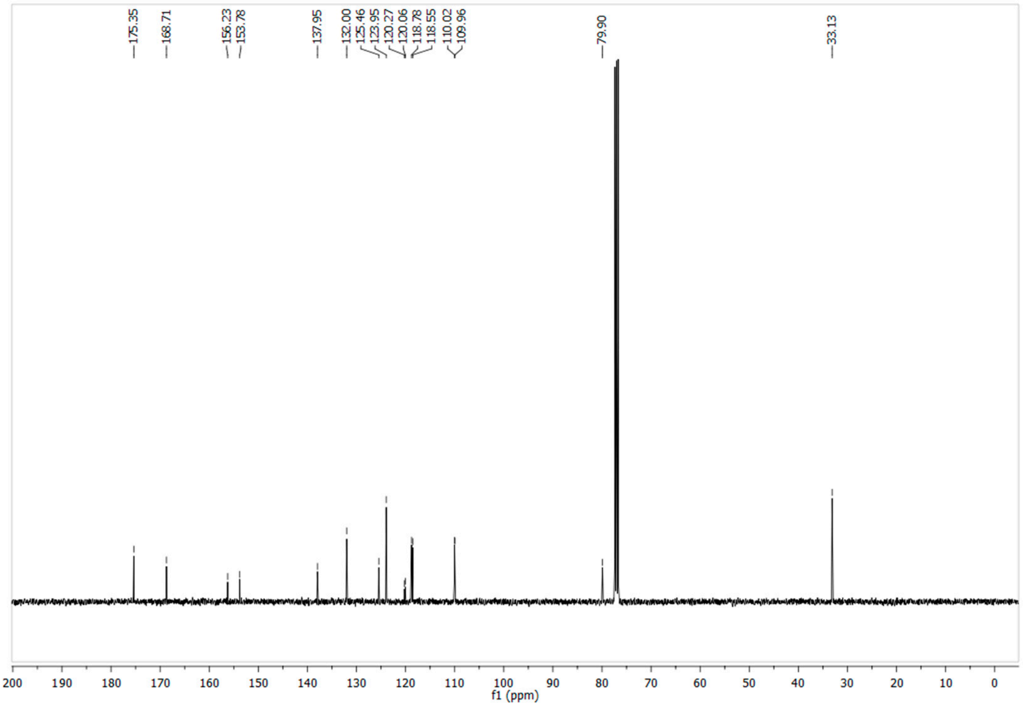

^{13}C NMR of compound **3o** at 101 MHz ($CDCl_3$)

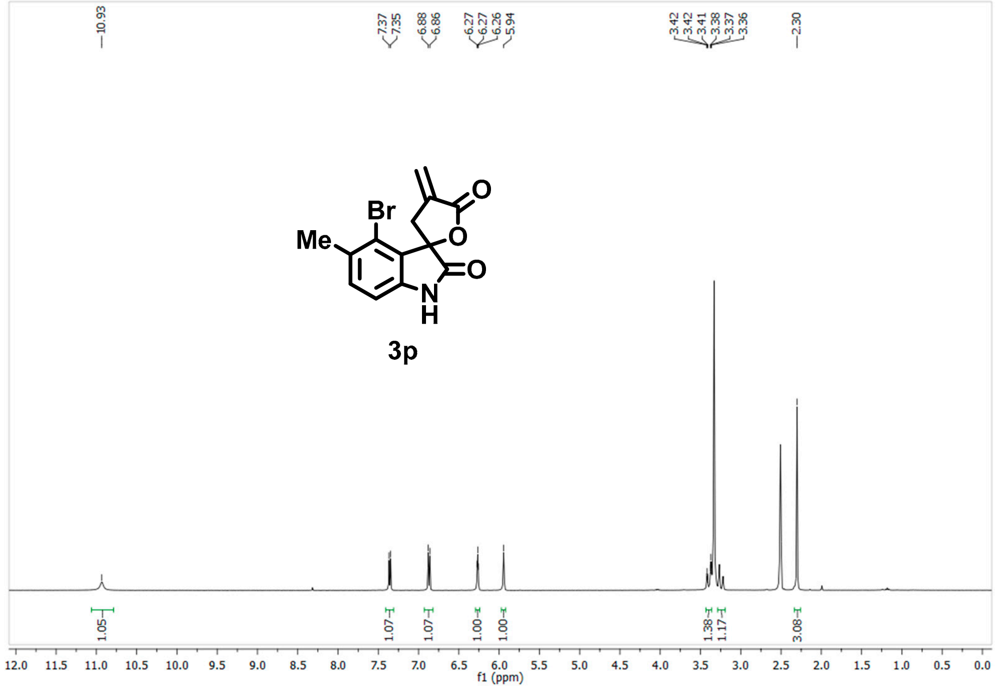

^{1}H NMR of compound **3p** at 400 MHz (DMSO-d_6)

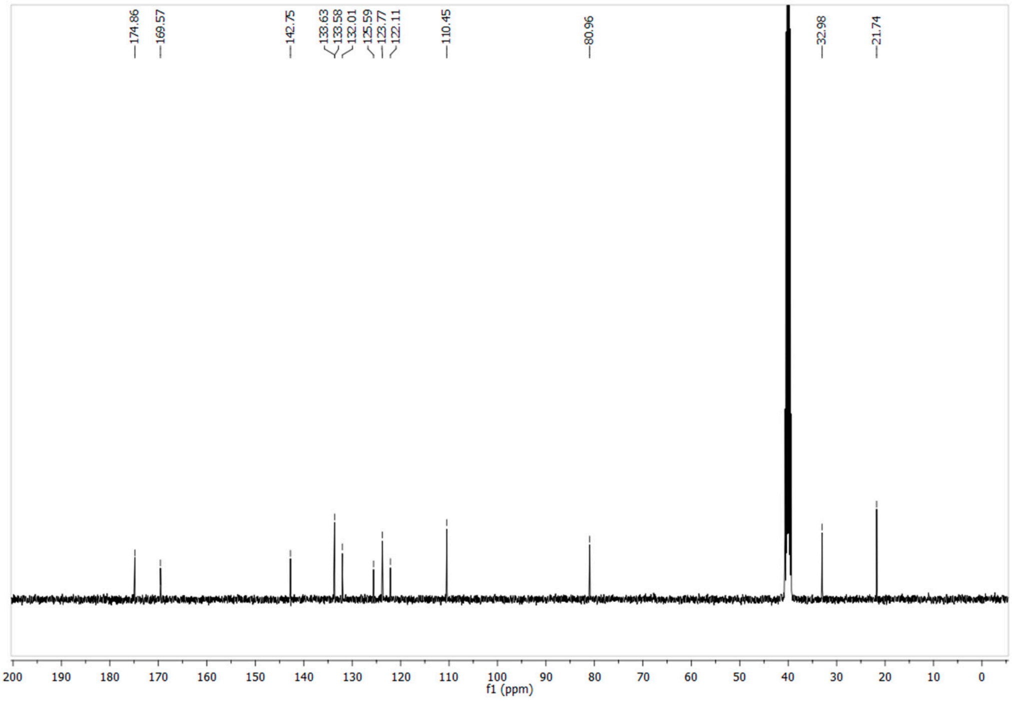

^{13}C NMR of compound **3p** at 101 MHz (DMSO-d_6)

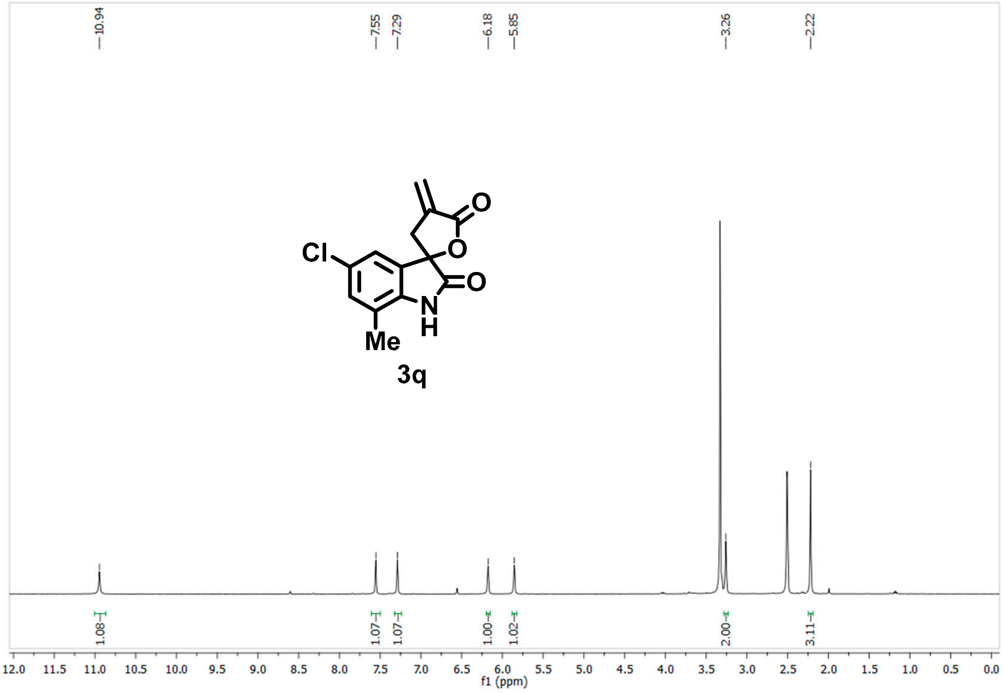

^{1}H NMR of compound **3q** at 400 MHz (DMSO-d_6)

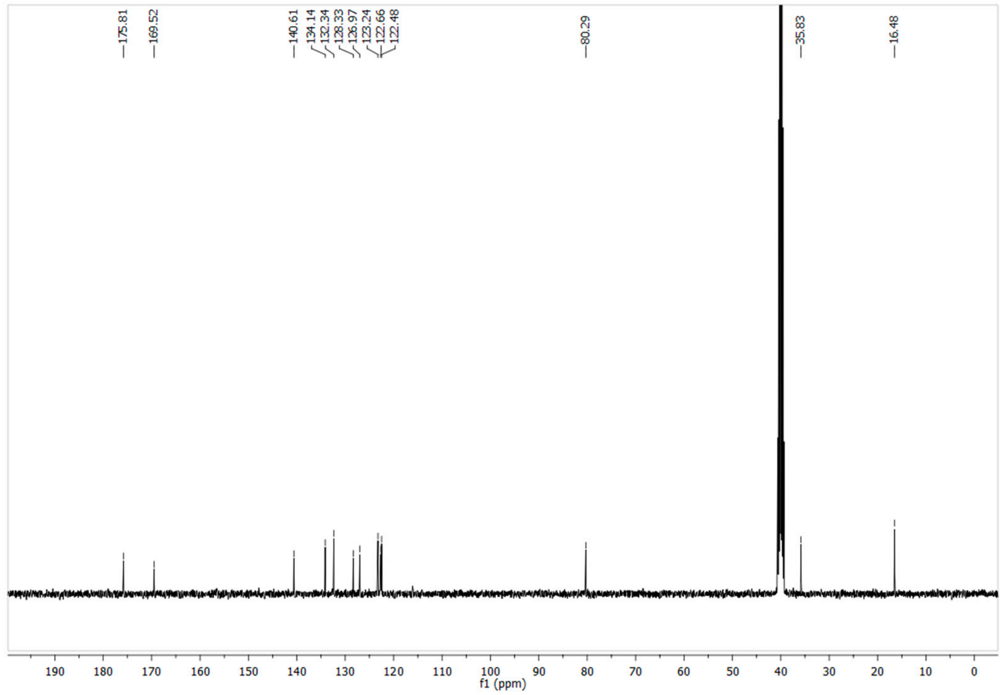

^{13}C NMR of compound **3q** at 101 MHz (DMSO-d_6)

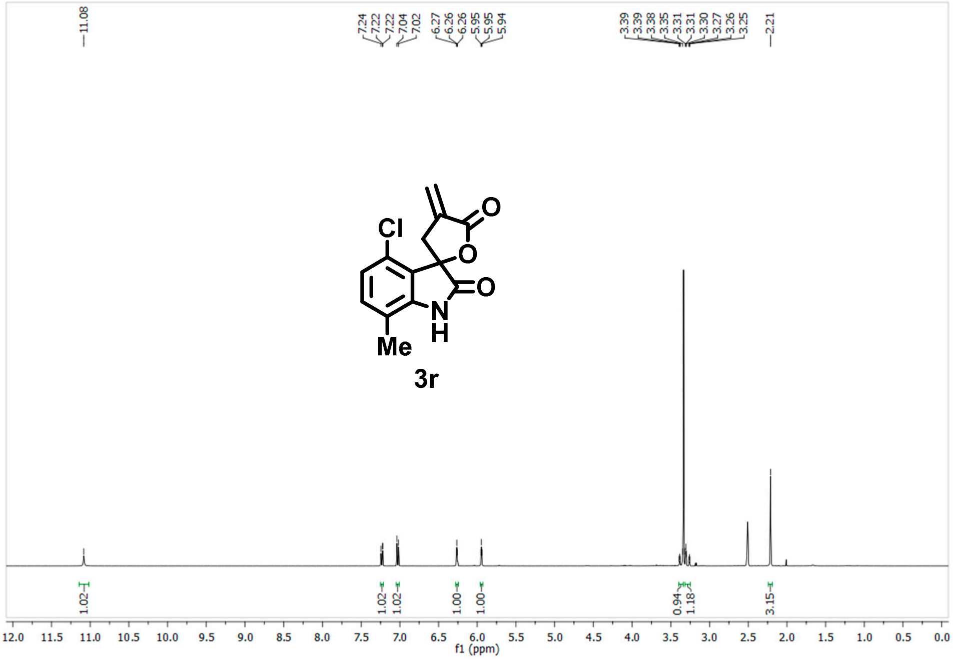

^{1}H NMR of compound **3r** at 400 MHz (DMSO-d_6)

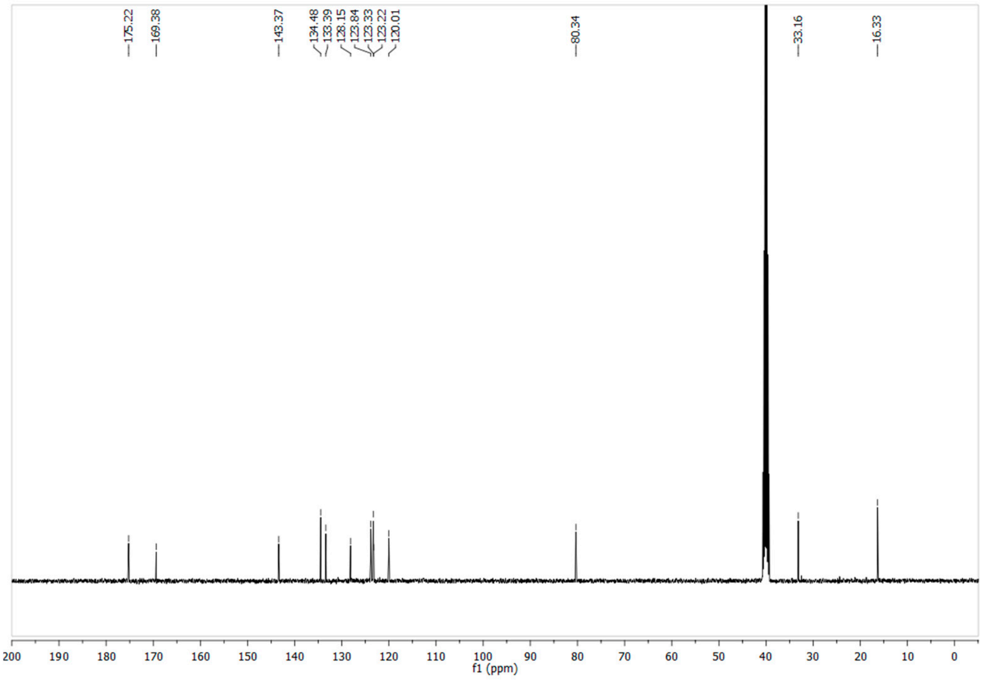

^{13}C NMR of compound **3r** at 101 MHz (DMSO-d_6)

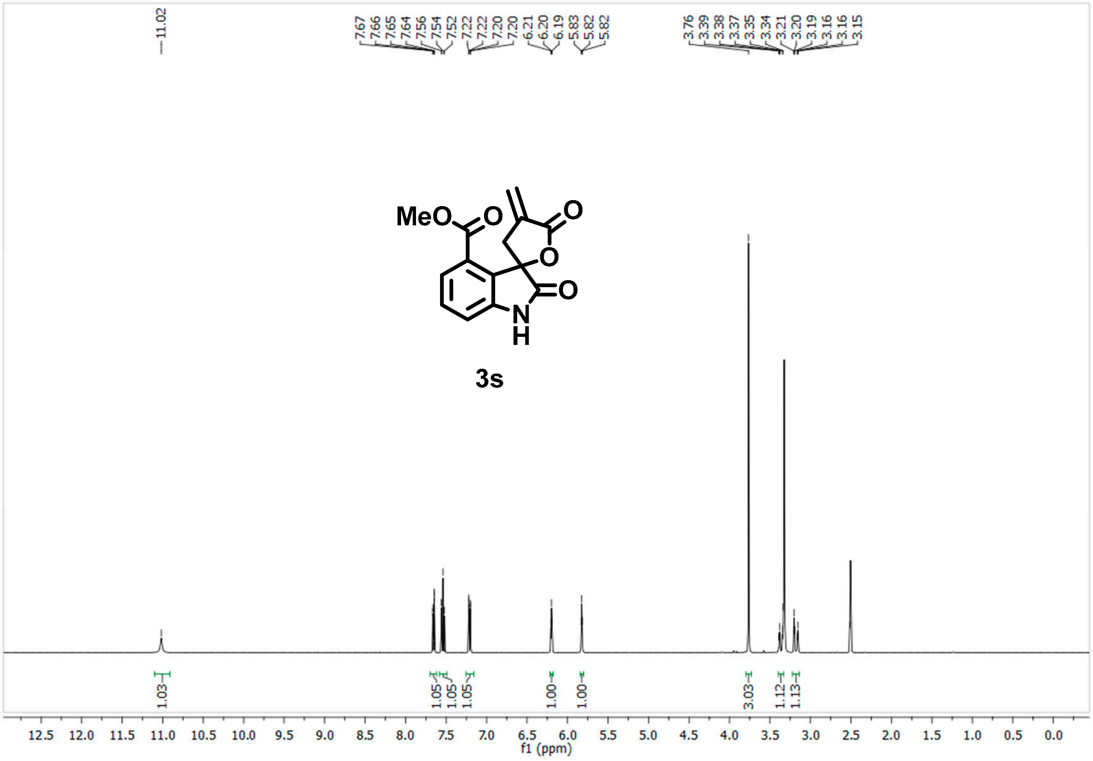

^{1}H NMR of compound **3s** at 400 MHz (DMSO-d_6)

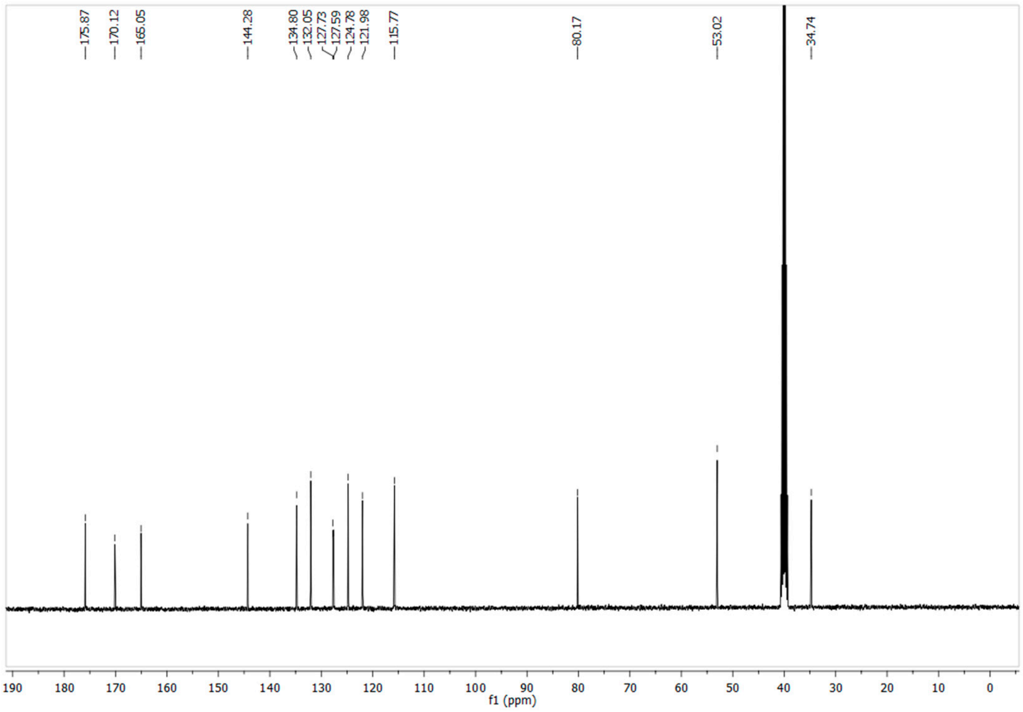

^{13}C NMR of compound **3s** at 101 MHz (DMSO-d_6)

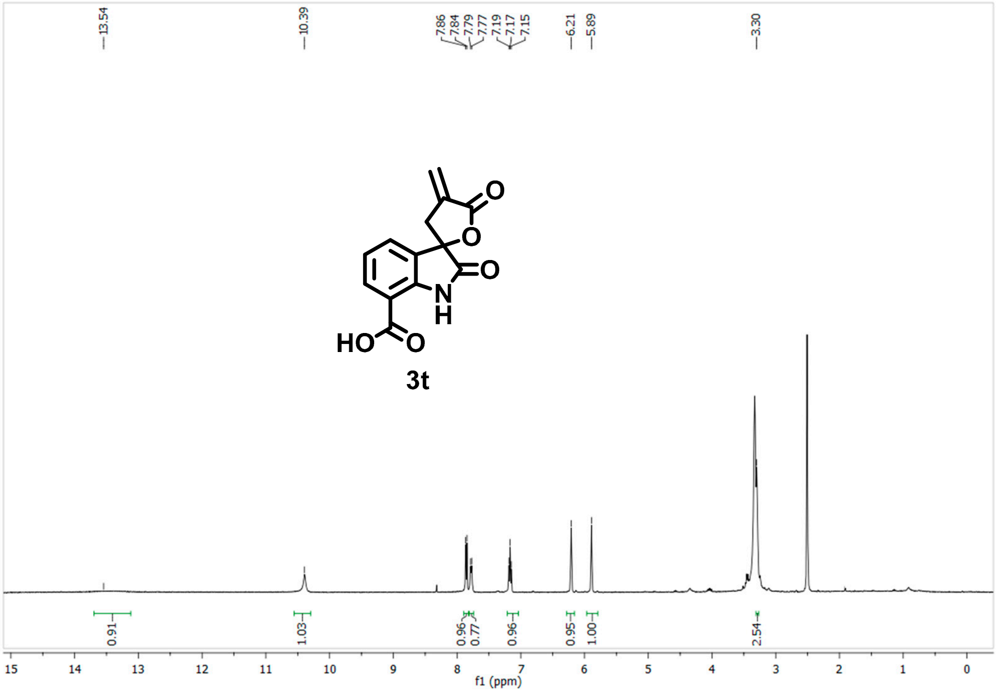

^{1}H NMR of compound **3t** at 400 MHz (DMSO-d_6)

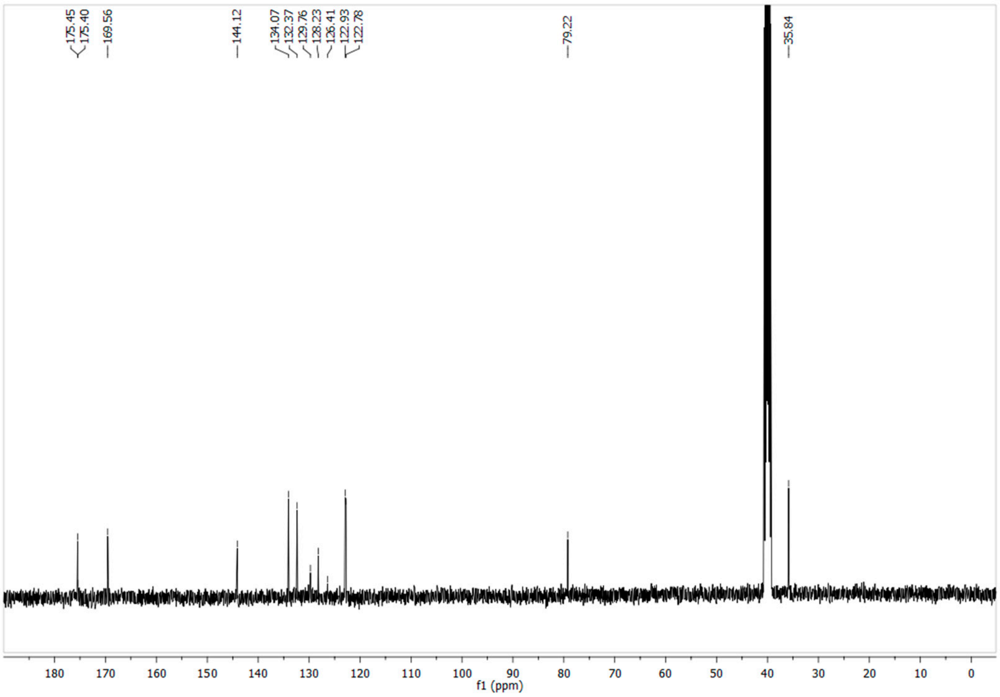

^{13}C NMR of compound **3t** at 101 MHz (DMSO-d_6)

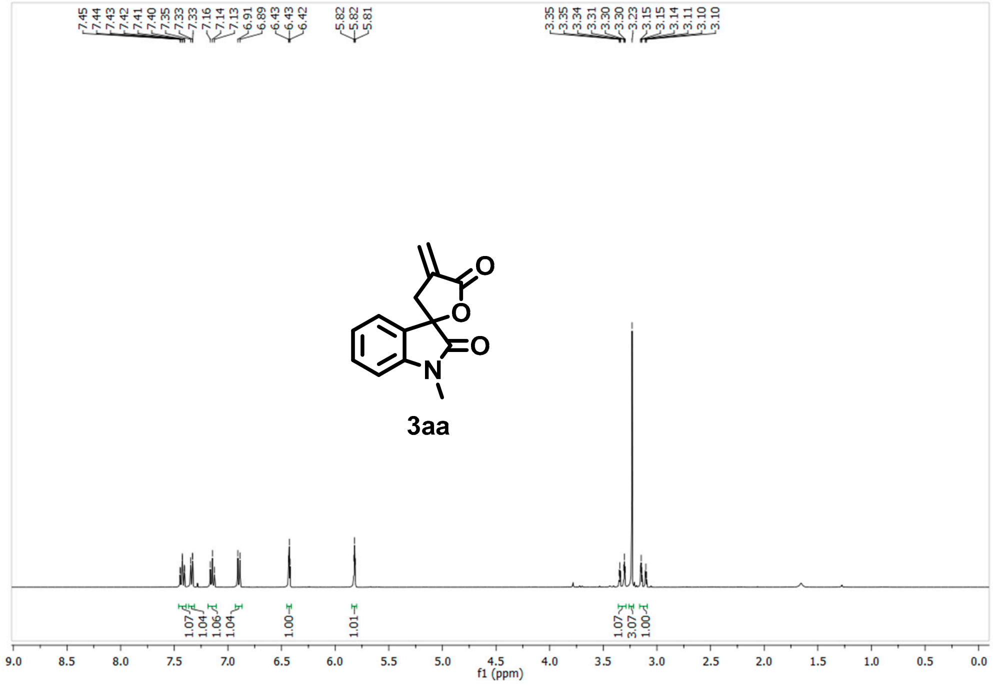

^{1}H NMR of compound **3aa** at 400 MHz ($CDCl_3$)

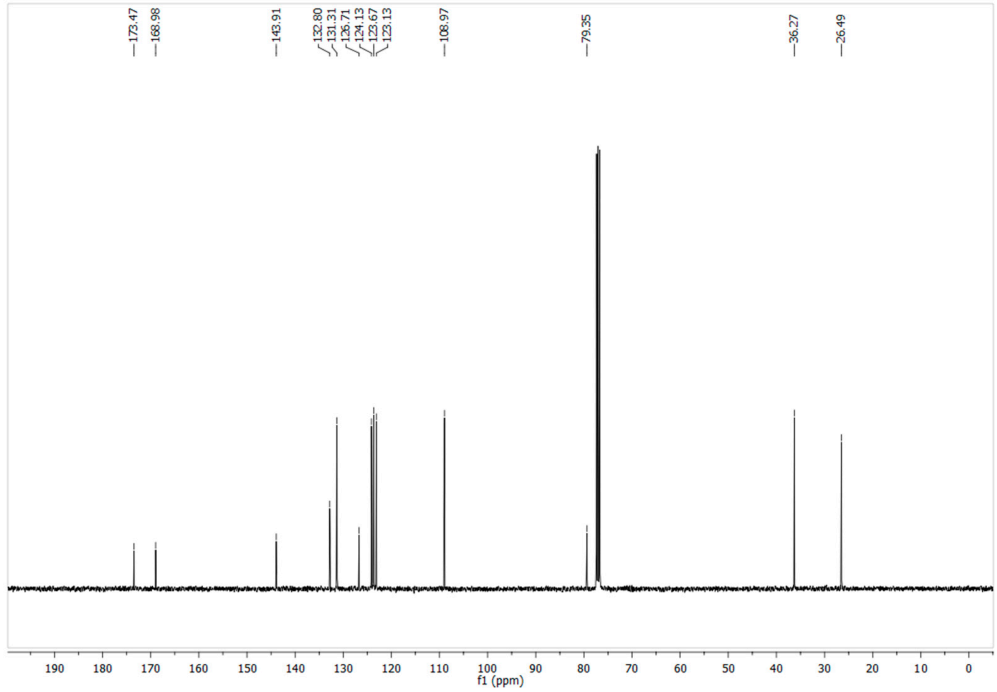

^{13}C NMR of compound **3aa** at 101 MHz ($CDCl_3$)

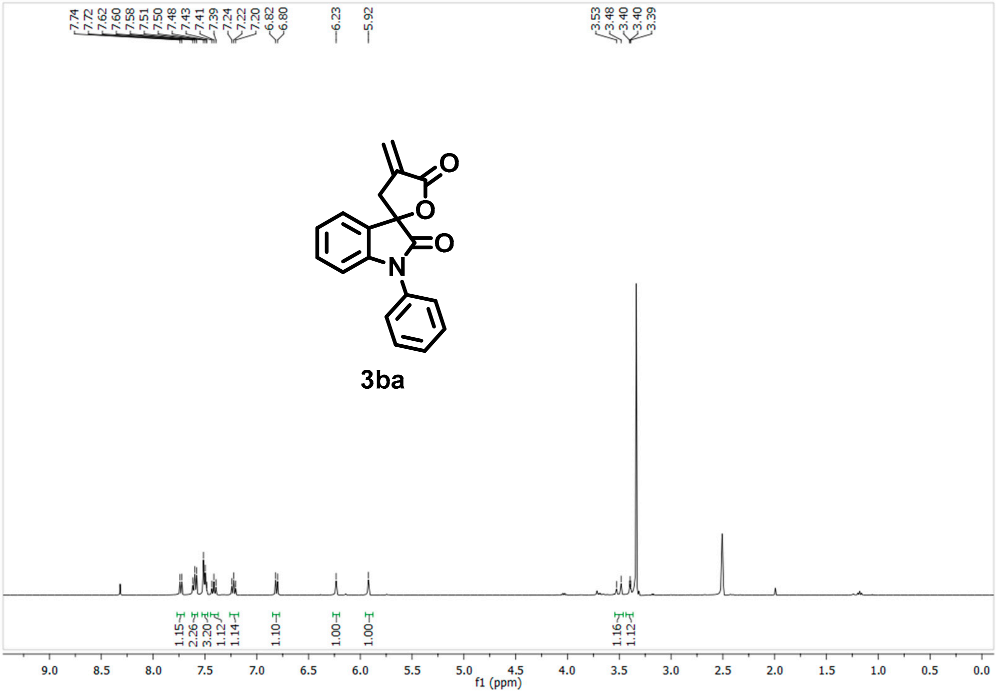

^{1}H NMR of compound **3ba** at 400 MHz (DMSO-d_6)

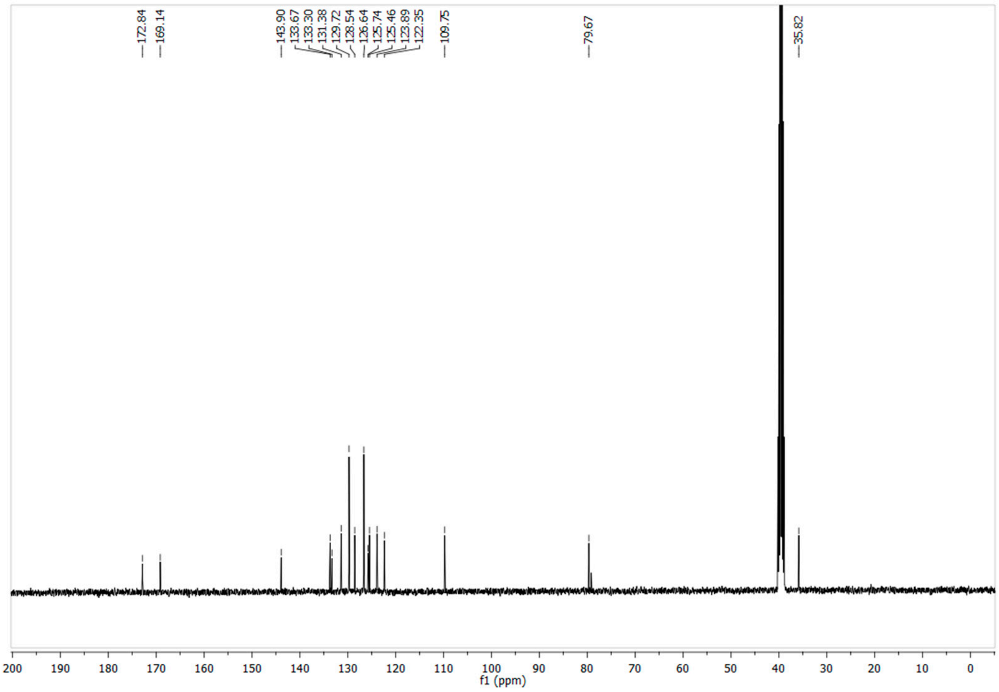

^{13}C NMR of compound **3ba** at 101 MHz (DMSO-d_6)

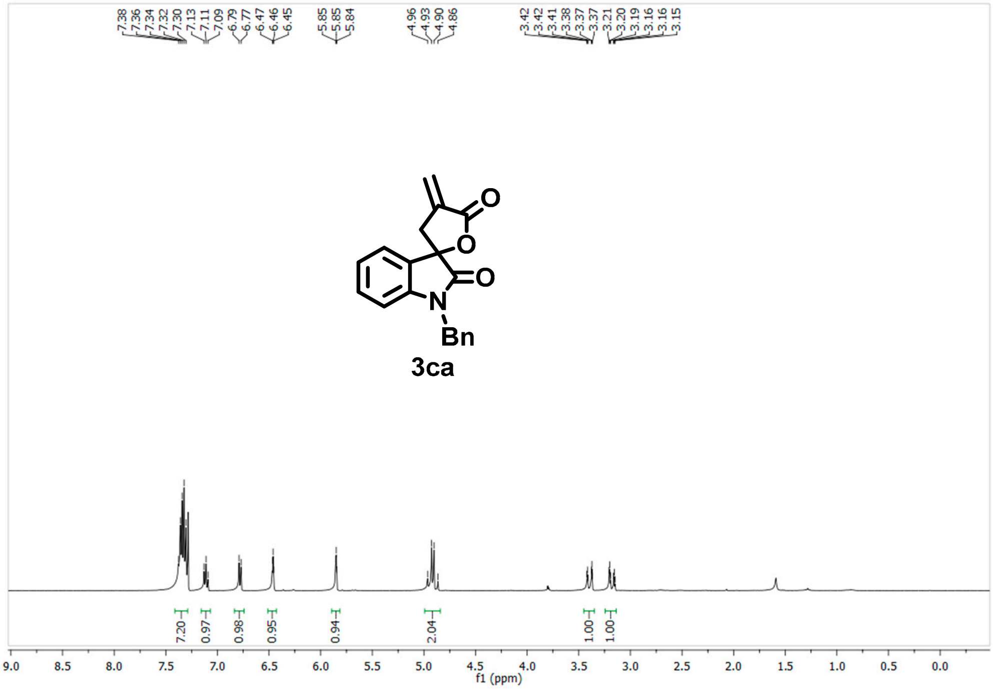

^{1}H NMR of compound **3ca** at 400 MHz ($CDCl_3$)

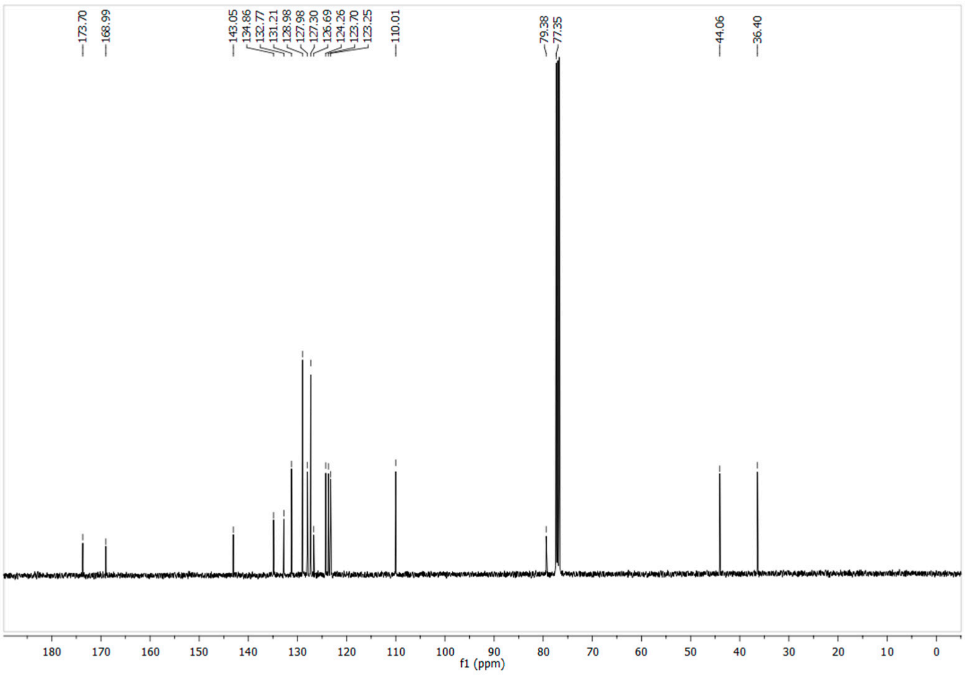

^{13}C NMR of compound **3ca** at 101 MHz ($CDCl_3$)

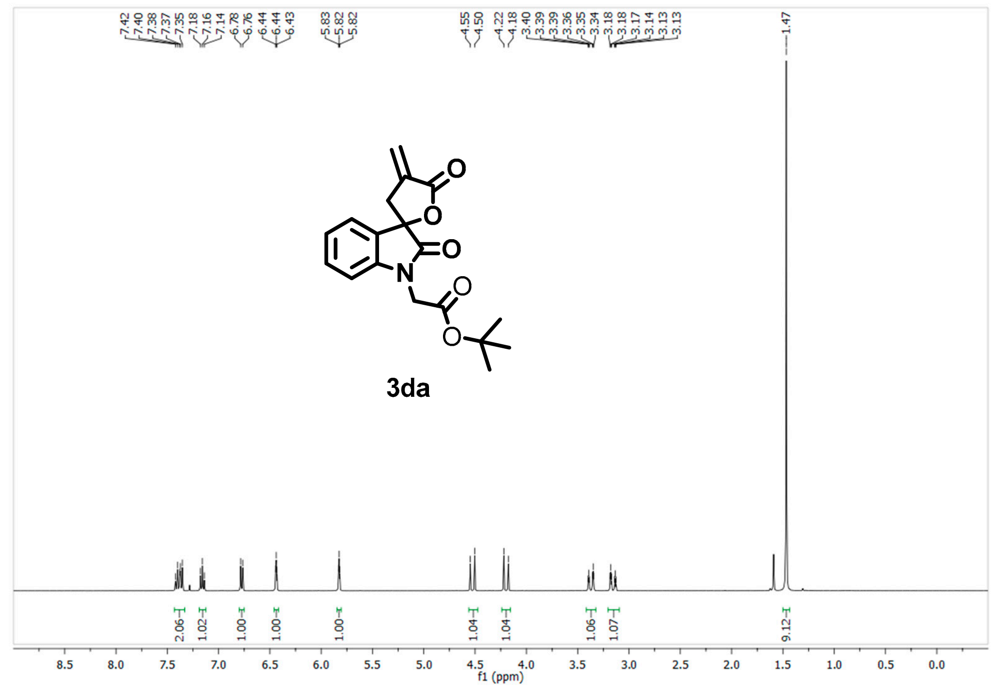

^{1}H NMR of compound **3da** at 400 MHz ($CDCl_3$)

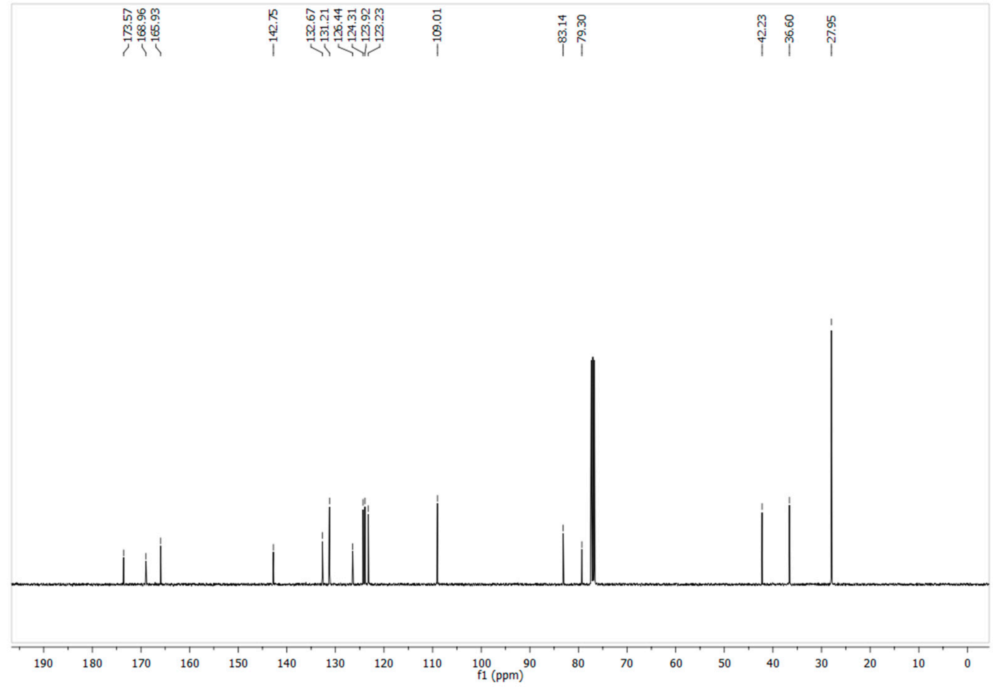

^{13}C NMR of compound **3da** at 101 MHz ($CDCl_3$)

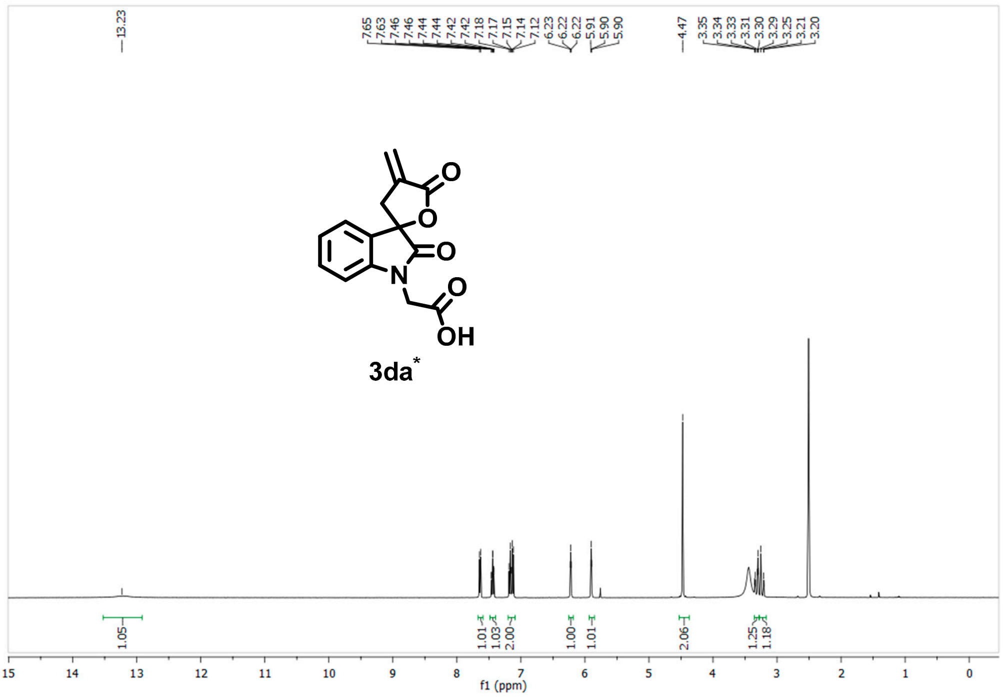

^{1}H NMR of compound **3da*** at 400 MHz (DMSO-d_6)

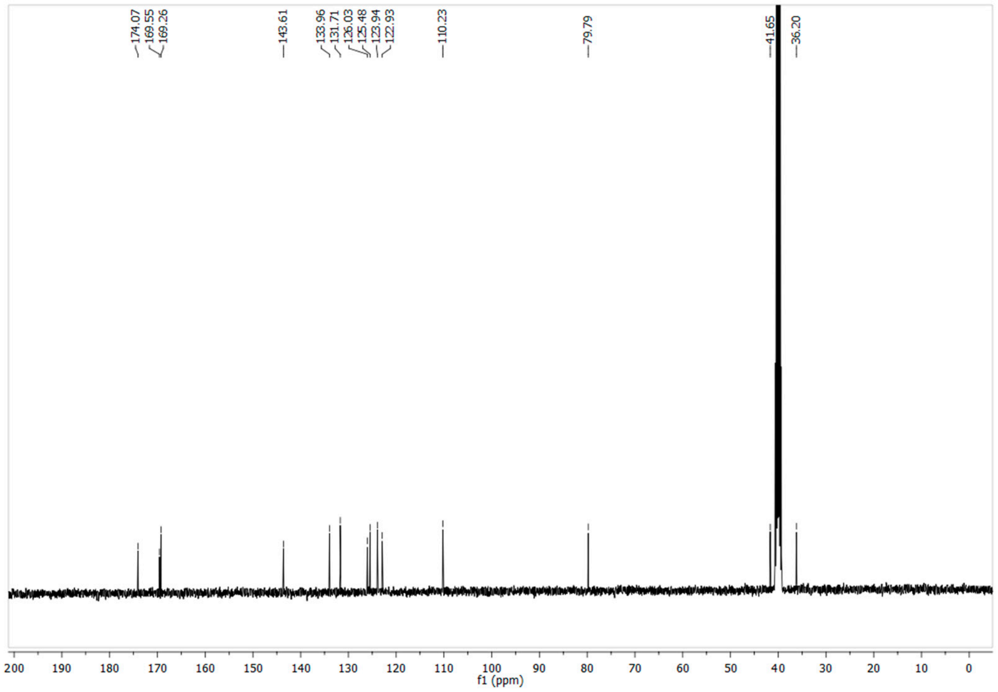

^{13}C NMR of compound **3da*** at 101 MHz (DMSO-d_6)

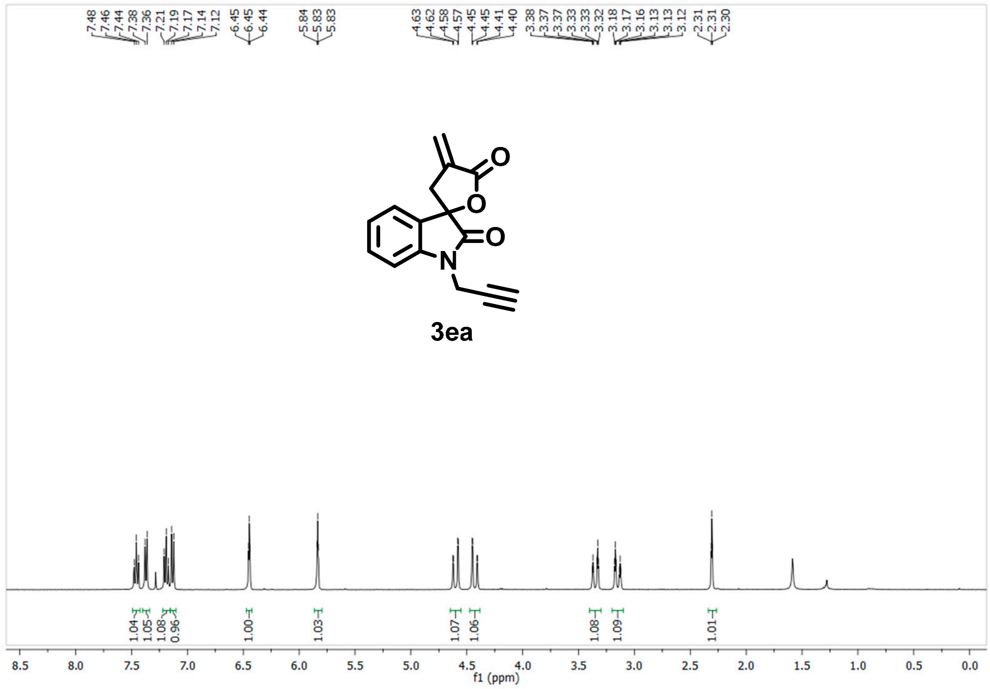

^{1}H NMR of compound **3ea** at 400 MHz ($CDCl_3$)

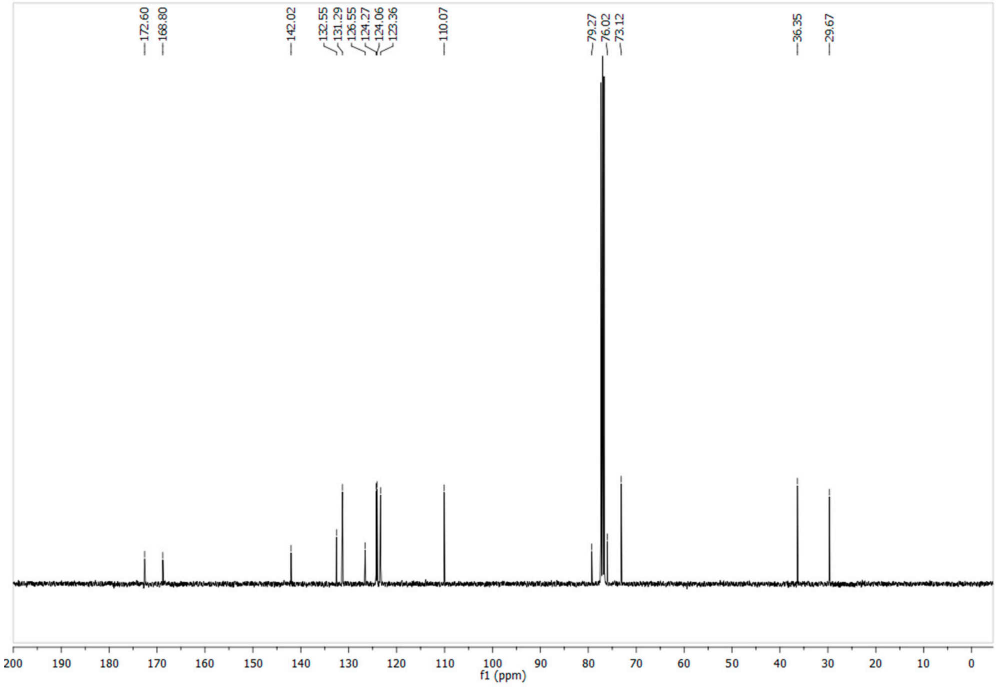

^{13}C NMR of compound **3ea** at 101 MHz ($CDCl_3$)

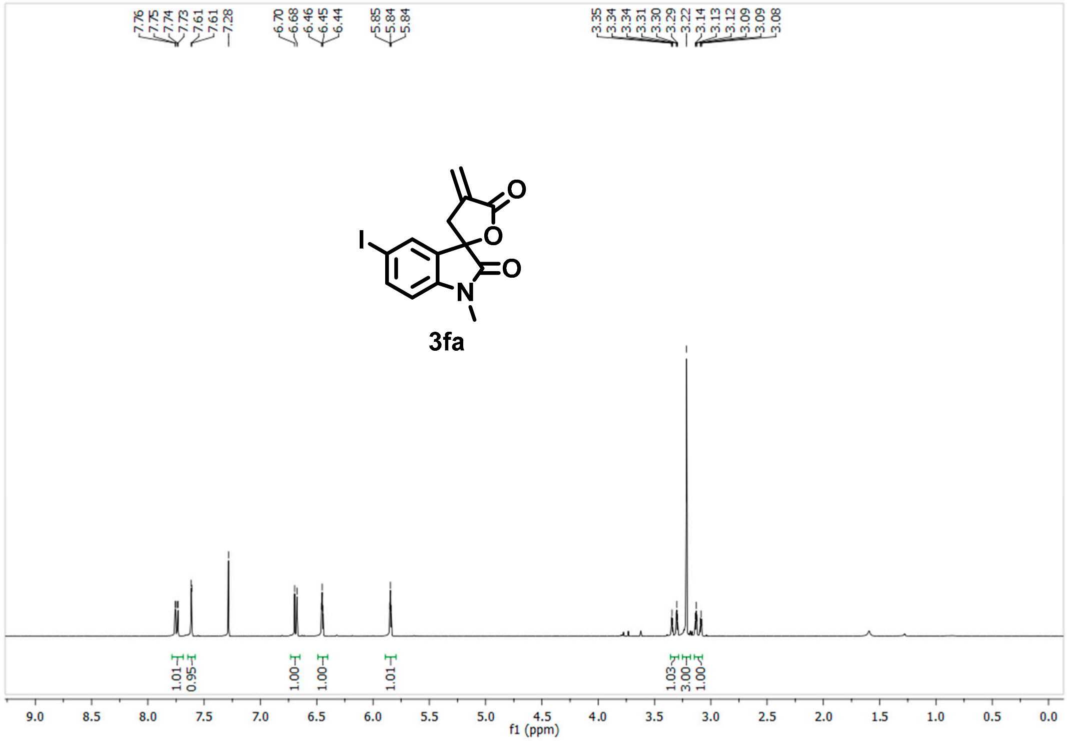

^{1}H NMR of compound **3fa** at 400 MHz ($CDCl_3$)

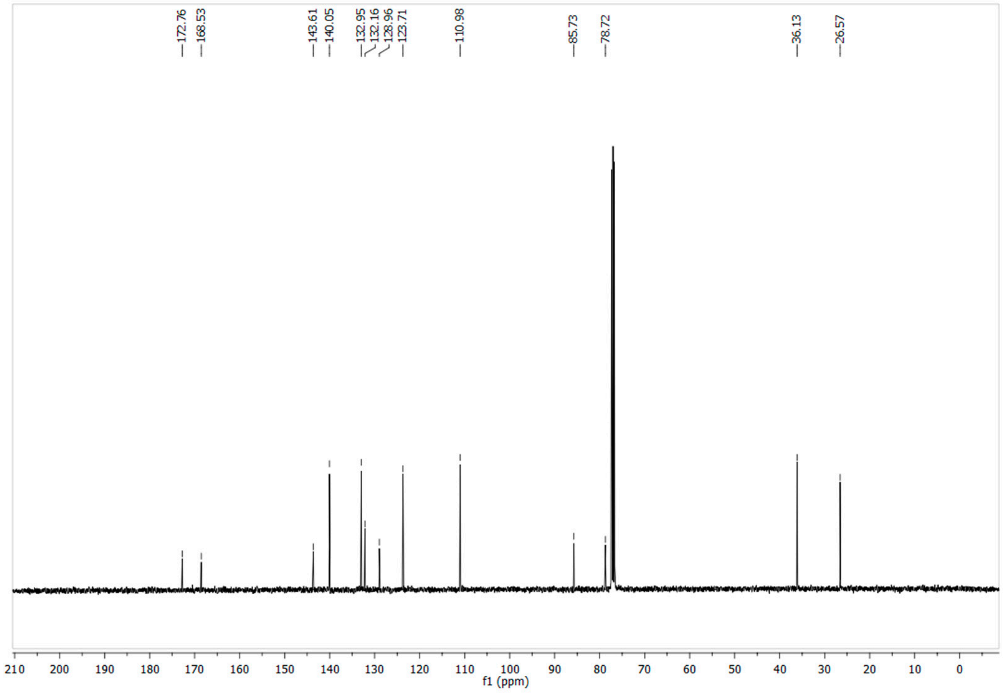

^{13}C NMR of compound **3fa** at 101 MHz ($CDCl_3$)

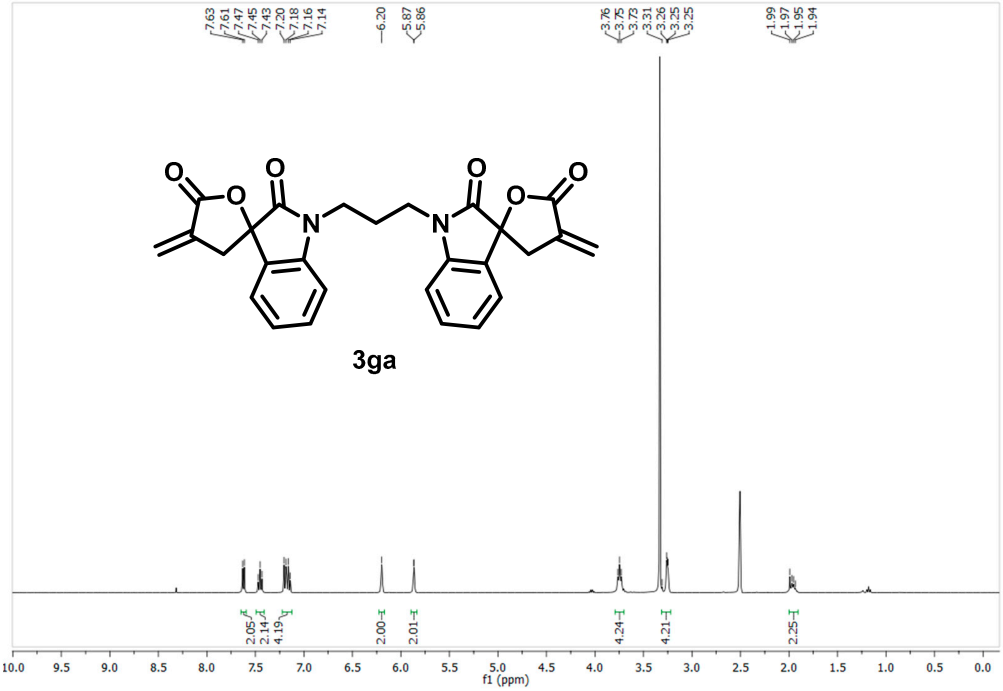

^{1}H NMR of compound **3ga** at 400 MHz (DMSO-d_6)

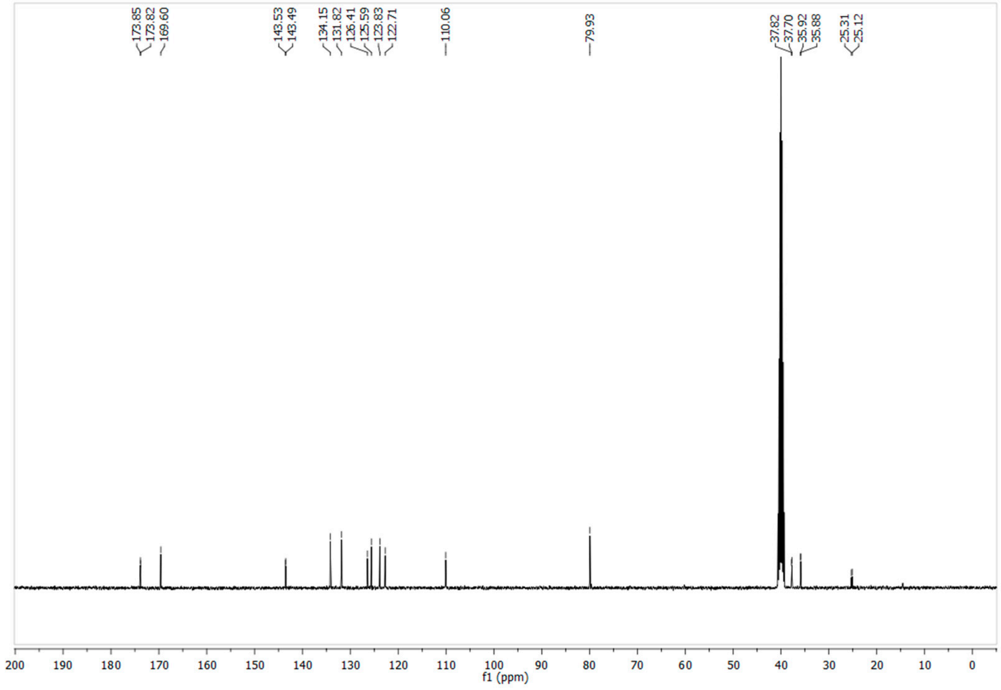

^{13}C NMR of compound **3ga** at 101 MHz (DMSO-d_6)

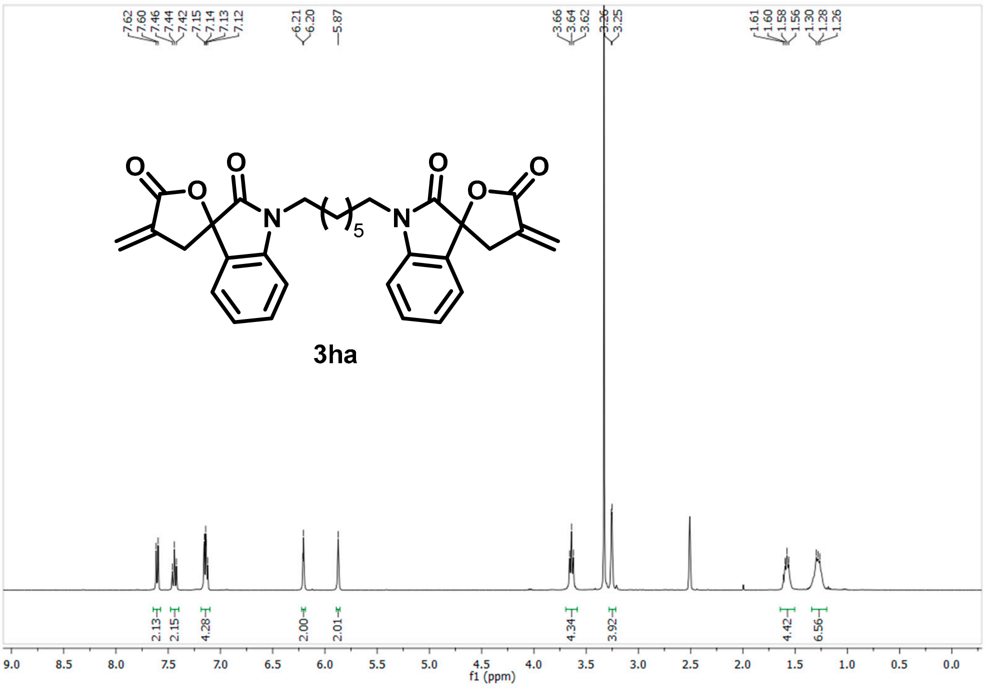

^{1}H NMR of compound **3ha** at 400 MHz (DMSO-d_6)

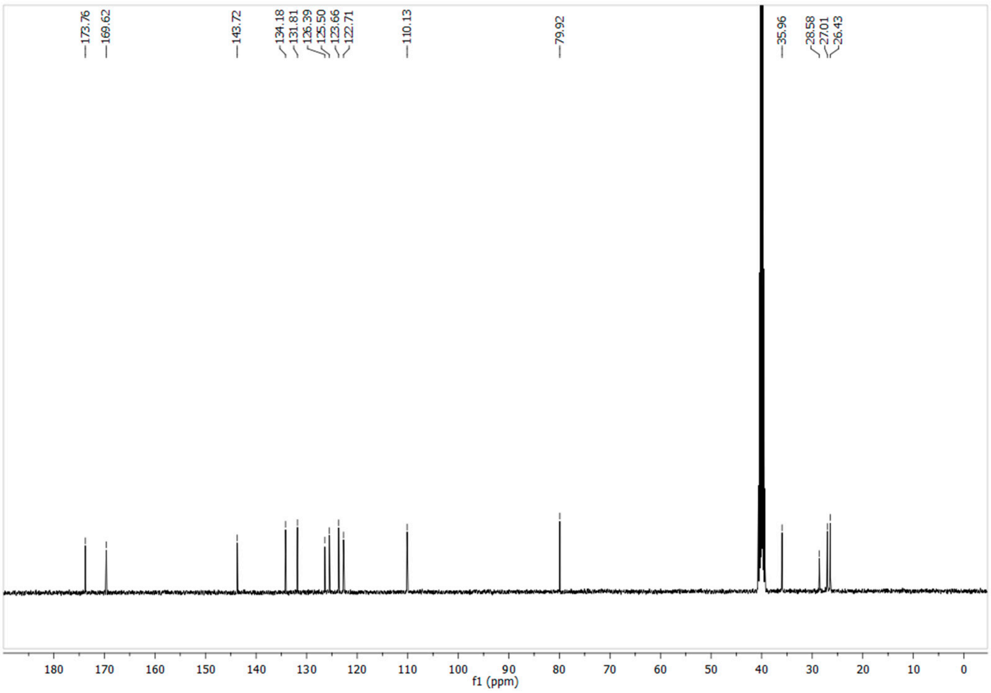

^{13}C NMR of compound **3ha** at 101 MHz (DMSO-d_6)

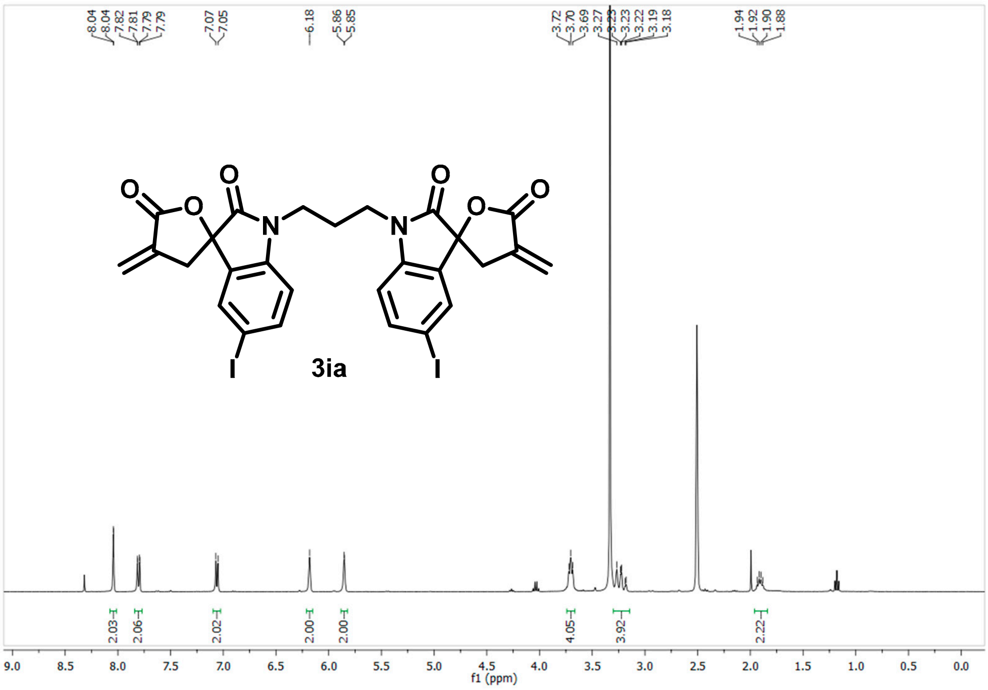

^{1}H NMR of compound **3ia** at 400 MHz (DMSO-d_6)

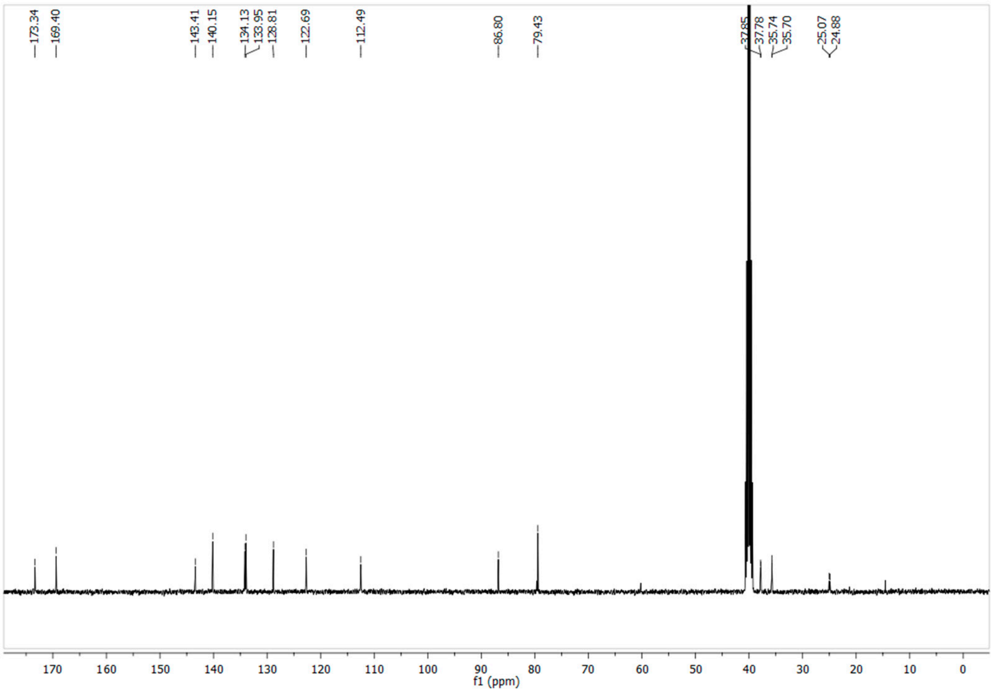

^{13}C NMR of compound **3ia** at 101 MHz (DMSO-d_6)

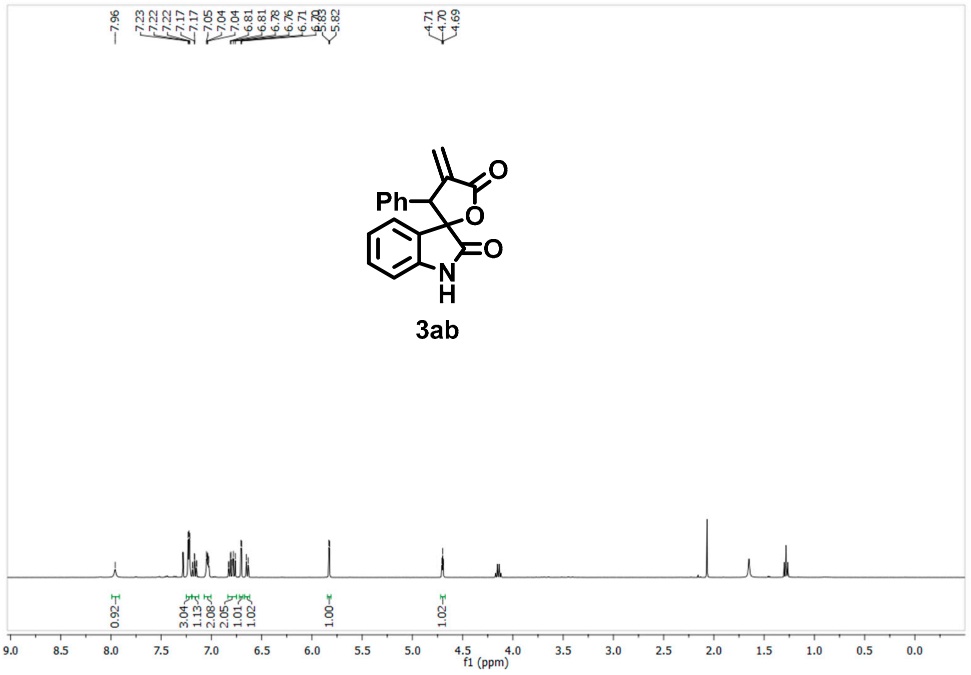

^{1}H NMR of compound **3ab** at 400 MHz ($CDCl_3$)

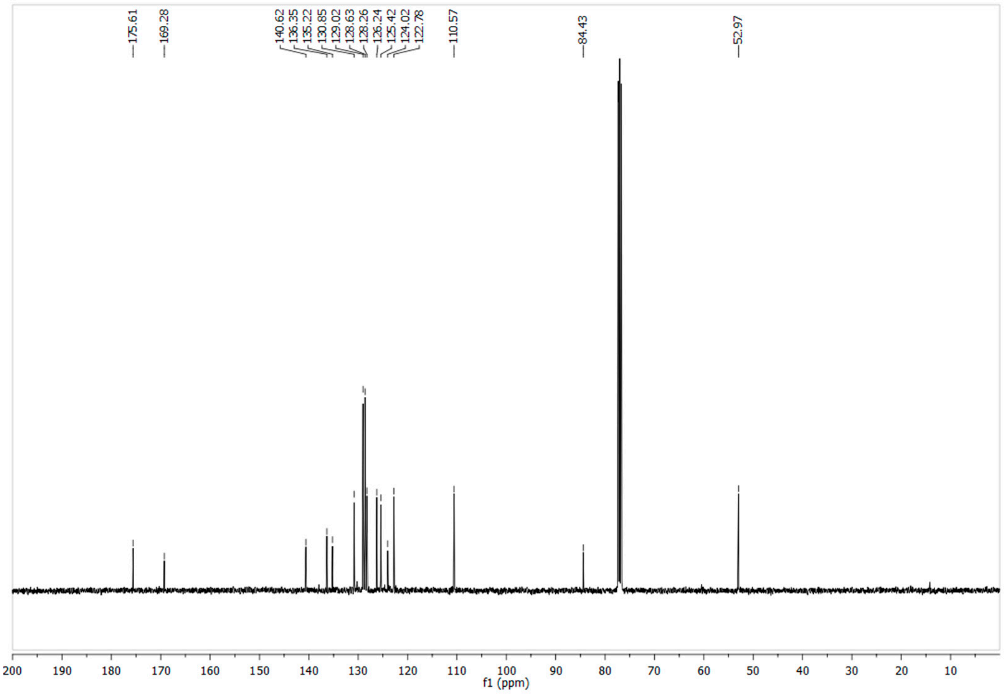

^{13}C NMR of compound **3ab** at 101 MHz ($CDCl_3$)

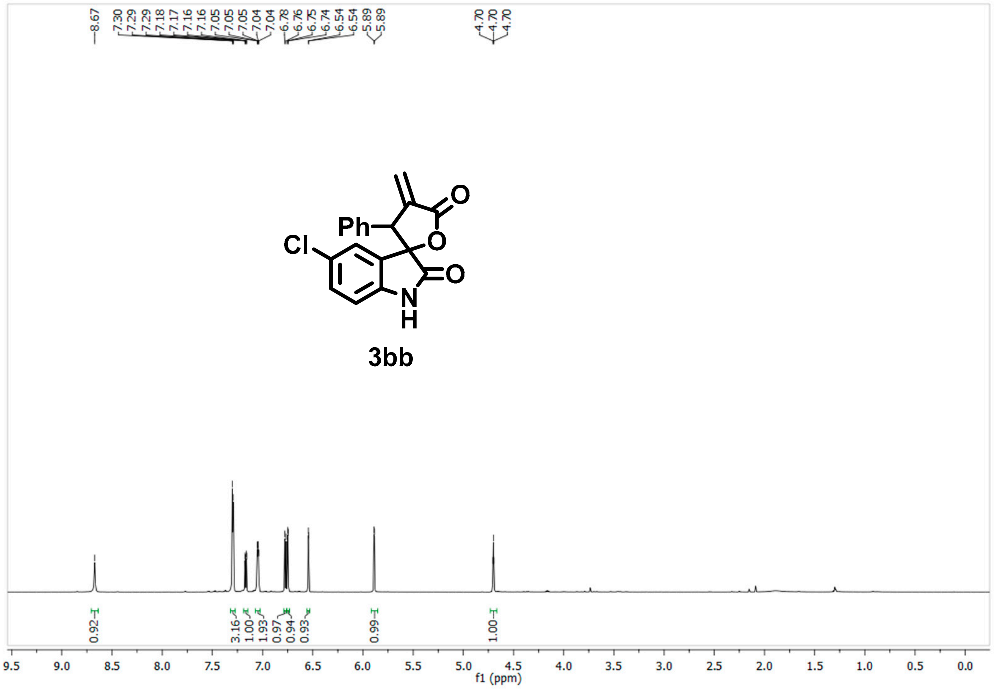

^{1}H NMR of compound **3bb** at 600 MHz ($CDCl_3$)

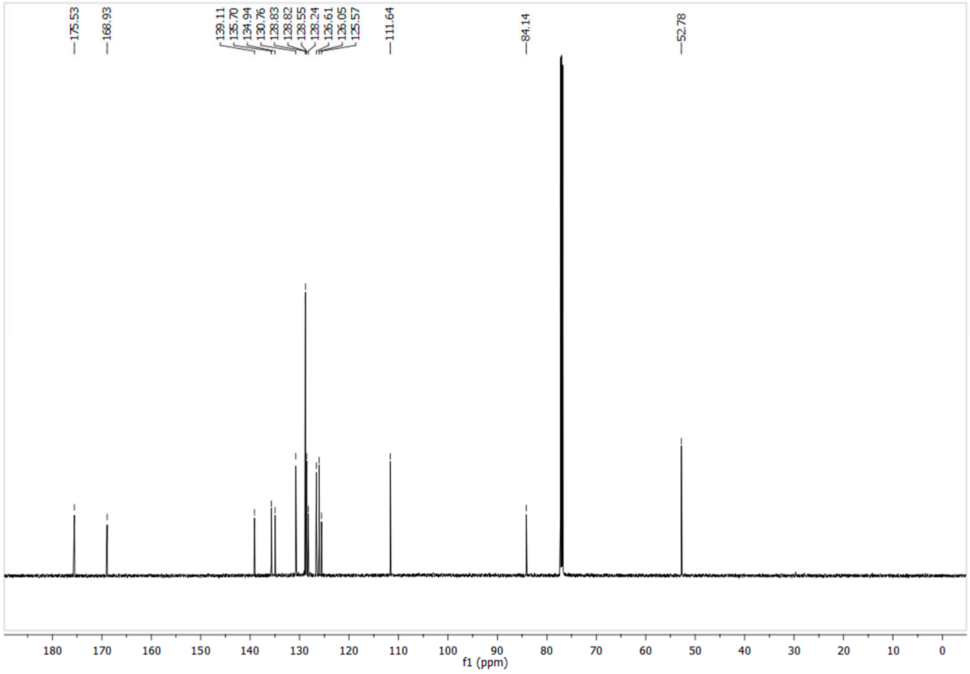

^{13}C NMR of compound **3bb** at 151 MHz ($CDCl_3$)

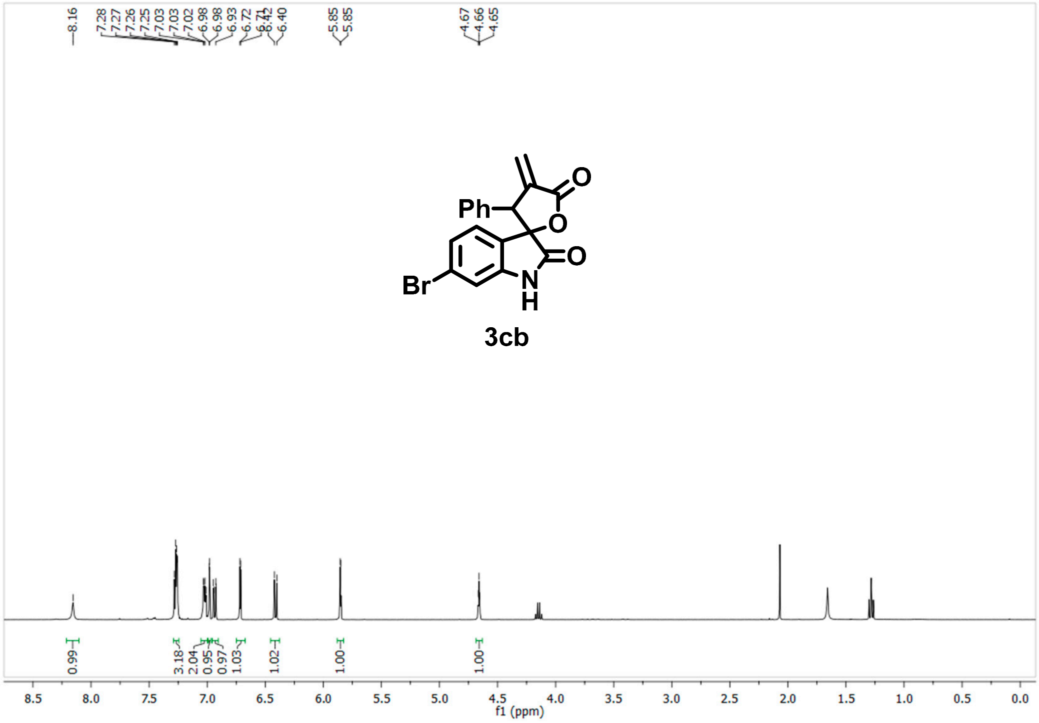

^{1}H NMR of compound **3cb** at 400 MHz ($CDCl_3$)

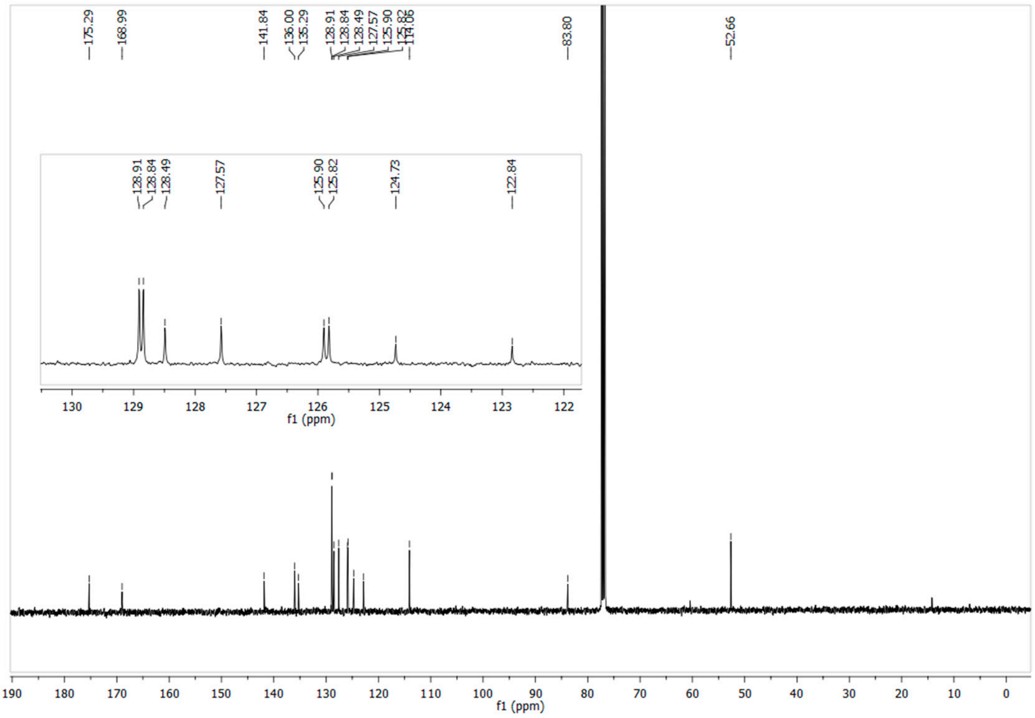

^{13}C NMR of compound **3cb** at 101 MHz ($CDCl_3$)

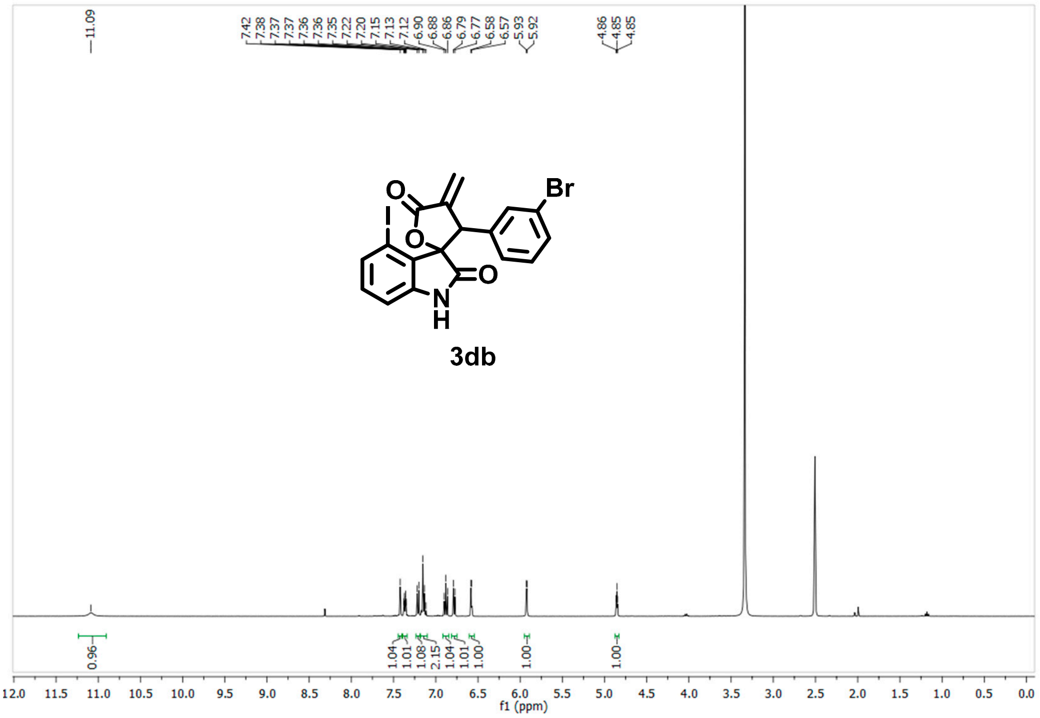

^{1}H NMR of compound **3db** at 400 MHz (DMSO-d_6)

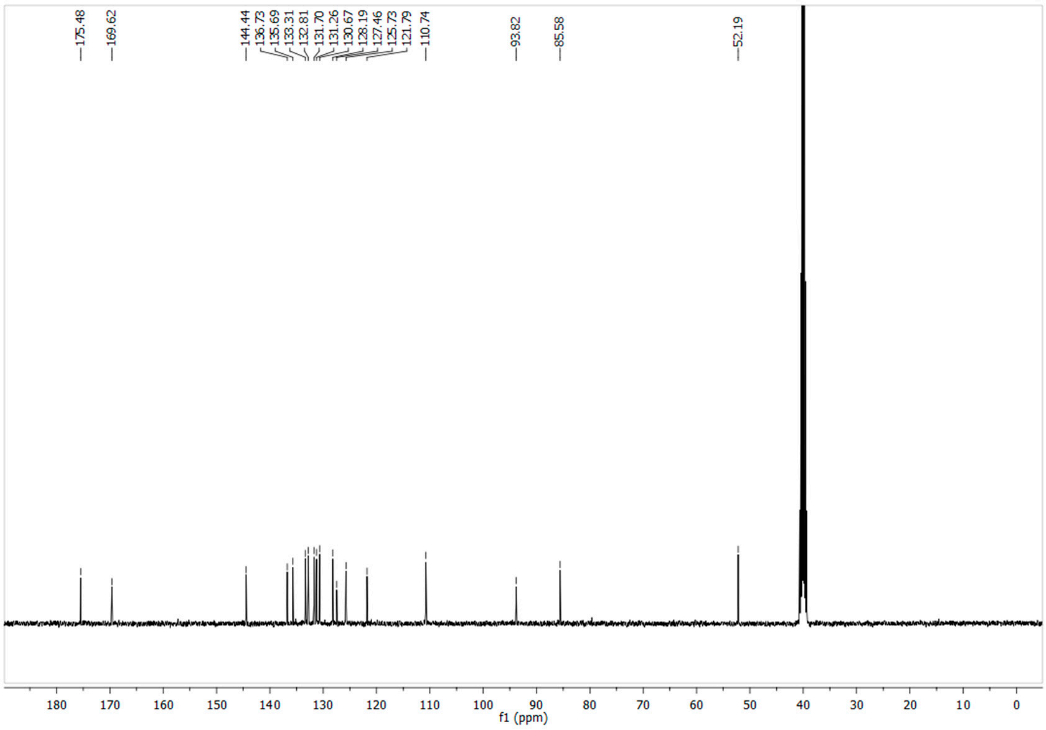

^{13}C NMR of compound **3db** at 101 MHz (DMSO-d_6)

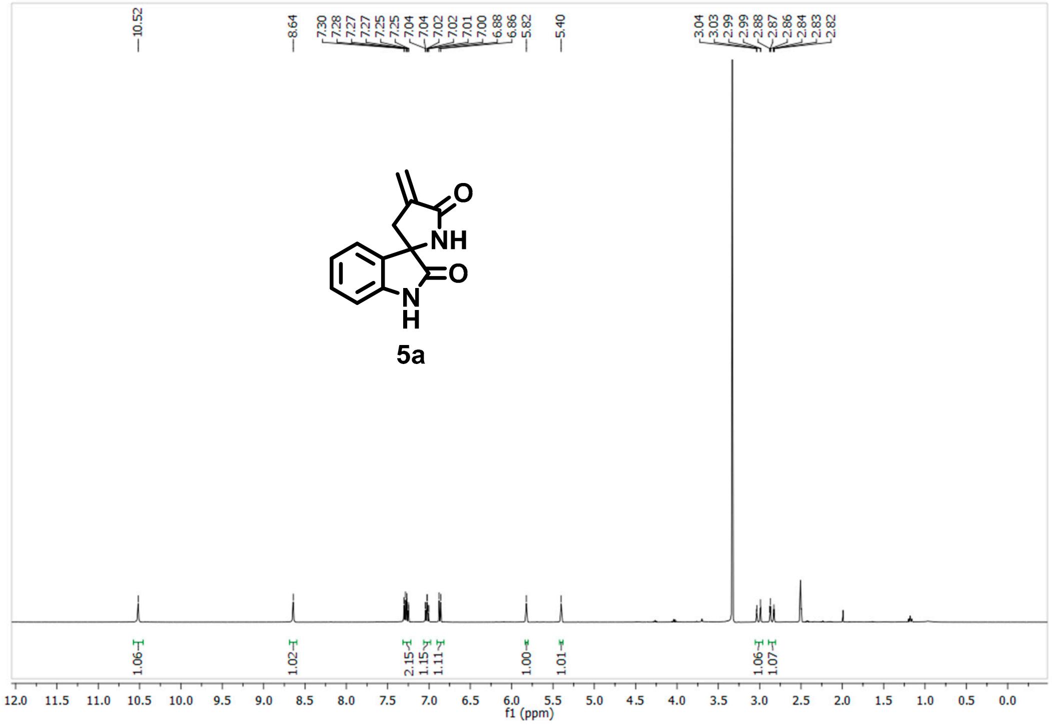

^{1}H NMR of compound **5a** at 400 MHz (DMSO-d_6)

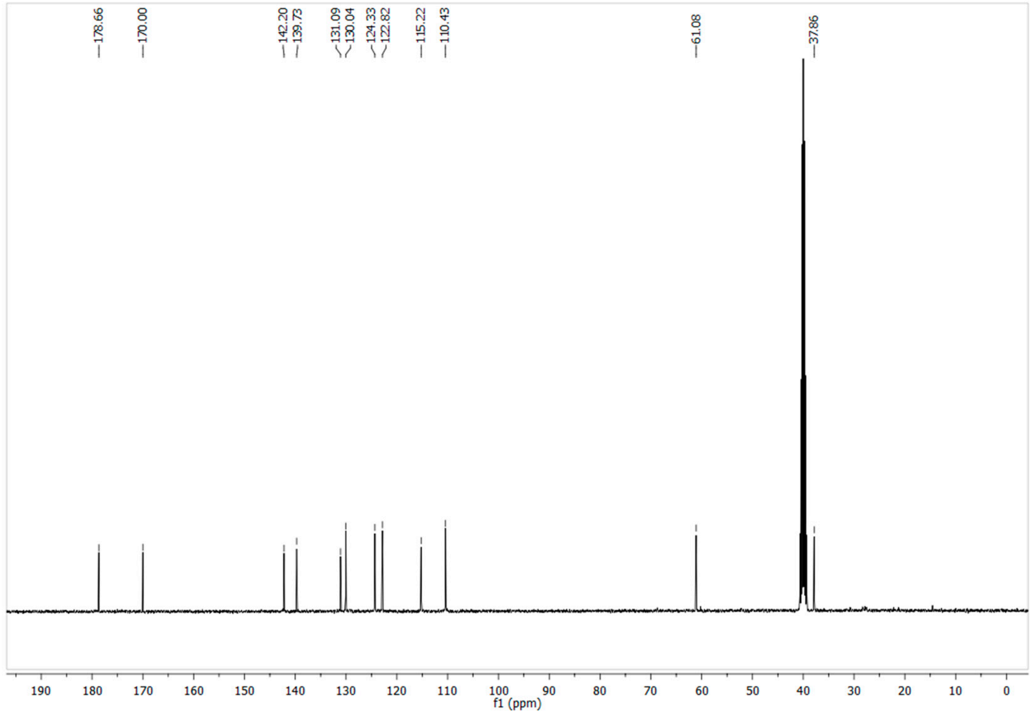

^{13}C NMR of compound **5a** at 101 MHz (DMSO-d_6)

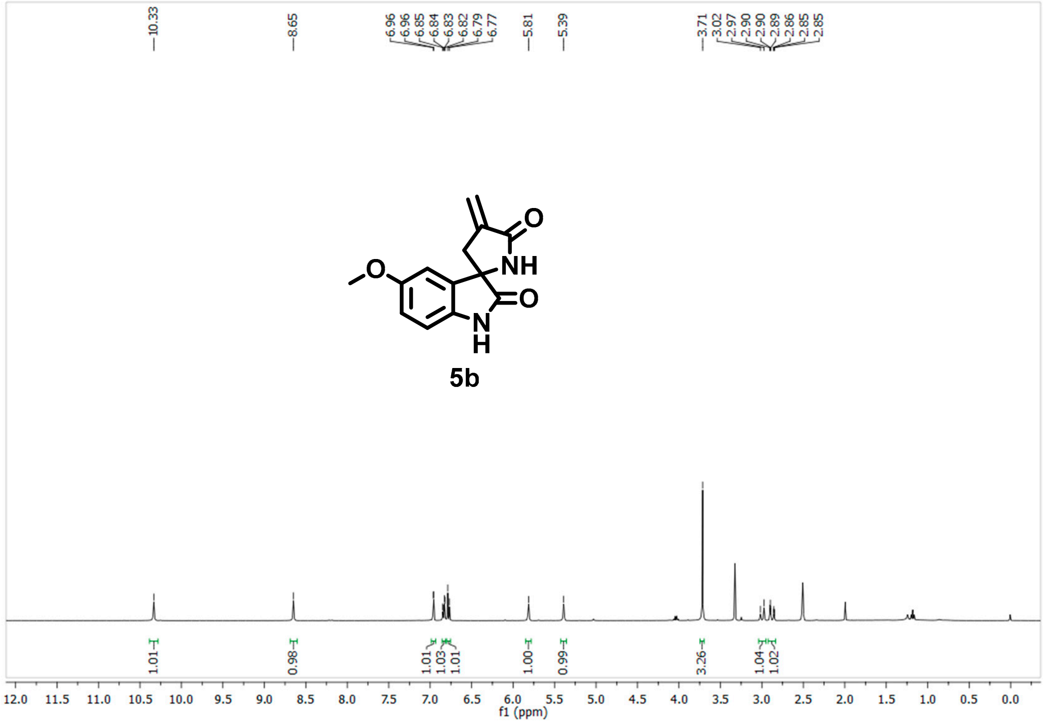

^{1}H NMR of compound **5b** at 400 MHz (DMSO-d_6)

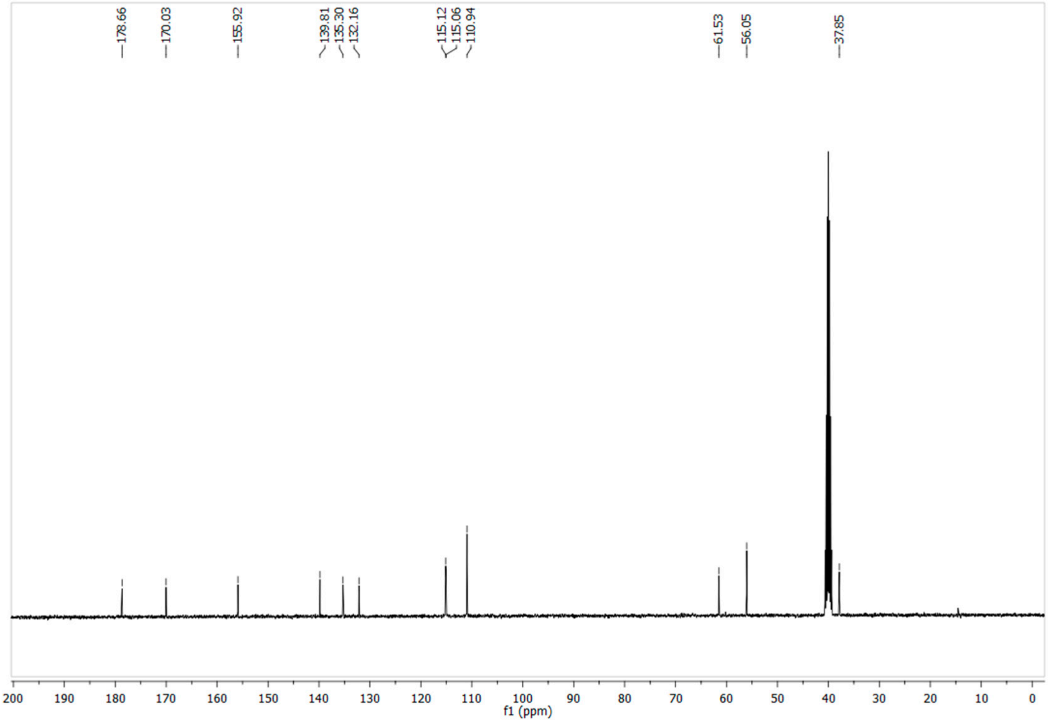

^{13}C NMR of compound **5b** at 101 MHz (DMSO-d_6)

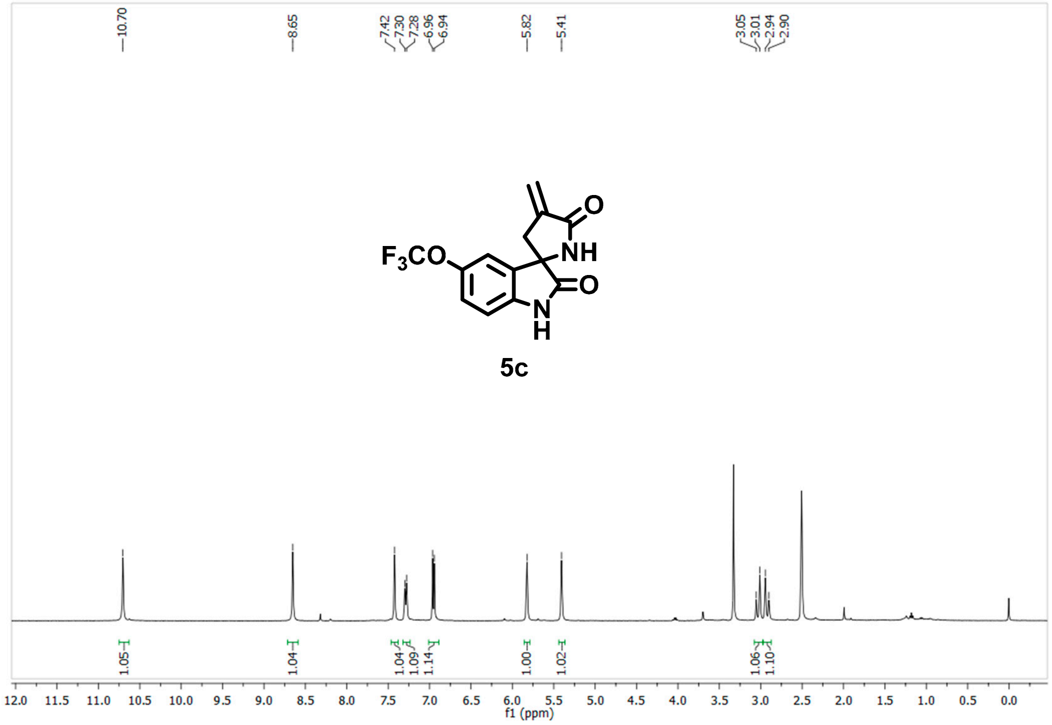

^{1}H NMR of compound **5c** at 400 MHz (DMSO-d_6)

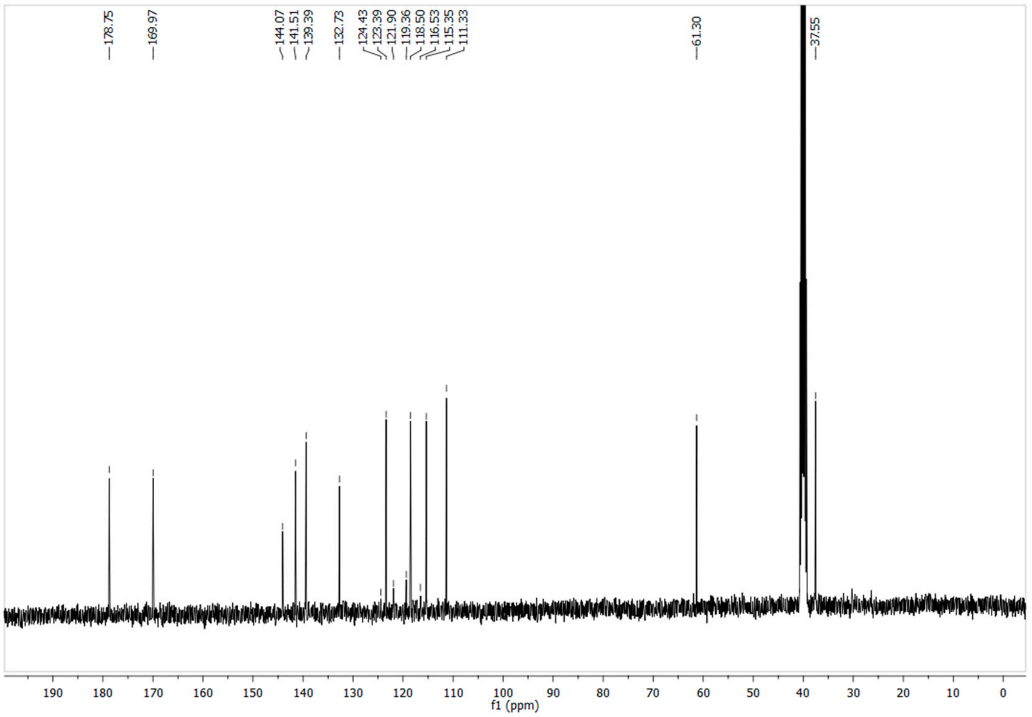

^{13}C NMR of compound **5c** at 101 MHz (DMSO-d_6)

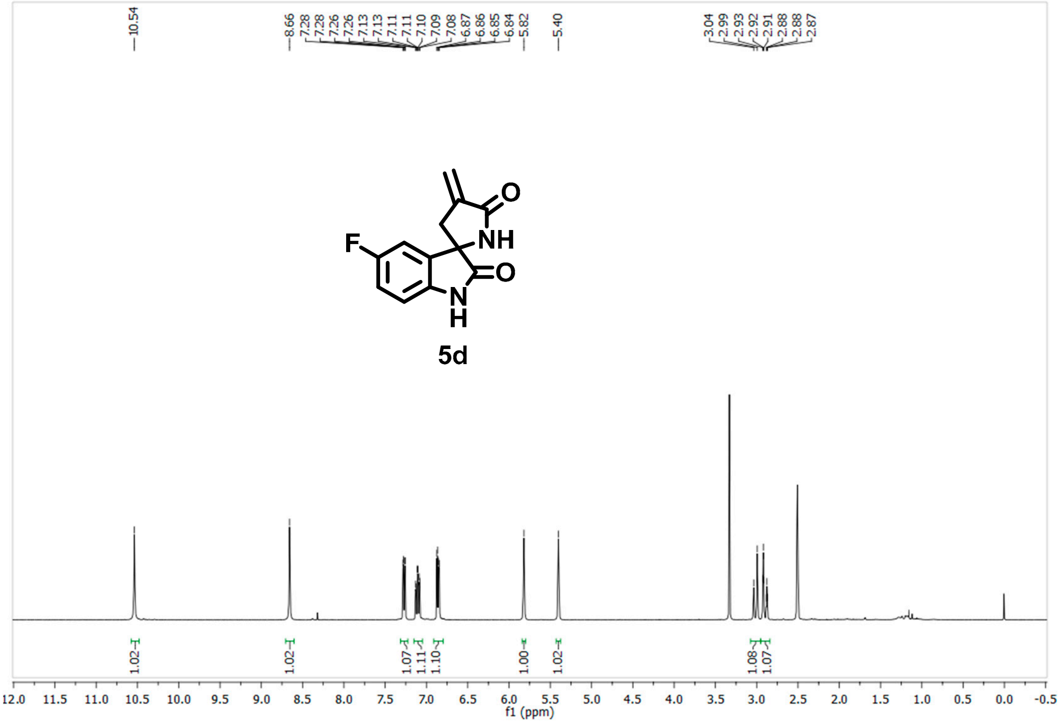

^{1}H NMR of compound **5d** at 400 MHz (DMSO-d_6)

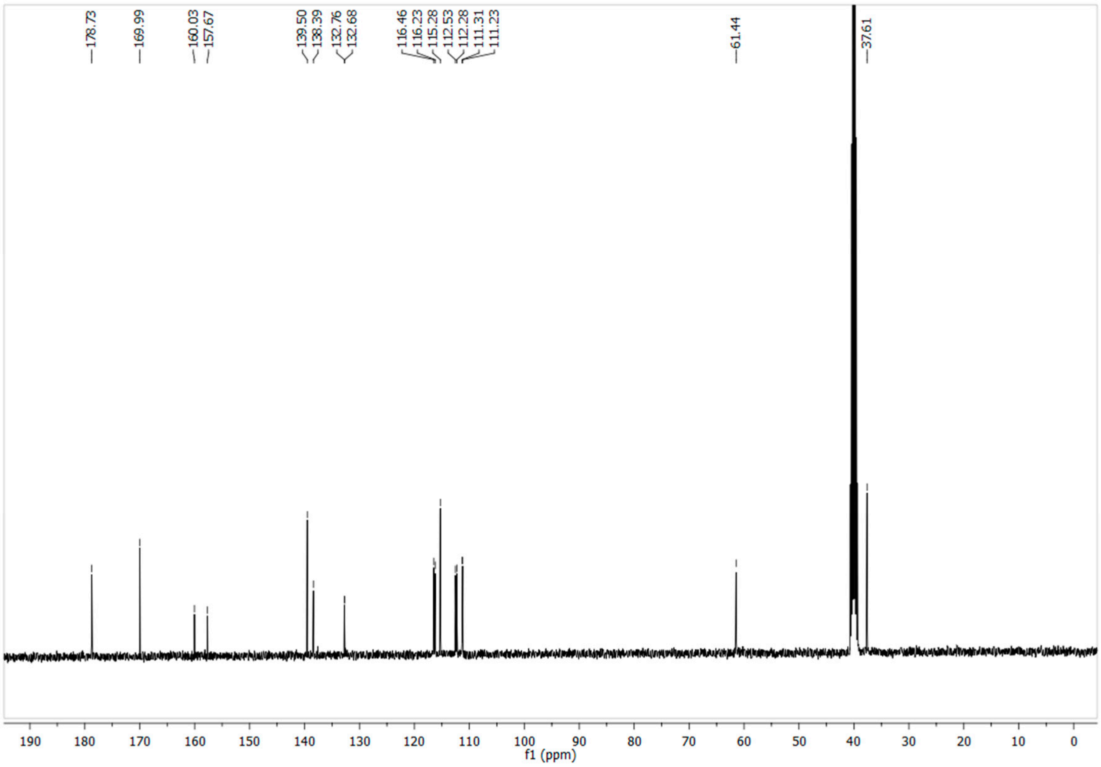

^{13}C NMR of compound **5d** at 101 MHz (DMSO-d_6)

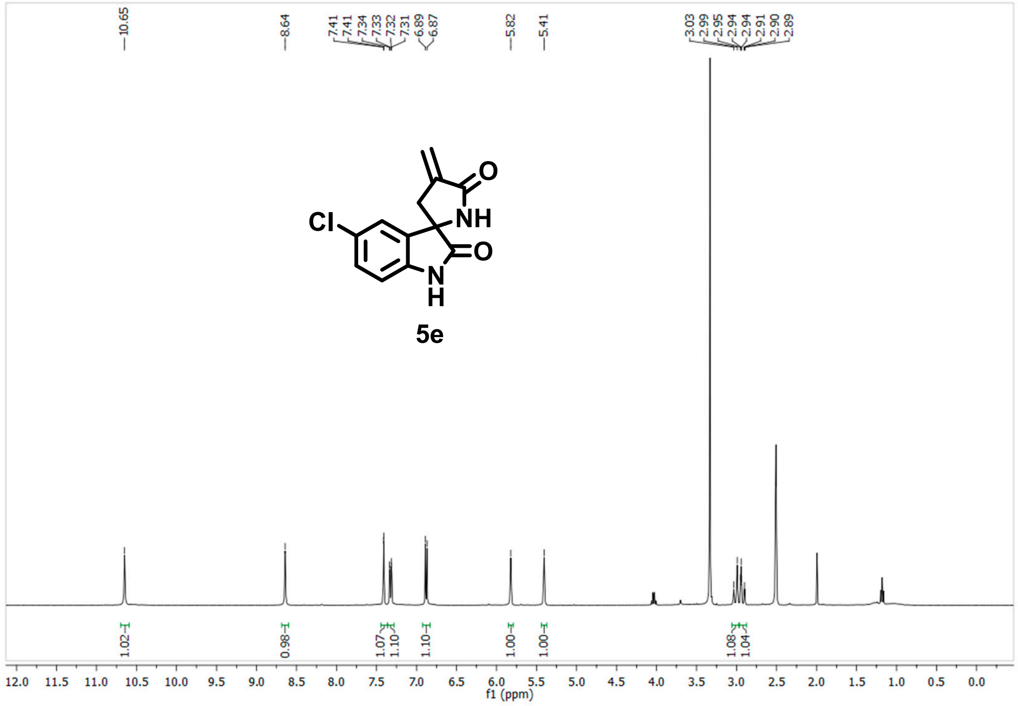

^{1}H NMR of compound **5e** at 400 MHz (DMSO-d_6)

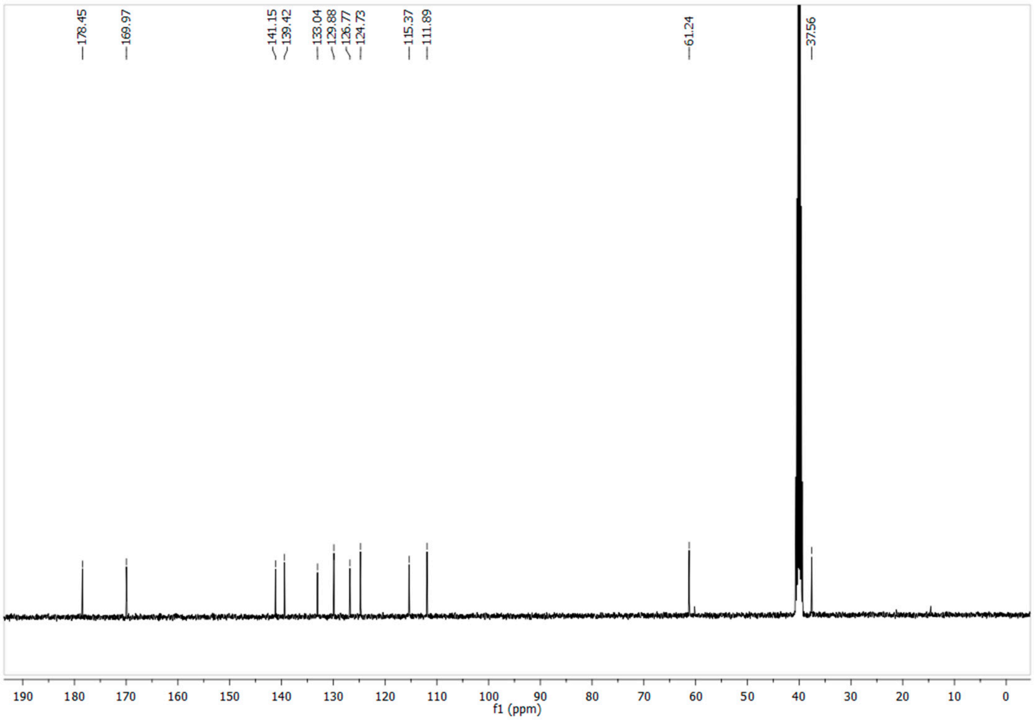

^{13}C NMR of compound **5e** at 101 MHz (DMSO-d_6)

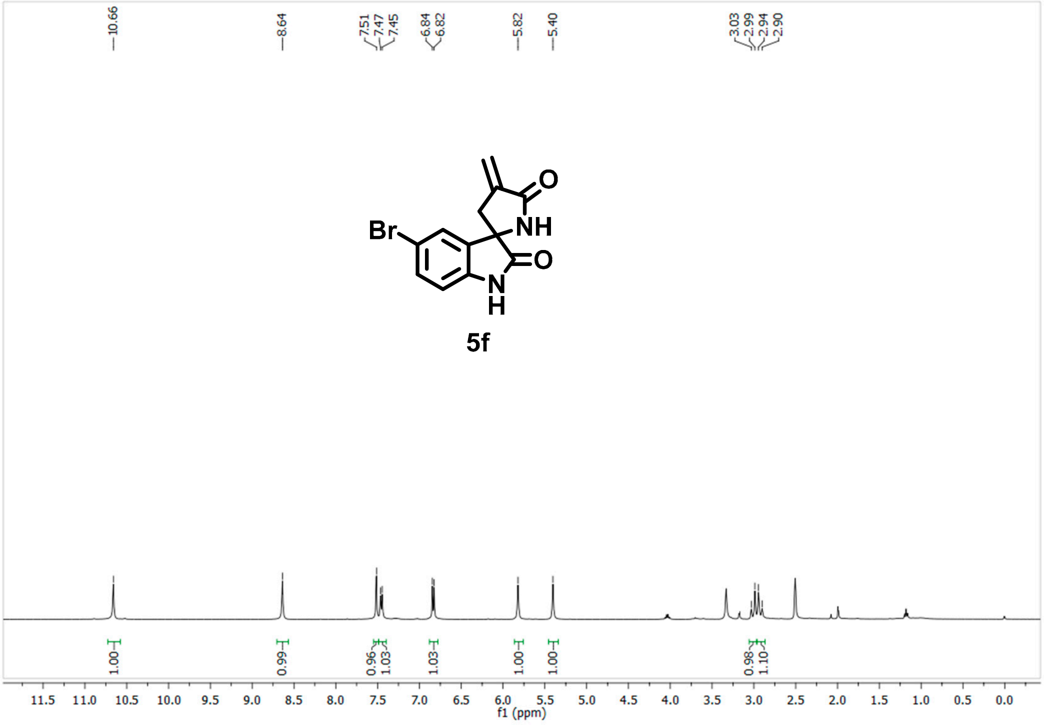

^{1}H NMR of compound **5f** at 400 MHz (DMSO-d_6)

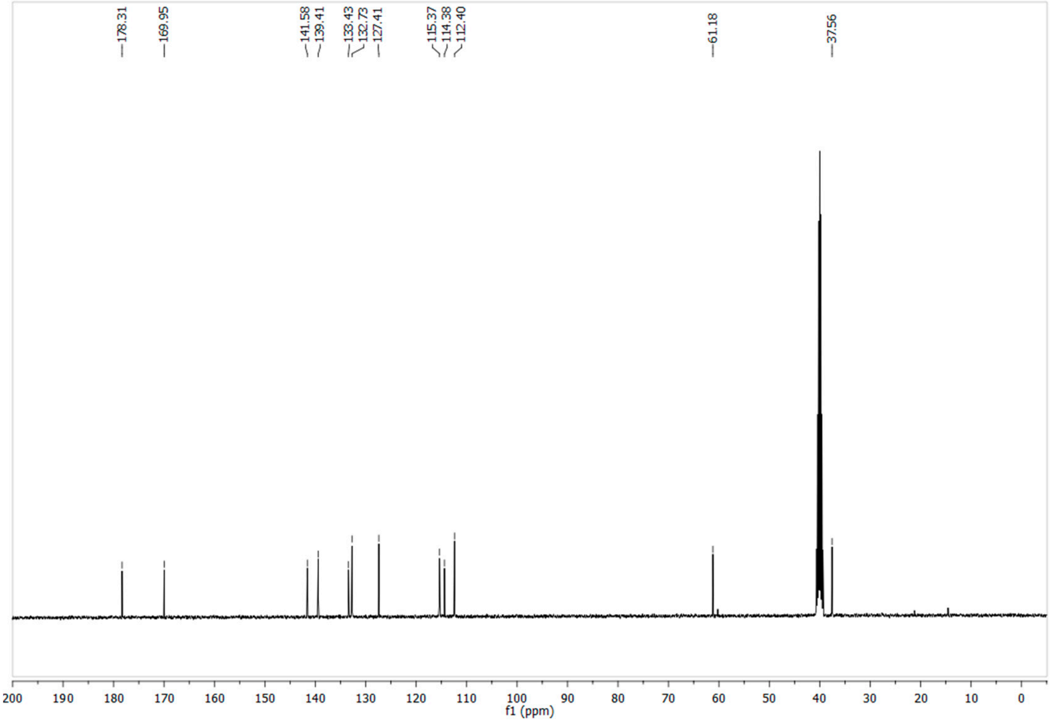

^{13}C NMR of compound **5f** at 101 MHz (DMSO-d_6)

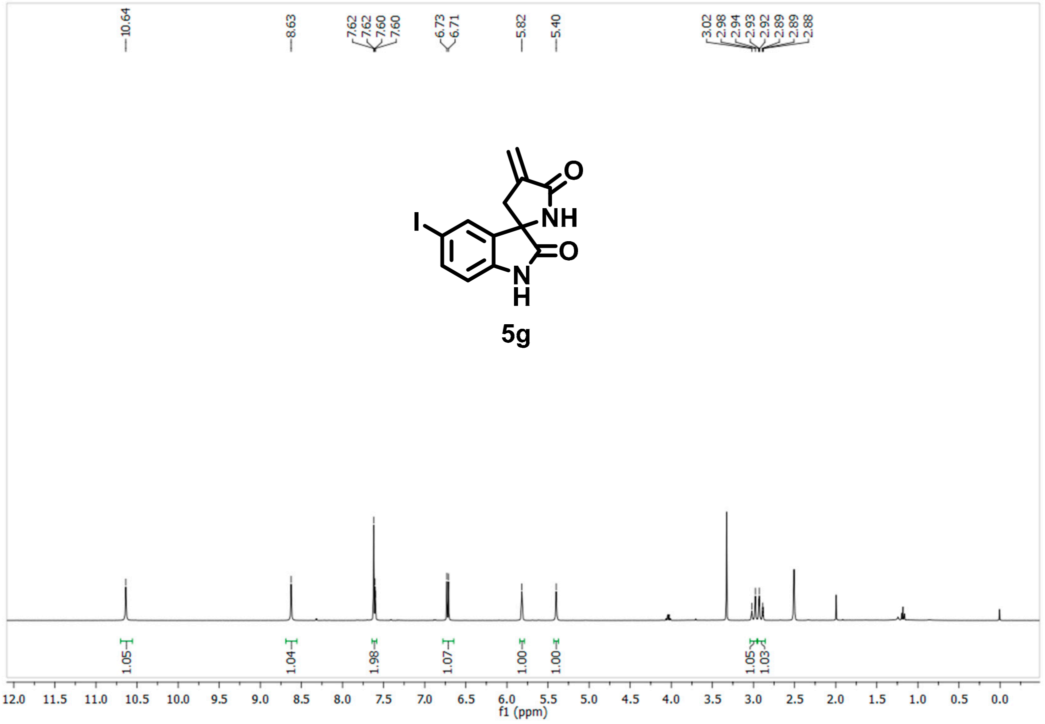

^{1}H NMR of compound **5g** at 400 MHz (DMSO-d_6)

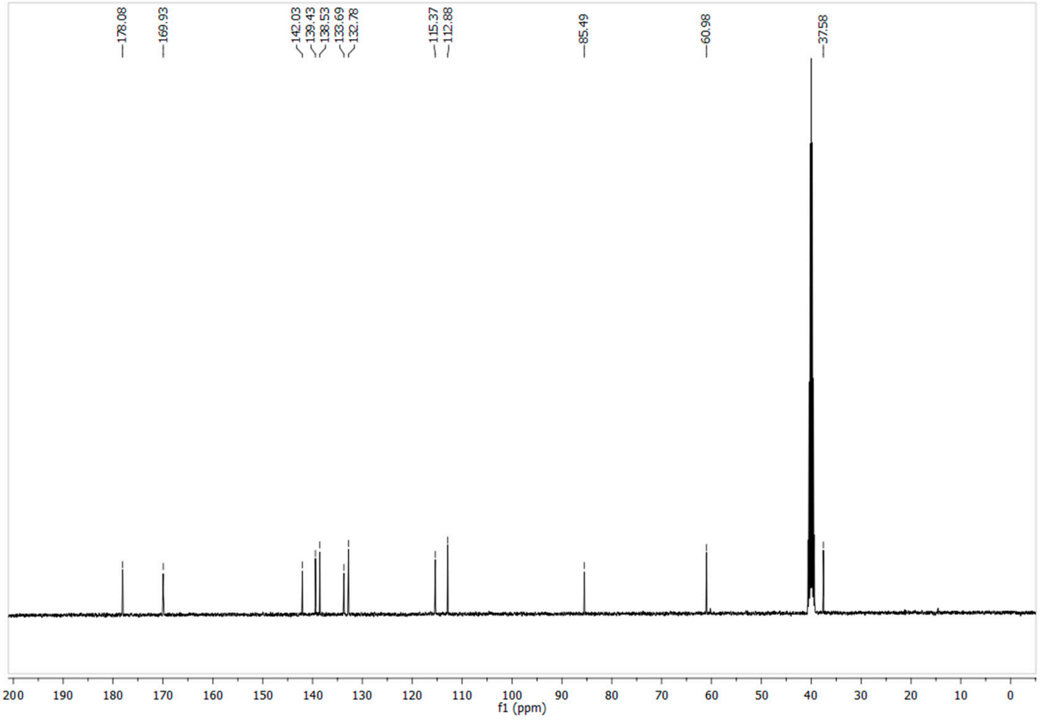

^{13}C NMR of compound **5g** at 101 MHz (DMSO-d_6)

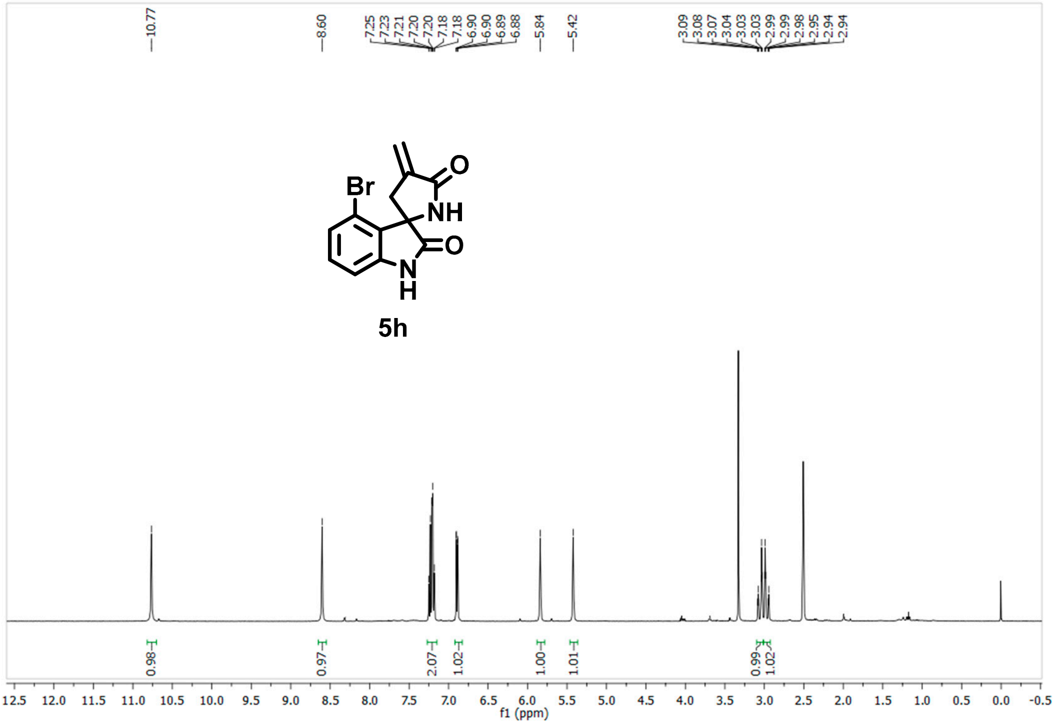

^{1}H NMR of compound **5h** at 400 MHz (DMSO-d_6)

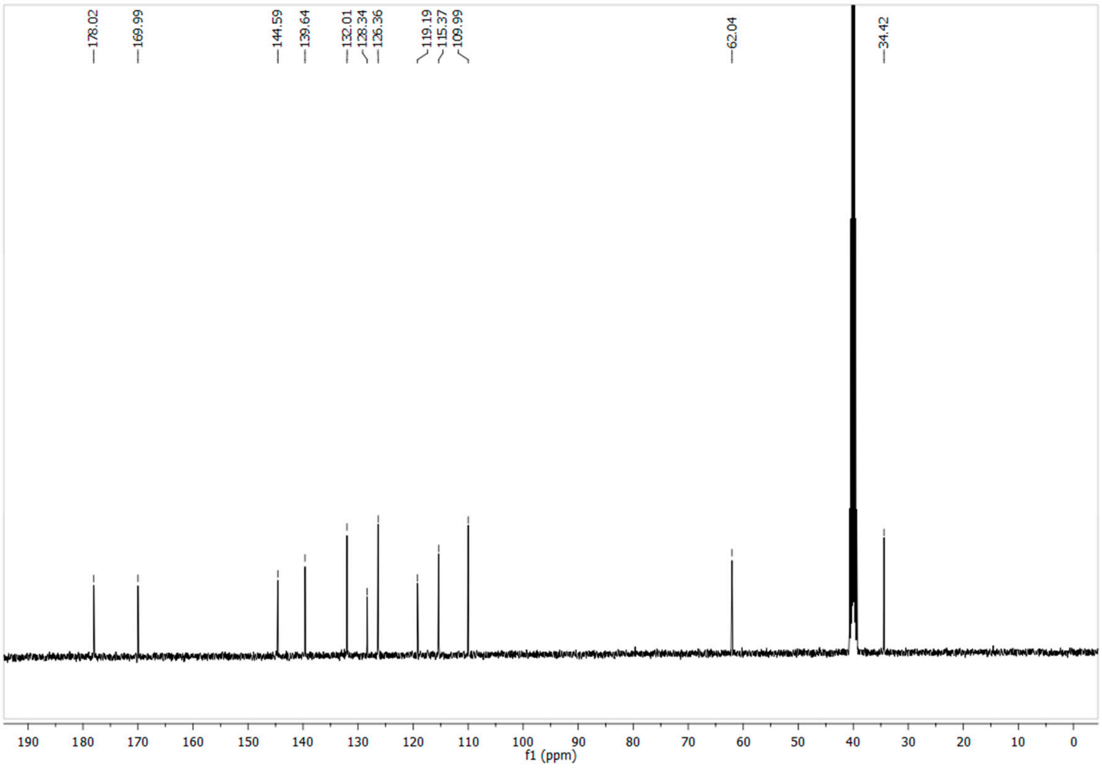

^{13}C NMR of compound **5h** at 101 MHz (DMSO-d_6)

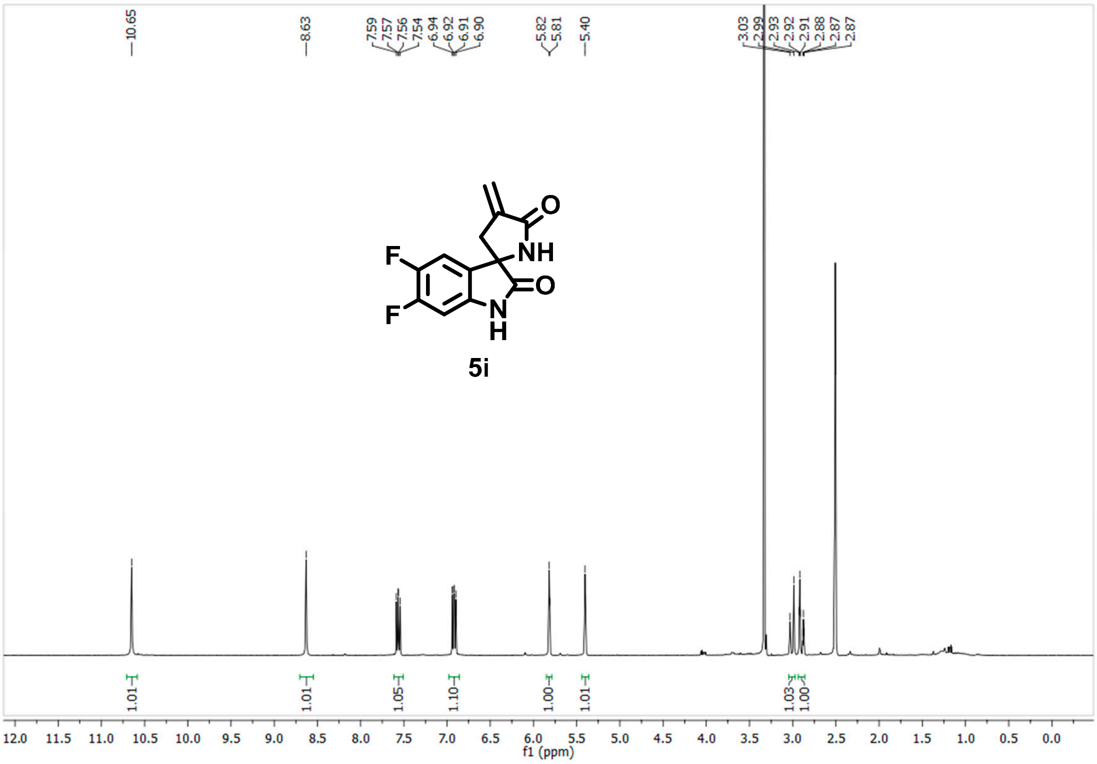

^{1}H NMR of compound **5i** at 400 MHz (DMSO-d_6)

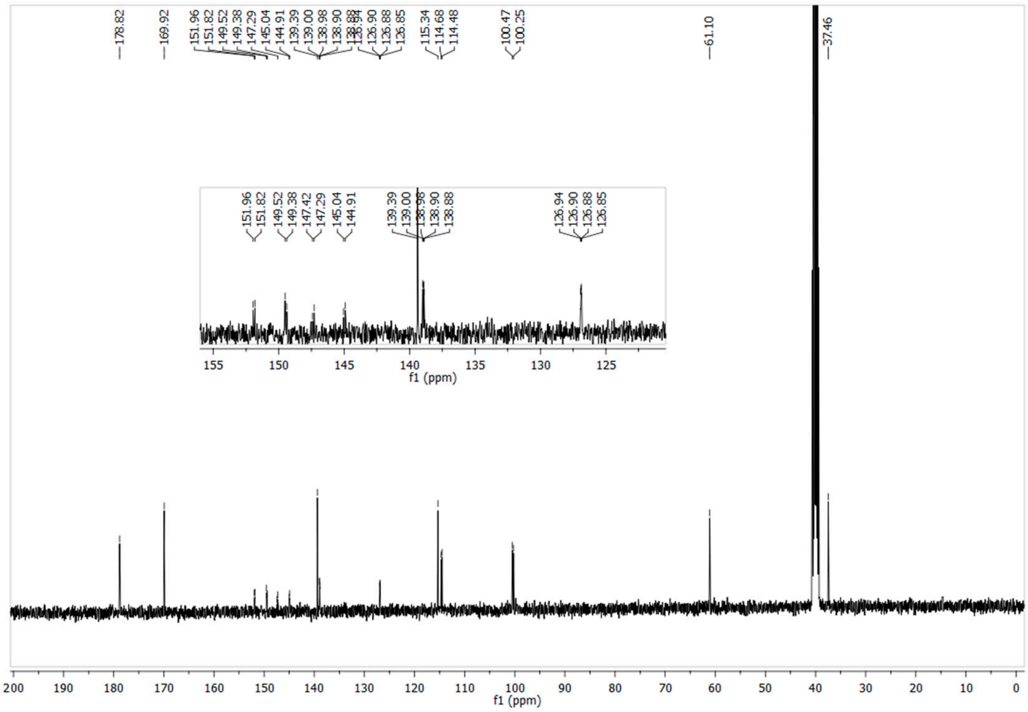

^{13}C NMR of compound **5i** at 101 MHz (DMSO-d_6)

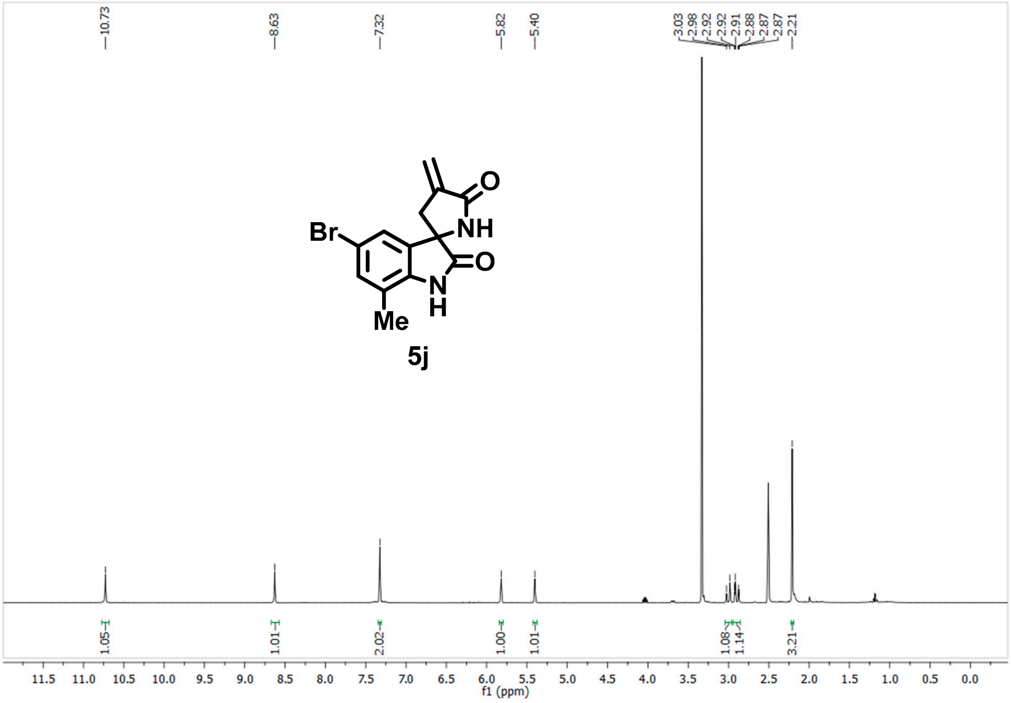

^{1}H NMR of compound **5j** at 400 MHz (DMSO-d_6)

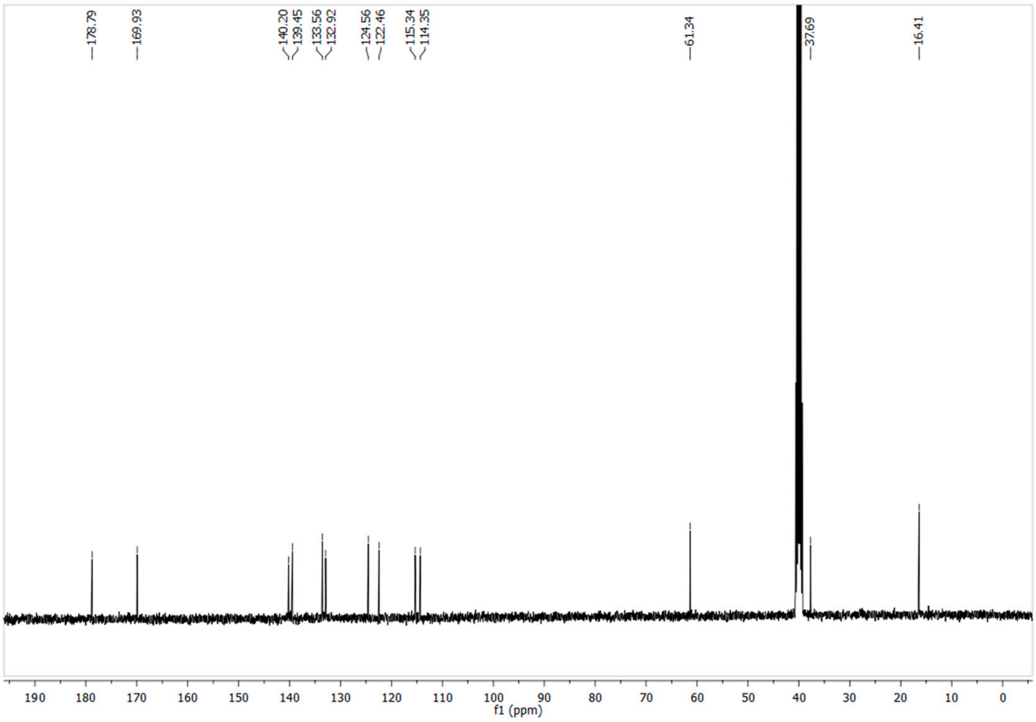

^{13}C NMR of compound **5j** at 101 MHz (DMSO-d_6)

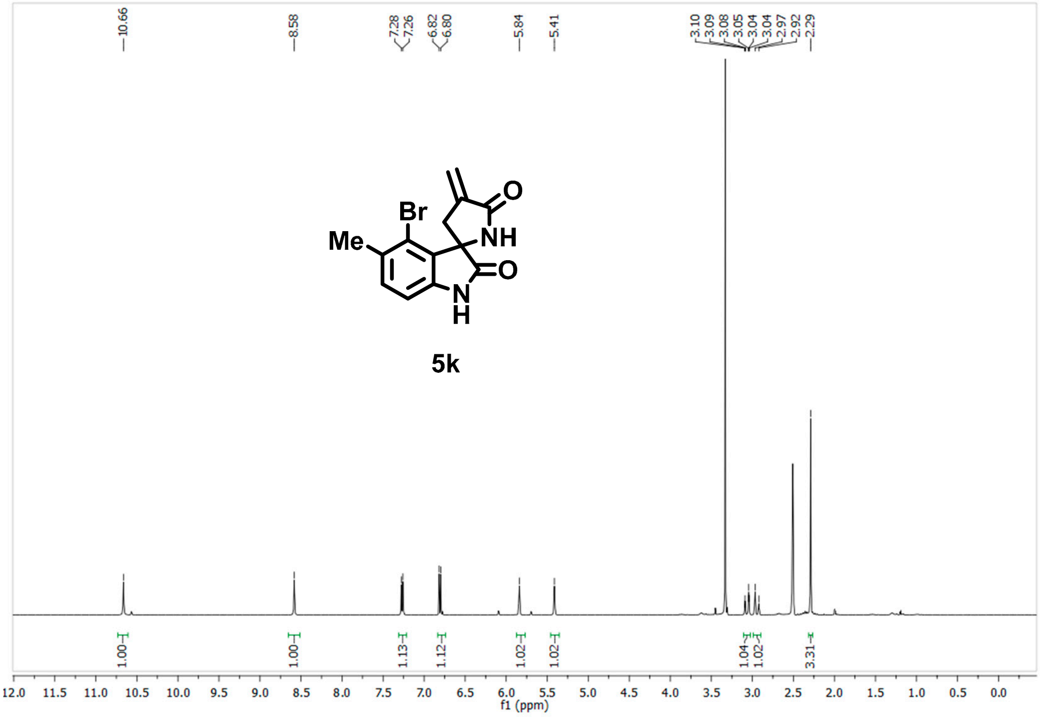

^{1}H NMR of compound **5k** at 400 MHz (DMSO-d_6)

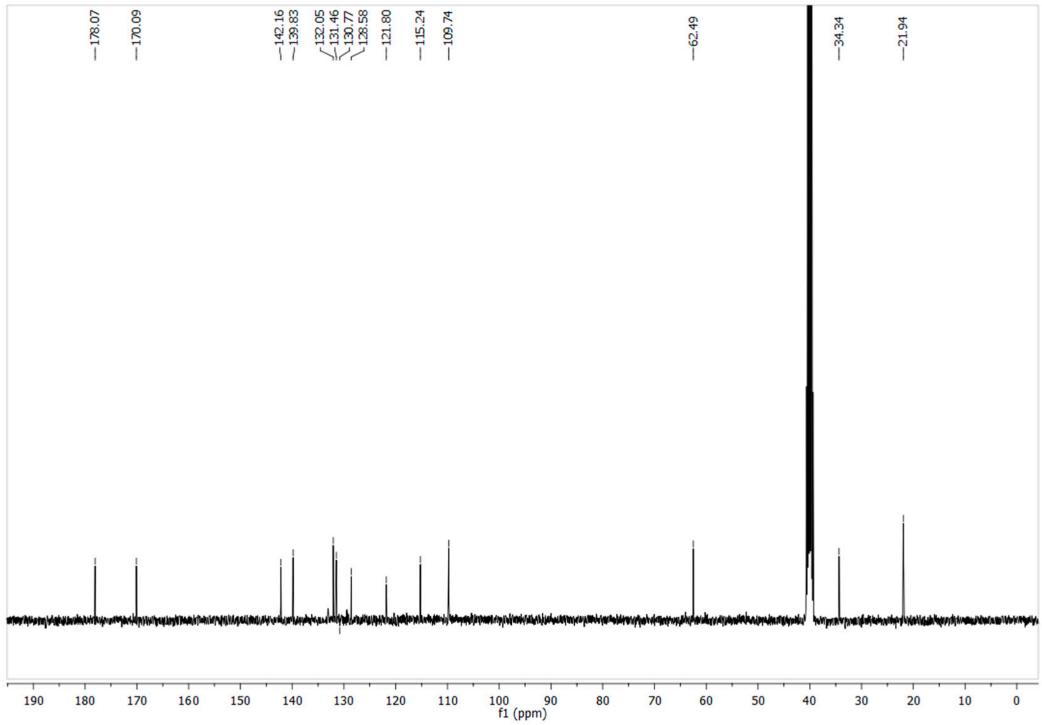

^{13}C NMR of compound **5k** at 101 MHz (DMSO-d_6)

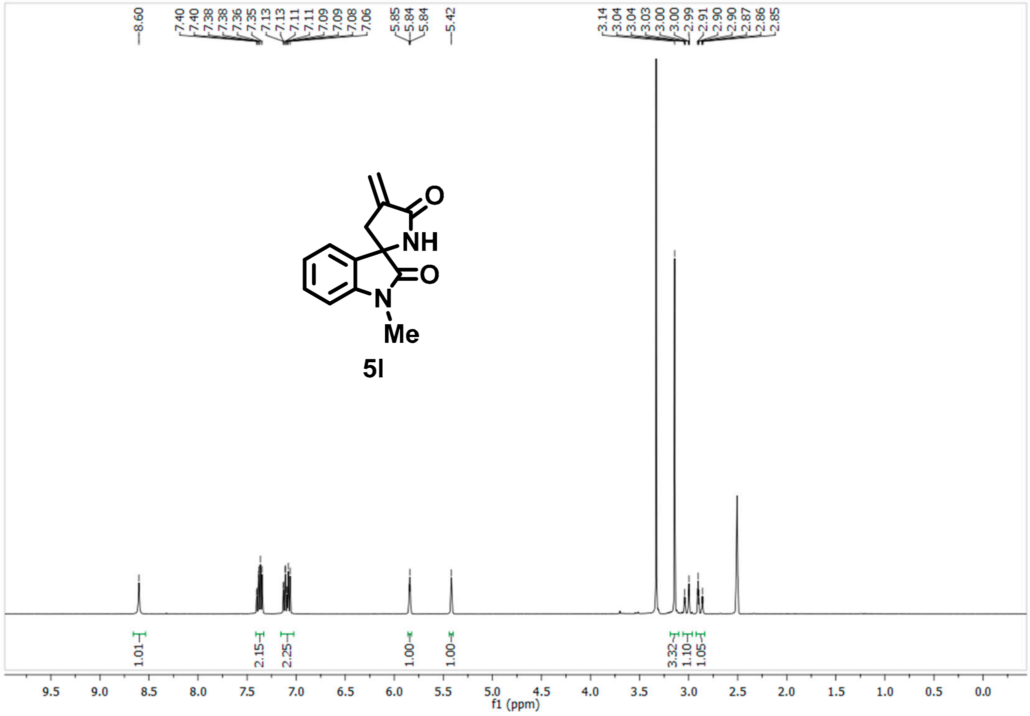

^{1}H NMR of compound **5l** at 400 MHz (DMSO-d_6)

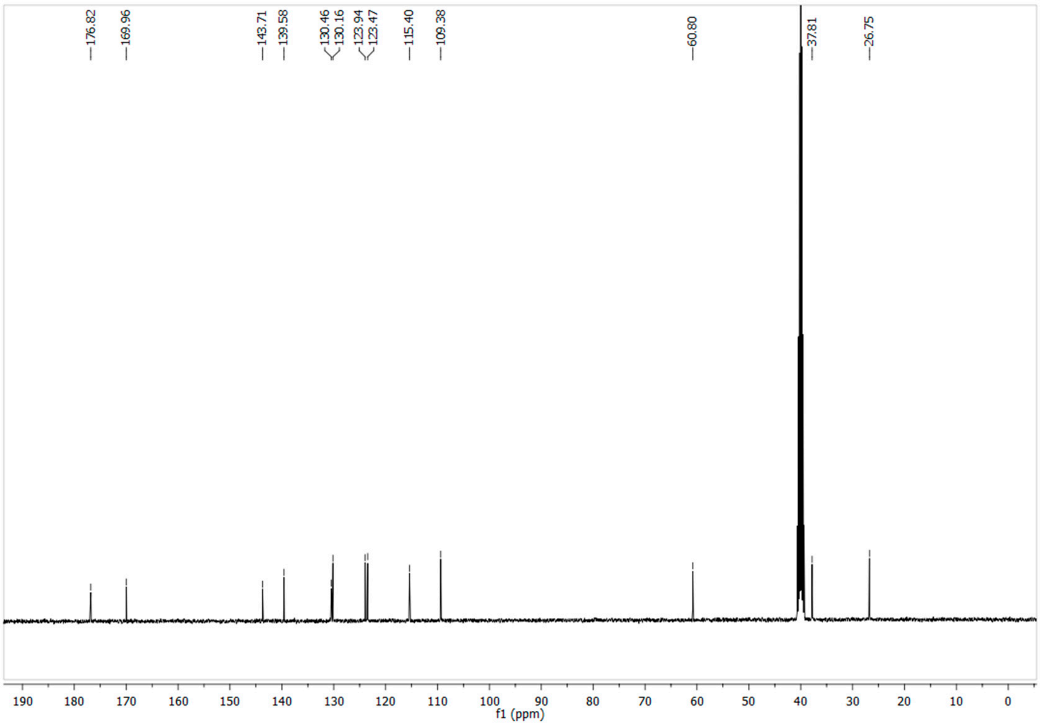

^{13}C NMR of compound **5l** at 101 MHz (DMSO-d_6)

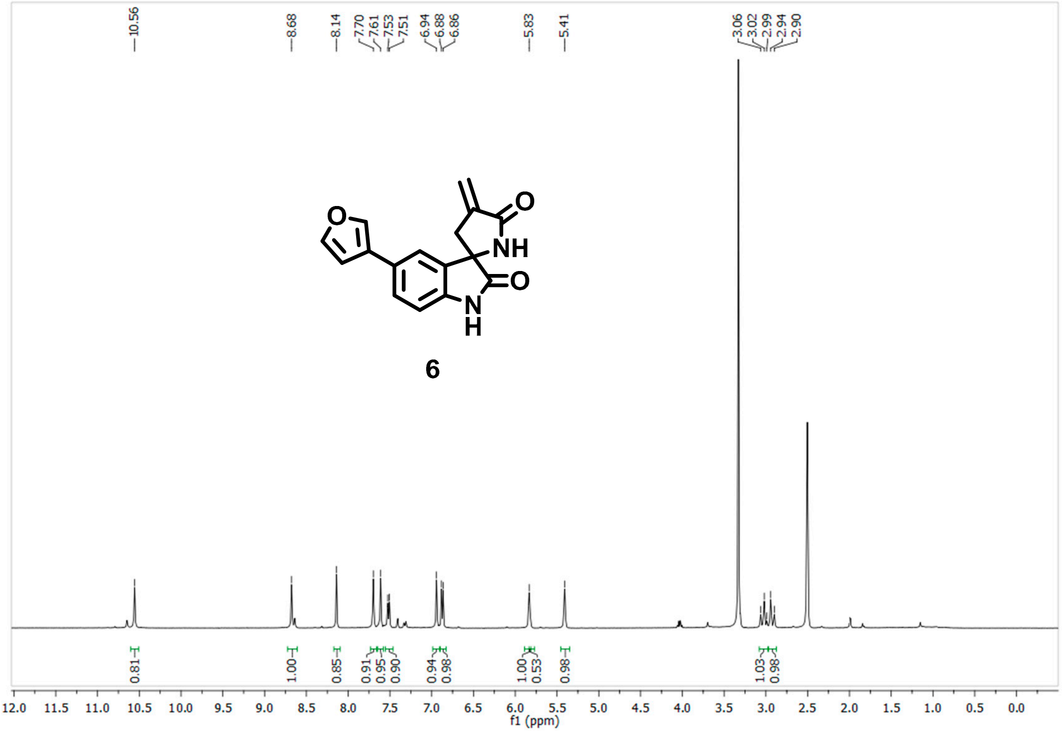

^{1}H NMR of compound **6** at 400 MHz (DMSO-d_6)

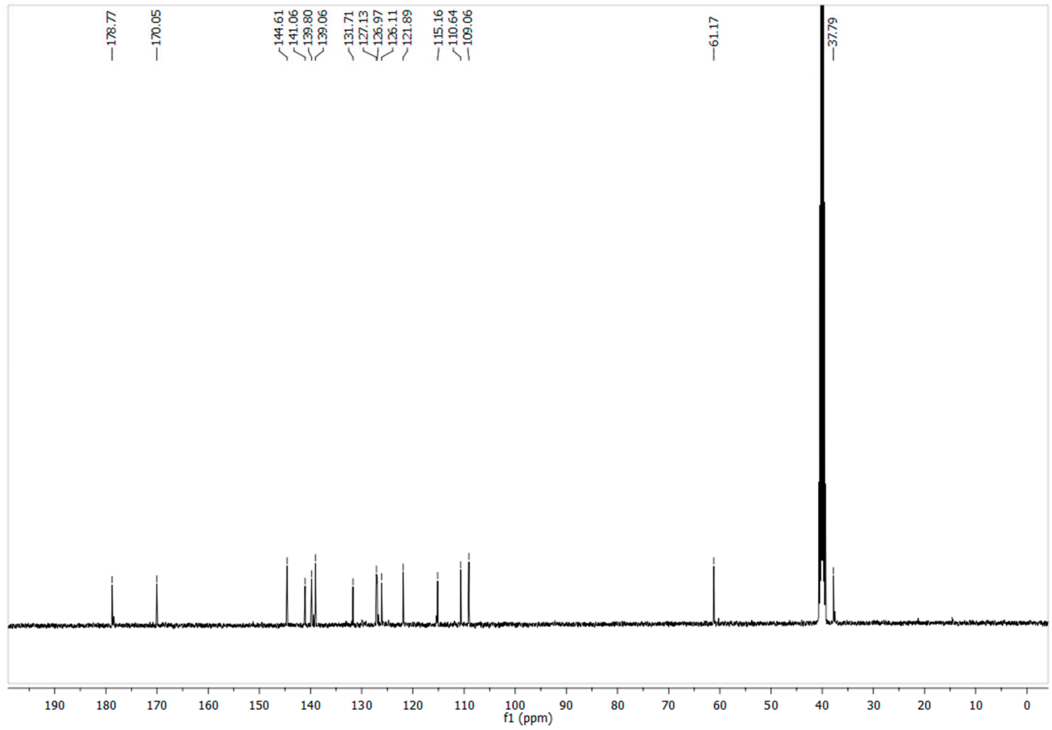

^{13}C NMR of compound **6** at 101 MHz (DMSO-d_6)

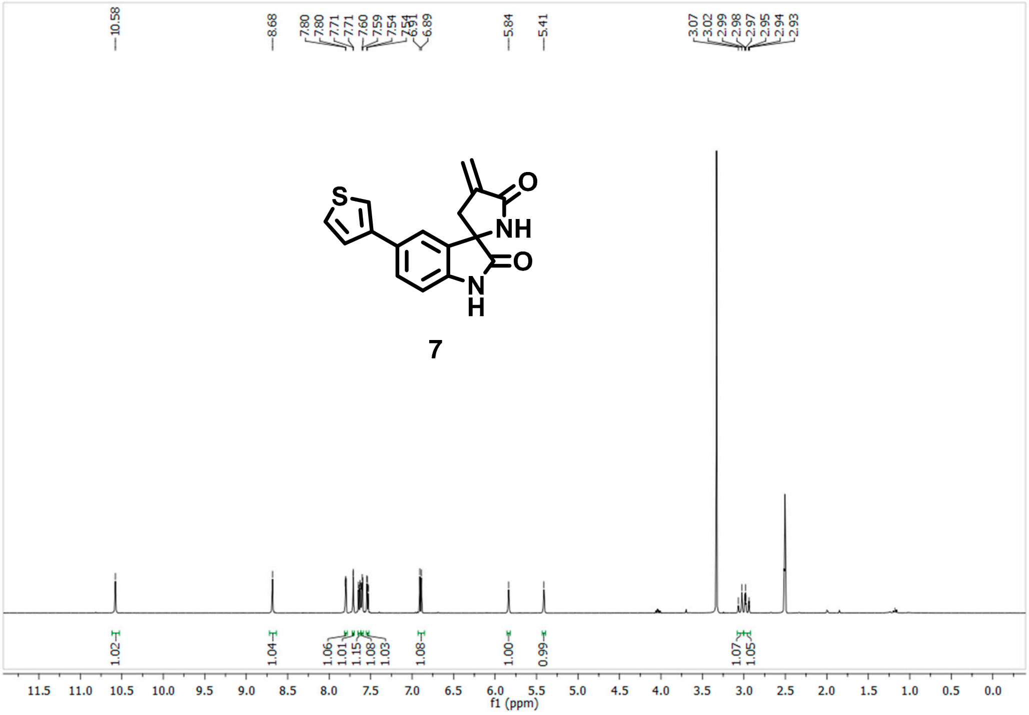

^{1}H NMR of compound **7** at 400 MHz (DMSO-d_6)

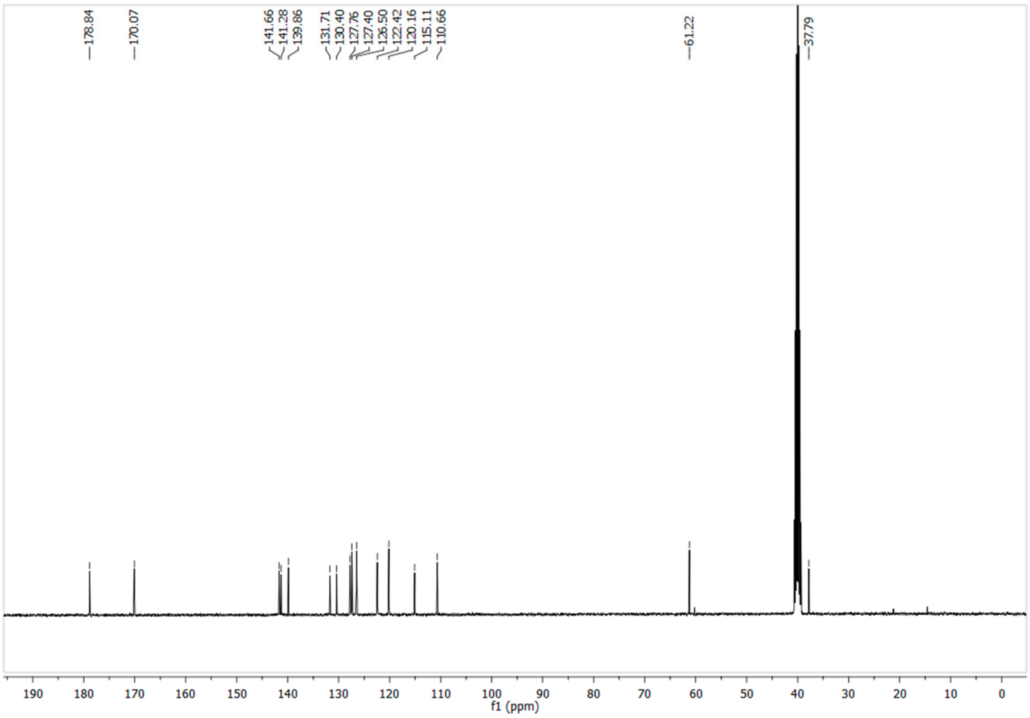

^{13}C NMR of compound **7** at 101 MHz (DMSO-d_6)

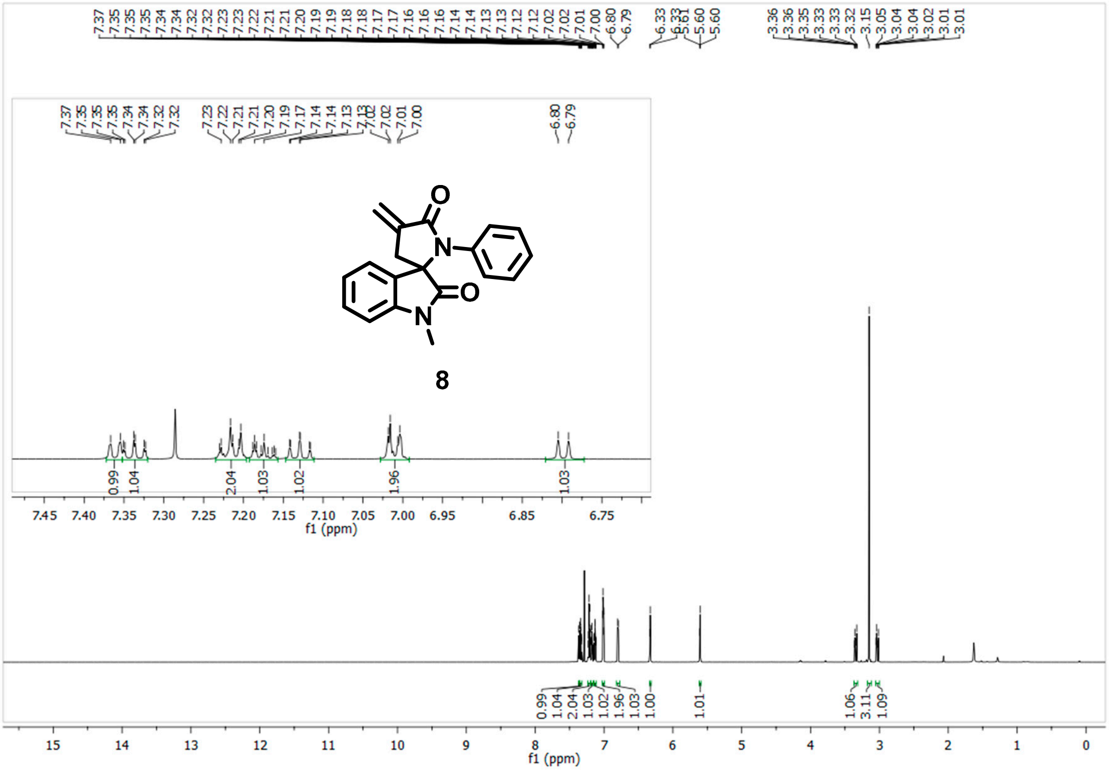

^{1}H NMR of compound **8** at 400 MHz ($CDCl_3$)

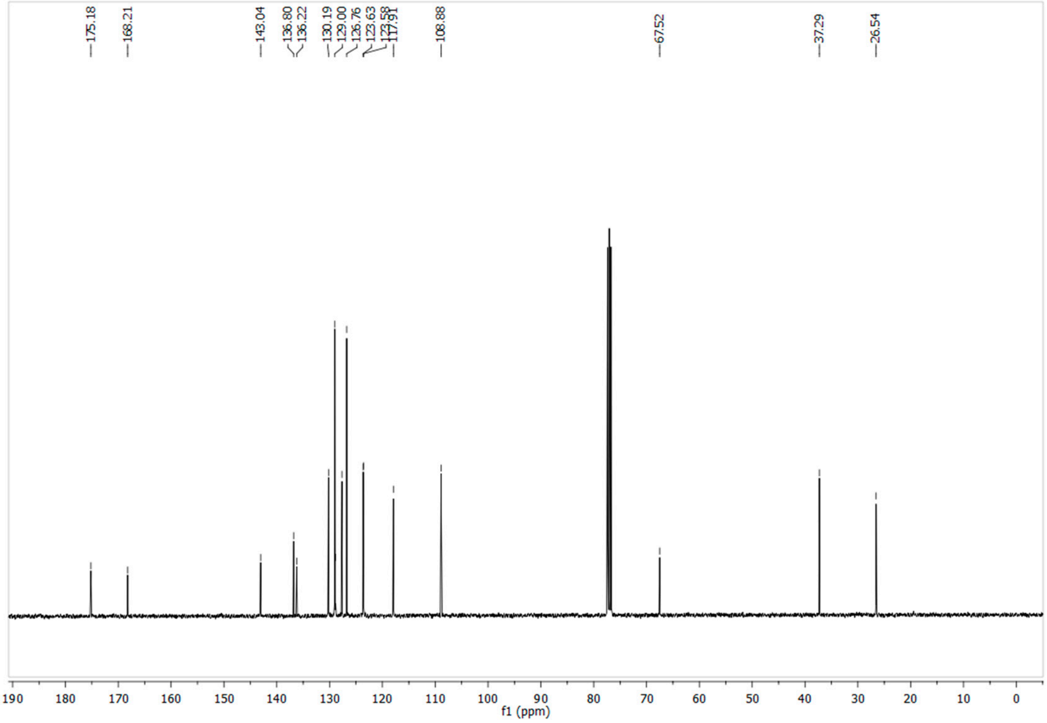

^{13}C NMR of compound **8** at 101 MHz ($CDCl_3$)
